# Prosocial Behavior and Subjective Insecurity in Violent Contexts: Field Experiments

**DOI:** 10.1371/journal.pone.0158878

**Published:** 2016-07-29

**Authors:** María Alejandra Vélez, Carlos Andres Trujillo, Lina Moros, Clemente Forero

**Affiliations:** School of Management, Universidad de los Andes, Bogotá, Colombia; Middlesex University, UNITED KINGDOM

## Abstract

Subjective insecurity is a key determinant of different forms of prosocial behavior. In Study 1, we used field experiments with farmers in Colombian villages exposed to different levels of violence to investigate how individual perceptions of insecurity affect cooperation, trust, reciprocity and altruism. To do so, we developed a cognitive-affective measure of subjective insecurity. We found that subjective insecurity has a negative effect on cooperation but influences trust and altruism positively. In Study 2, carried out three years after Study 1, we repeated the initial design with additional measures of victimization. Our goal was to relate subjective insecurity with actual victimization. The findings of Study 2 support the initial results, and are robust and consistent for cooperative behavior and trust when including victimization as a mediator. Different indicators of victimization are positively correlated with subjective insecurity and an aggregate index of victimization has a negative effect on cooperation but exerts a positive influence on trust.

## Introduction

Research on collective action and social capital based on laboratory experiments and field studies has examined different antecedents of cooperative and other prosocial behavior such as trust and altruism (e.g.[1, 2, 3, 4]) In fact, a comprehensive model that includes all possible determinants seems impossible to devise (See [5, 6]) for a discussion of this point). As suggested by Ostrom and Walker [7] for trust, but also relevant for other prosocial behavior, “*trusting and trustworthy behavior are not unchanging*, *universal attributes of all individuals but are rather the result of multiple contextual and individual attributes*”.

Among these contextual attributes, recent studies have explored exposure to violence, in the context of civil wars, as a focal category, examining in particular the direct relationship between exposure to violent acts and prosocial behaviors. However, conclusions are divergent [8, 9, 10, 11, 12, 13, 14]. In these studies an important variable has been left unexplored: the subjective experience of exposure to violence. What is the relationship between subjective insecurity and cooperative and other prosocial behaviors? There is a fundamental flaw in connecting context level facts (e.g., statistics on violent acts) with individual behavior. This is an important issue because prosocial behavior is potentially a vehicle to overcome the harsh consequences of violence for social dynamics.

We posit that the inconclusiveness of previous studies may be attributable to a lack of research on the mechanisms that link objective violent facts and individual preferences and behaviors. Accordingly, we argue that subjective or perceived insecurity mediates the effect of individual exposure to violence (e.g. victimization) on cooperative behavior and other prosocial behaviors. Our rationale is that violent acts elicit heterogeneous responses among people, due to subjective differences in the ways they experience such situations. That is, individuals experiencing similar conditions of violence in their environment may develop different perceptions of insecurity.

Thus, the focus on subjective perceptions of insecurity is useful for at least two reasons. First, the distinction between objective violence and subjective (perceived) measures of insecurity is needed because objective conditions and subjective perceptions may differ. As Bar-Tal and Jacobson [15] explained, *“individuals perceive external events and conditions*, *evaluate them*, *and subsequently form beliefs about the state of security*. *Estimation of security is thus a cognitive process based on the repertoire of personal beliefs that make up people’s subjective view of reality*. *This implies that external events are subjectively identified*, *interpreted*, *and understood”*. Thus, the same external event might elicit different insecurity beliefs [15] or, as Owen [16] suggests, a person may feel insecure even when objective indicators appear to be favorable. Such perceptions of insecurity, like any other beliefs, are dynamic and are therefore affected and updated by evolving events and the actions of the individual (e.g. [17]). In consequence, a static indicator of violence will not necessarily coincide with subjective perceptions that are the outcome of complex dynamic processes of belief updating.

Second, measures of subjective perceptions of insecurity reflect a wide psychological mindset that may exert an influence on choices and behaviors [18, 19]. As Diprose [18] explains, *“the threat of violence is an important aspect of security and safety; however*, *threats can be real and perceived*, *incorporating many other psychological elements”*. For this reason, the focus on individual perceptions of insecurity allows us to analyze individual-level variables, resolving different problems faced by previous work on violence and pro-social behaviors. For instance, aggregate measures of objective violence are calculated for geographic regions, making it difficult to argue for a causal direction of the relationship between violence and pro-social preferences. It is not clear whether communities develop higher levels of prosocial behavior because of their exposure to violence, or whether they experience more violence because of higher levels of social capital; indeed, in the context of civil war, armed groups might intentionally target specific communities with higher levels of social capital [20].

As an alternative, individual measures of victimization have been used, but these are very hard to capture and are often underreported [21, 22]. Psychological theory (e.g. the Theory of Reasoned Action, [23]), establishes that attitudes similar to subjective insecurity are composed of cognitions and affective associations that precede observable behaviors and behavioral intentions. As Tadjbakhsh and Chenoy [19] expresses it, *“people’s perceptions of security impact on their optimism and pessimism and influence their choices and courses of action*, *and ultimately impact on their lives”*. These arguments support the notion that subjective insecurity influences behavior and that such relationship remains true regardless of the causal direction between prosocial behavior and actual violence.

Although the literature contains studies on subjective perceptions of insecurity as antecedents of wellbeing and associativity [24–27], the influence of subjective insecurity on cooperative behavior, trust, reciprocity and altruism has not yet been examined. These concepts, discussed in the next section, have been analyzed from different perspectives in the literature on collective action and social capital. For some authors, reciprocity and altruism are modeled as social or other-regarding preferences that determine cooperation and trust [28,29]. Others have analyzed the role of reciprocity and trust as forms of social capital that facilitate collective action and cooperative behavior [7]. In fact, some literature identifies cooperative behavior as a result of or even as a proxy for social capital (see, for example, [7, 30, 31]). In this study, we don’t explore the relationships between these concepts, using instead a broad category (“prosocial behavior”) to describe them. As suggested by Batson and Powell [32] the concept of prosocial behavior “*was created by social scientists as an antonym for antisocial*. *Prosocial behavior covers the broad range of actions intended to benefit one or more people other than oneself*”.

In Study 1, we investigated whether individual perceptions of insecurity affect cooperation, trust, reciprocity and altruism, using field experiments carried out with 796 farmers in rural Colombian villages exposed to different levels of violence in recent years. We developed a cognitive-affective measure of subjective insecurity to capture individual perceptions of insecurity for the personal, family and community dimensions. Subjective insecurity captures the way victims or non-victims encode violence in their beliefs and affective associations, in turn influencing pro-social behavior. Our measure of subjective insecurity is based on developments of the concept of human security [33] and in particular the definition proposed by Tadjbakhsh [34]: “*Insecurity can refer to the loss of the guarantee of access to jobs*, *health care*, *social welfare*, *education*, *etc*. *as much as to the fear that arises from domestic violence*, *political instability*, *crime*, *displacement*, *etc*.”. As such, our insecurity measure, explained further in the methods section, captures an individual-centered concept in which “*threats depend invariably on the context and can be anything from a sudden clear and present danger to a chronic violation of human dignity*” [34]. In fact, people are rarely able accurately to trace, let alone verbally explain, the causes of such complex perceptions. We found that subjective insecurity has a negative effect on cooperation but a positive influence on trust and altruism, and no effect on reciprocity.

Three years after Study 1, we returned to the same villages and replicated the same experimental design with 800 farmers, applying in addition a new measure of victimization. Our goal was to relate subjective insecurity with individual accounts of exposure to violence, using reports of actual victimization. Thus, with individual-level variables of victimization and subjective insecurity we were better able to assess the appropriateness of a mediating model of the relationship between victimization and prosocial behavior through the subjective perceptions of insecurity.

In Study 2 we found consistent results for cooperative behavior and trust when controlling for victimization. However, subjective insecurity does not have a significant effect on reciprocity and loses its effect on altruism. We also found that, although all types of victimization are positively correlated with subjective insecurity, the effect of victimization on cooperation and trust could be either negative or positive depending on victimization type. Furthermore, we found that direct and indirect paths for the link between victimization and pro-social behavior could operate in opposite directions.

Th**e** research findings extend knowledge about the contextual determinants of pro-social behavior. Our findings also highlight the richness and complexity of the ways in which **v**iolence influences individual behavior; in addition they open up new questions for the exploration of the behavioral mechanisms behind these relationships. Most importantly, our findings indicate new directions for social interventions aimed at recovering individual agency and fostering community cooperation to overcome collective action problems. In particular, intervention programs intended to reduce violence should consider an eventual lag between actual violence reduction and the effective decrease of subjective insecurity, resulting in policies that target the perceptions of threats to security and safety successfully, both in the present and in the future.

## Related Literature

During the past two decades the experimental and behavioral economics literature has collected an extensive amount of evidence criticizing self-interest as the only motive driving individual behavior [1–3]. In this context, prosocial or other-regarding preferences have been identified as additional important drivers. Two (out of many) important social preferences discussed in the behavioral literature are reciprocity and altruism (e.g. [35, 36, 37]). Reciprocity implies “*conditional kindness*” [35, 36] whereas altruism, is “*a form of unconditional kindness*” [35]. Neither is driven by an expectation of future benefits but both, and in particular reciprocity, are important drivers of cooperation, understood as “*individual behavior that incurs personal costs in order to engage in a joint activity that confers benefits exceeding these costs to other members of one’s group*” [38]. Ostrom and Walker [4, 5] suggest that reciprocal individuals should prevail if trust is to develop. Trust, then, is considered “*a matter of the beliefs that one agent has about the behavior of another*” [36]. In the behavioral model developed by Ostrom and Walker [4, 5] the mutually reinforcing relationships between reciprocity, reputation and trust are determinant to explain cooperative behavior. And, as suggested by them [4, 5], these relationships are affected by structural and contextual variables (e.g. violence).

Some recent experimental studies have explored the relationship between objective violence, measured by intensity indicators (e.g. victimization, homicides rates, number of kidnappings, attacks, displacement), and prosocial preferences such as trust, altruism and cooperation. To date, however, the evidence concerning the direction and nature of the relationship between exposure to violence and pro-social behaviors is not conclusive. The sign and direction of causality is still a matter of disagreement (see for example [9, 10–14, 39]). As summarized by Gilligan et al. [10], these studies put forward different explanations of their results: a) a preference-based hypothesis, which posits that individuals actually change their preferences as a result of their exposure to violence; b) an institutional explanation, which suggests that individuals develop and adopt new social norms to cope with violence, without changing their preferences; and c) a purging hypothesis, which proposes that individuals with certain preferences or conditions leave their communities as a consequence of violence, changing the distribution of social preferences in a given community but causing no change in individual preferences.

Voors et al. [9] sought to establish the causal impact of indicators of civil-war victimization on social, time and risk preferences. They found that individuals self-reporting high levels of exposure to violence display more altruistic behavior, are more willing to take risks, and exhibit a higher discount rate, but that victims and non-victims do not differ very much in related prosocial behaviors. Similarly, Gilligan et al. [10] found that subjects from villages exposed to violent conflict were more likely to contribute in public goods games and were more trusting than subjects from villages not exposed. However, subjects from victimized households, in spite of being more altruistic, were not more likely to contribute to public goods, nor were they more trusting than subjects from non-victimized households. Explanations of these results based on psychological recovery during post-conflict periods have not been conclusive. Victims’ prosocial behavior has also been found to be contradictory: they participate more in community activities but are less trusting [11]. In addition, victims display increased risk aversion [39] but their contribution in public goods games is higher [12]. Further evidence shows that victims’ increased involvement in community activities is accompanied by decreased cooperation in public goods games [40, 41]. Finally, Whitt and Wilson [42] use dictator games to study the effect of ethnic conflict and culturally inherited rivalries from the war in Bosnia. In spite of in-group bias, they found that in a post-conflict context, the effect of violence is not strong enough to constrain altruistic behaviors, contradicting what might have been expected.

These works attempt to trace a direct link between actual levels of violence observed either at community or individual level (e.g. victimization) and the behavior of individuals. In so doing, they do not incorporate the heterogeneity of subjective individual responses to violence. Hence the specific literature on violence and cooperation also ignores individual-level antecedents. As exposure to a violent act interacts with a myriad of individual psychological conditions, persons may react in different ways to the same objective violent act or threat (e.g., witnessing a murder). We argue that subjective insecurity, a variable that is not explicitly captured by indicators of victimization, is a key determinant of prosocial behavior. In particular, our research expands the hypothesis that preferences might be modified, by proposing a perception-based mechanism that links experience of violence with cooperative behavior, trust, reciprocity and altruism. The vehicle of that link is the heterogeneous subjective insecurity that ensues from the subjective experience of violence.

## Results

### Study 1

We calculated an aggregated index of perceived insecurity as the average of its three dimensions (i.e., personal, family and community). Factor analyses and Cronbach’s alpha (α_personal_ = .81; α_family_ = .85; α_community_ = .82; α_total_ = .92) revealed that for each dimension of insecurity the eight questions loaded on a single factor (see S1 Fig) with high reliability for each insecurity dimension. The reliability coefficients are above the cut-off points suggested by Hair et al. [43]. One item was removed from community insecurity due to very low factor loading (less than 0.05). We conclude that we obtained a reliable overall measure of subjective insecurity.

The difference in the overall mean of subjective insecurity between municipalities of relatively high and low violence was significant (*M*_*high*_ *= 1*.*83*, *M*_*low*_ *= 1*.*50*, *t = -7*.*07; p <* .*001*). This result persisted for each dimension of insecurity, reinforcing the external validity of our measure. There were no differences in subjective insecurity across games (*F = 0*.*54; p > 0*.*5*). Table 1 shows the mean subjective insecurity by dimension and by game, together with the aggregate insecurity index by game. Also worth noting is that the distribution of insecurity was highly skewed (See S2 Fig).

**Table 1:** Summary statistics of perceptions of insecurity by game: Studies 1 and 2.

**Study 1**	**N**	**Personal insecurity**		**Family insecurity**		**Community insecurity**		**Aggregate insecurity**	
		**HV**	**LV**	**HV**	**LV**	**HV**	**LV**	**HV**	**LV**
Public Goods	320	1.93	1.54	1.86	1.43	1.88	1.51	1.89 (0.71)	1.49 (0.60)
Trust	158	1.74	1.56	1.67	1.43	1.66	1.51	1.69 (0.70)	1.51 (0.59)
Reciprocity	158	1.85	1.55	1.86	1.41	1.89	1.53	1.86 (0.72)	1.49 (0.58)
Dictator	160	1.82	1.58	1.85	1.52	1.75	1.49	1.81 (0.63)	1.53 (0.67)
**Study 2**									
Public Goods	320	1.72	1.42	1.81	1.67	1.75	1.53	1.76 (0.48)	1.54 (0.38)
Trust	160	1.75	1.63	1.86	1.71	1.71	1.64	1.78 (0.49)	1.65 (0.53)
Reciprocity	160	1.82	1.44	1.89	1.72	1.87	1.55	1.86 (0.59)	1.57 (0.41)
Dictator	160	1.75	1.44	1.89	1.68	1.75	1.55	1.81 (0.49)	1.56 (0.43)

Note: This table presents summary statistics of perceptions of insecurity by game, per study and for each level of violence: high violence (HV) and low violence (LV). We asked the same eight questions at the personal, family (household) and community levels to capture variations of subjective insecurity as a function of social distance (i.e., distance from the self). Aggregate insecurity is the average of the three dimensions. Standard errors for aggregate insecurity index are in parentheses.

### Public Goods Game

**Fig 1:**
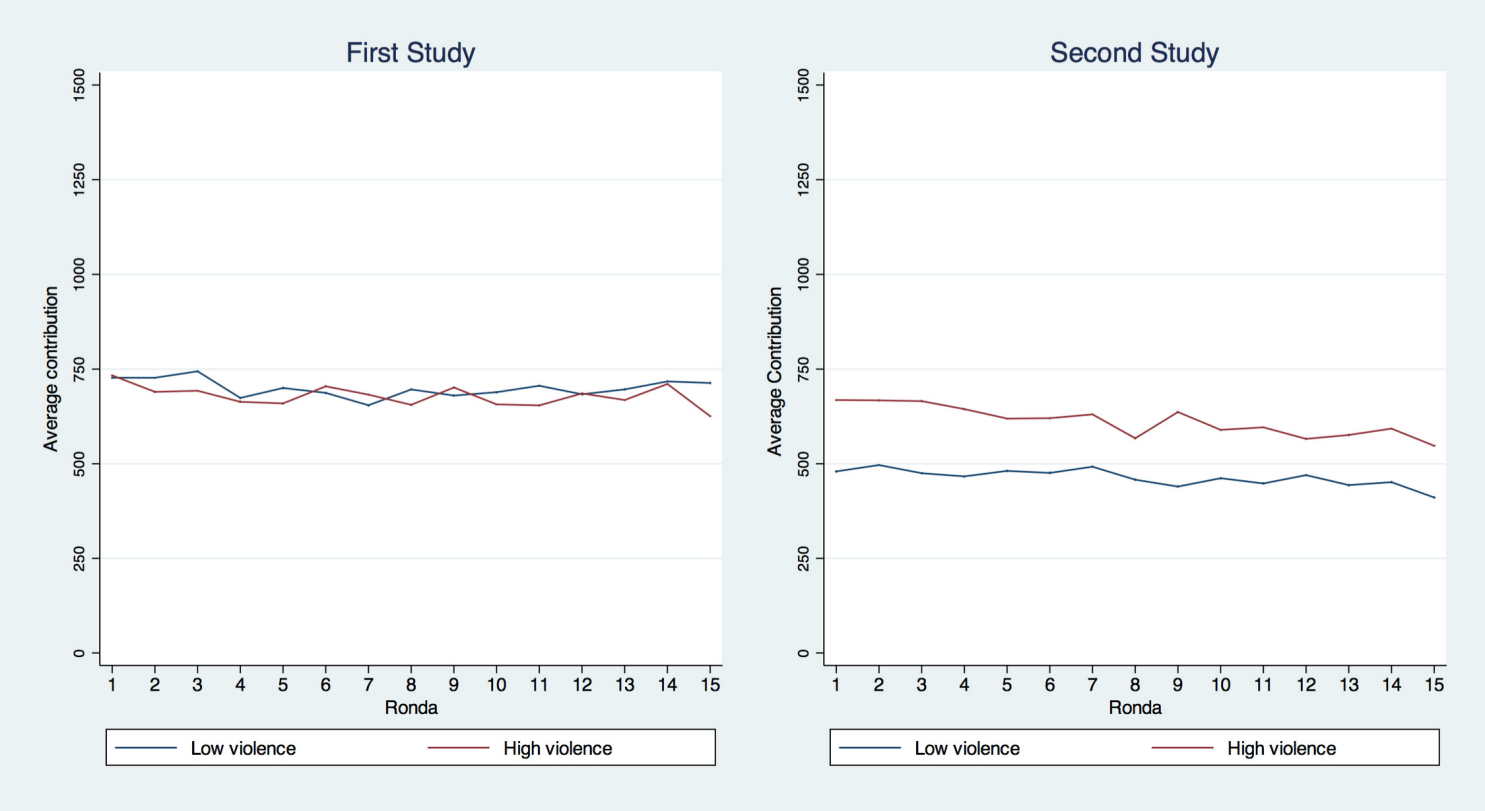
Average contribution by round: PGG.

Average cooperation across all rounds and groups was 46%, which is within the range of 40% to 60% reported in the literature [44,45] and in recent public goods games conducted in the same region [40, 41]. No evidence was found either of learning or behavioral change, as rounds progressed in a way that was consistent with previous field experiments [45, 46, 47]. Fig 1 shows average cooperation by round. Cooperation in Round 1 was 48%, slightly decreasing to 45% in Round 15. However, no significant trend was observed (*t = 1*.*7; p >* .*08*). A closer look at the distribution of contribution reveals a bimodality located in the 45^th^ and 75^th^ percentiles, corresponding roughly to a contribution of one third or two thirds of the endowment (See S3 Fig for the overall distribution). The endowment was 1,500 Colombian pesos (COP) for each round. Local currency is available in coins of 50, 100, 200, 500 and 1,000 COP, and bills of 1,000 and 2,000 COP and above. However, during the experiment, subjects were not constrained by these units (i.e. coins and bills of certain denominations). We therefore do not think that the two focal points can be attributed to an artifact of the experiment, but we did adjust our statistical methods to account for this empirical situation.

To explore the relationship between cooperation and subjective insecurity, we conducted quantile regressions analysis in order to account for the bimodality of contributions (the dependent variable) in the public goods game [48]. Given the distribution of the contributions, quantile regressions are more appropriate than linear regressions, as the mean falls between the two modes.

We estimated the effect of subjective insecurity on cooperation using the aggregated index of perceived insecurity as the main independent variable, several socio-demographic covariates, and dummy variables for rural districts in order to control for local fixed effects of community on cooperation, as suggested by the literature on violence and conflict (e.g. [49]). In addition, we used bootstrapped estimation of standard errors and robust clustered errors (2,000 repetitions) to account for the repeated measures in our data (in our experiment, each participant made 15 decisions about contributions). We estimated the regression for quantiles 45 and 75, since preliminary analyses revealed that these were the two most likely values in the distribution of cooperation (See S1 Table).

**Fig 2:**
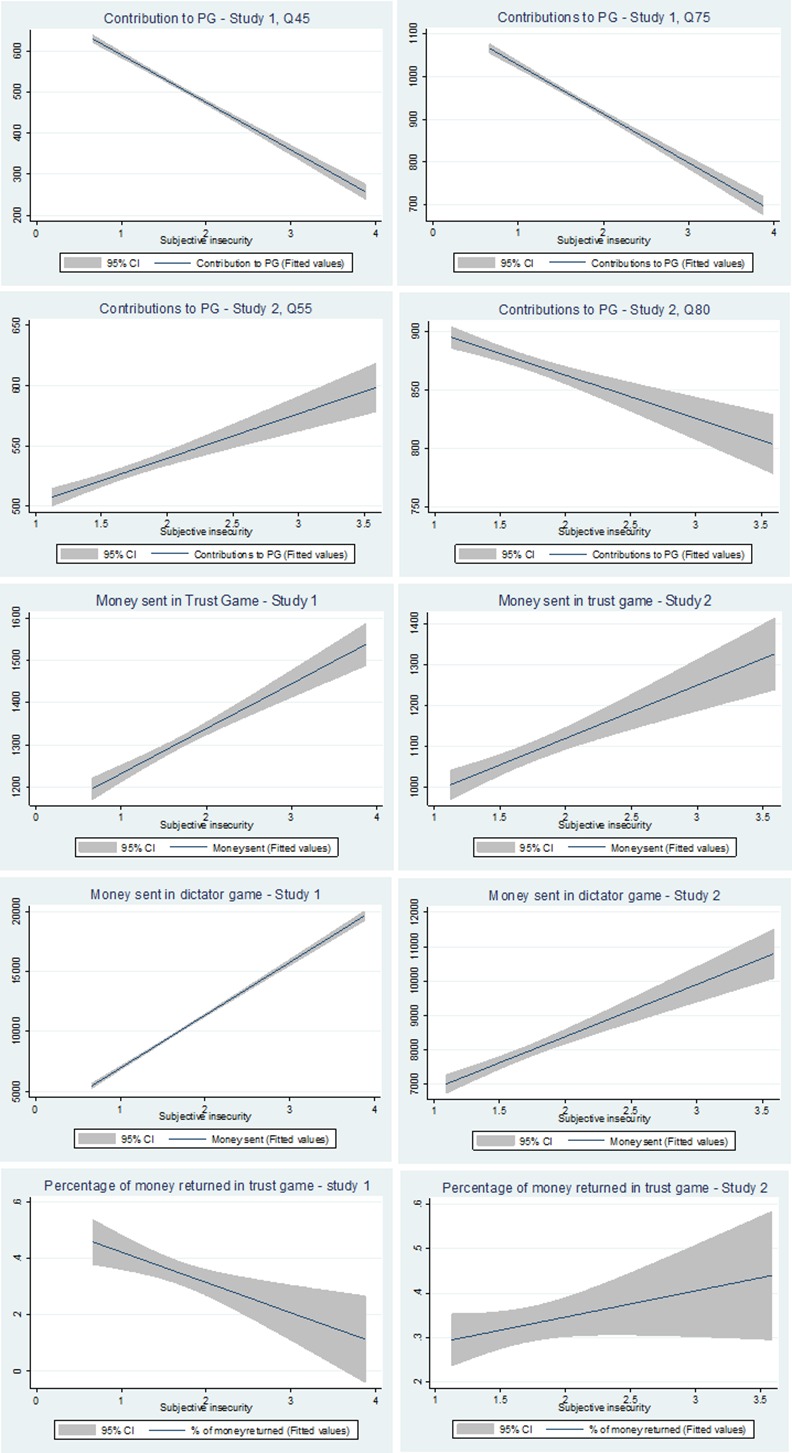
Summary of estimated results for all games. Note on Fig 2: This figure shows the estimated effects of subjective insecurity on each of the pro-social behaviors studied. Contribution (cooperation) in the PGG game was analyzed using simultaneous quantile regressions with robust clustered errors. Money sent (trust) and money returned (reciprocity) in the Trust game were analyzed using linear panel data regressions with population averaged effects and money sent (altruism) in the Dictator game was analyzed using median regressions. For further details, please see the methods section and the Supporting Information.

We found a significant negative main effect of subjective insecurity on cooperation for both quantiles (See Fig 2 for a summary of estimated results for all games). Regarding the covariates that significantly explain cooperation in both quantiles we found that being affiliated to the National Federation of Coffee Growers (FNC) increased contribution to the public good, but that cooperation decreased when people in a game session knew each other. More education significantly reduced contribution for levels of low cooperation (Q45), and men contributed more for levels of high cooperation (Q75). Age was not significant.

### Trust Game

**Fig 3:**
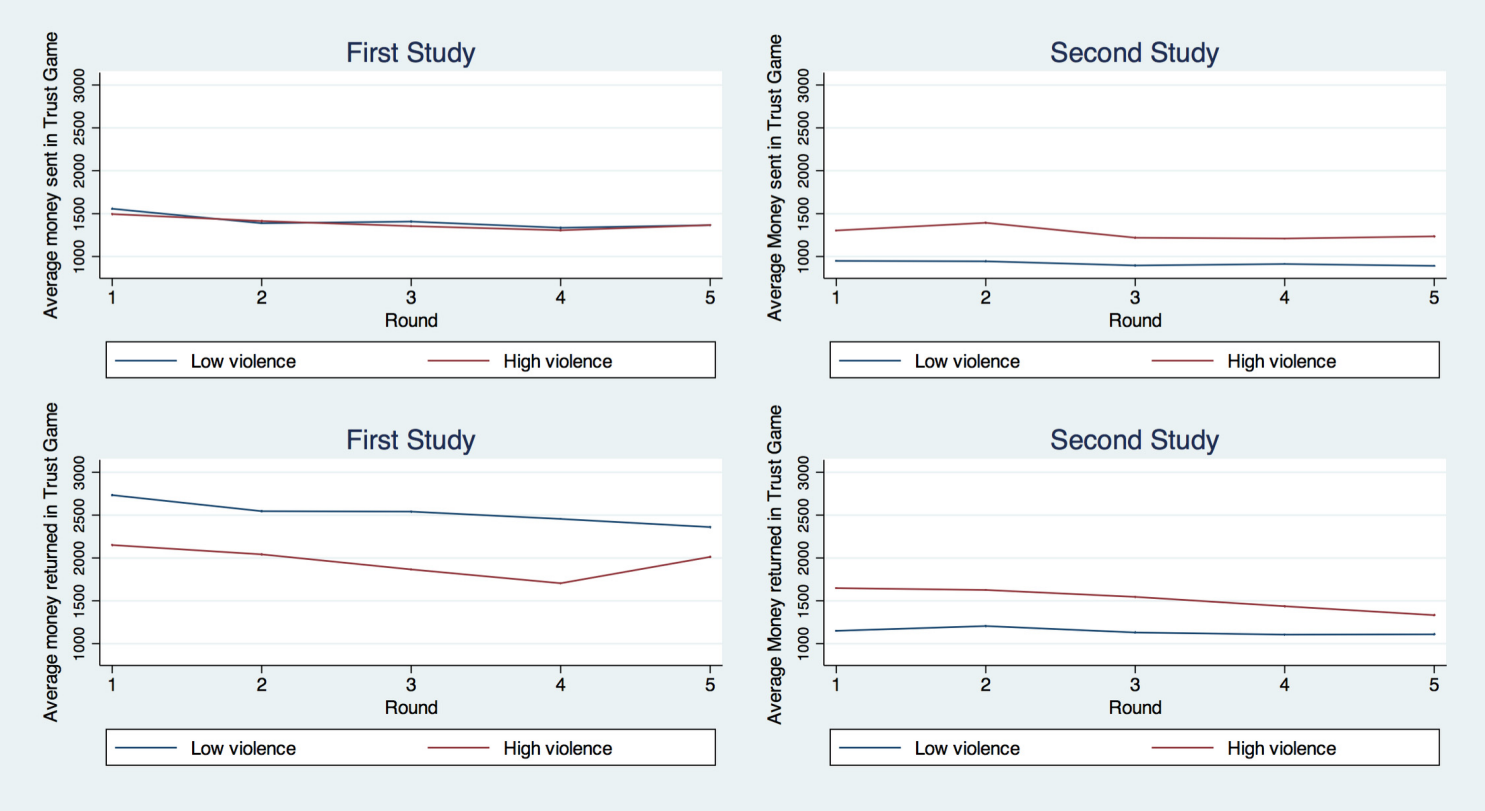
Average money sent and returned: Trust Game.

Money sent in the trust game averaged 46% of the endowment across the five rounds played; this is slightly below the range of 50% to 65% reported in the literature [1,7]. There was a slight decrease in money sent over the rounds (*F = 2*.*04*, *p < 0*.*1*), showing some learning (see Fig 3). The distribution of money sent fell mostly at 6 focal points at specific quantities (See S4 Fig). On the other hand, the average amount returned, which is used as a measure of reciprocity, was 64% of the money sent multiplied by 3, which is outside the range of 30% to 40% found in the literature mentioned above. Note that in our procedure, participants B were allowed to include money from their initial endowment in the amount returned. There was no significant change in this behavior over rounds (*F = 1*.*23; p = 0*.*29*) (see Fig 3). The distribution of money returned fell mostly at 6 focal points and at specific quantities (See S4 Fig).

In order to analyze the effect of subjective insecurity on money sent in the trust game, we conducted a panel linear regression with population-averaged effects (See S1 Table). Though the distribution showed several focal points, there was no benefit in conducting quantile regressions because the average contribution was well captured by a linear specification. We included the same covariates used in the regressions for cooperation. We found that the effect of subjective insecurity on trust was the opposite of the effect observed for cooperation (See Fig 2). That is, subjective insecurity increases trust significantly. Regarding covariates, trust was also negatively related to the number of other participants known by each person who were also taking part in the experiment. There was a significant, and expected, positive effect of the money returned through reciprocity. In contrast to cooperation, no other covariate exerted a significant effect, and there was a small but significant reduction in trust as the rounds progressed. We repeated the same specification for the money returned (reciprocity). However, we found no effect of subjective insecurity on reciprocity. This behavior was affected principally by the money sent by player A. No other covariate affected the money that was sent back (See S1 Table and Fig 2).

### Dictator Game

**Fig 4:**
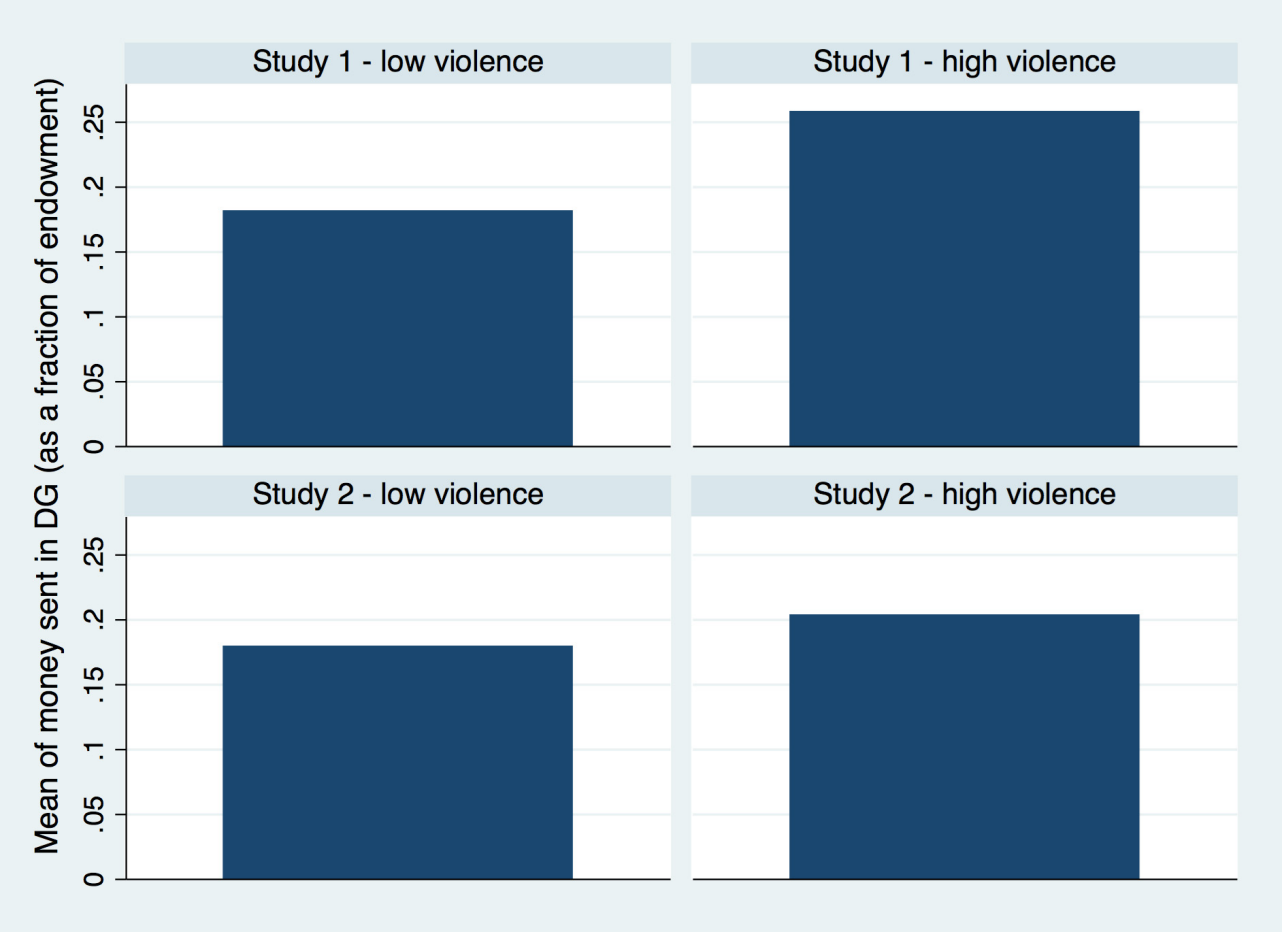
Average of money sent in the Dictator Game.

Altruism was measured using a one-shot dictator game. Money sent in the dictator game averaged 22% (See Fig 4) of the endowment, ranging from 0 to 83%, which is below the average of 27% found by [2]. But this is consistent with previous measures for the dictator Game in Colombia, which report a range between 19% and 44% [50]. The distribution of money sent was positively skewed (See S5 Fig).

To analyze the effect of subjective insecurity, we conducted a median regression, again using the same set of covariates. As for trust, we found that altruism was positively affected by subjective insecurity. The effect was significant at the 6% level. No other variable in the regression was observed to be related to altruism (See S1 Table and Fig 2).

### Discussion of Study 1

As mentioned above, in the behavioral literature trust, reciprocity and other-regarding preferences such as altruism are considered determinants of cooperative behavior. This could lead to the expectation that any relationship with subjective insecurity should be in the same direction for all prosocial behaviors. Although ours was a between-subjects design and hence we could not test individual consistency across preferences and behaviors, it is important to note that we are reporting different signs for each relationship. This divergence is not new in the literature exploring violence and prosocial behavior (see, for example, [10, 39]).

We speculate that subjective insecurity affected determinants of cooperation differently from other-regarding preferences and beliefs about others. In particular, subjective insecurity may be affecting the strategic component of cooperative behavior. Higher levels of subjective insecurity constrict cooperative choices, suggesting that fear and perception of threat impose cognitive and affective costs, thus driving individuals away from contributing to the public good. On the other hand, subjective insecurity is positively related to trust and altruism because other-regarding preferences are activated. This could be related in part to the nature of the games, which not only measure different aspects of behavior, but also create different dynamics. Cooperative behavior is measured using a simultaneous game in which the action of a group needs to be coordinated. On the other hand, trust (reciprocity) and dictator games are sequential activities in which a relationship, albeit an anonymous one, is established with a partner. Even after violence, this latter condition could facilitate the development of empathy toward others, or what has been called “selective trust” in the literature [11].

### Study 2

Summary statics of perceptions of insecurity by dimension and game are reported in Table 1. There were no significant differences in average aggregate subjective insecurity between the two studies (*M*_*sub*.*insec*.*study1*_ *= 1*.*67; M*_*sub*.*insec*.*study2*_ *= 1*.*69; p > 0*.*5*), and the distribution was also very similar (See S2 Fig). This was also true for participants in each game when considered separately. Notwithstanding this result, prosocial behavior varied between the two studies.

### Public Goods Game

In the public goods games, the mean contribution across all 15 rounds was significantly lower in Study 2 than in Study 1 (*M*_*cont*.*study1*_ *= 689*.*4; M*_*cont*.*study2*_ *= 537*.*9; p <* .*001*). Furthermore, in Study 2 there was evidence of decreasing contributions as rounds unfolded (see Fig 1). Though three years elapsed between the two studies, some strategic learning seems to have occurred, as is commonly reported in laboratory experiments with more trained participants. The distribution of contributions was again bimodal with two focal points at approximately one third and two thirds of the endowment (See S3 Fig).

In Study 2 we replicated the quantile regressions of the first study, using the same specification and covariates, but this time the two focal points were empirically located at quantiles 55 and 80 (See S1 Table). We also included three variables of victimization: being a homicide witness, being forcedly displaced and an aggregate index summarizing events that had occurred during the previous 12 months (e.g., robbery, aggressions, etc.). We included a dummy that controlled for participation in Study 1, in which 56% of the sample had also taken part, though not necessarily in the same game. We found that subjective insecurity was negatively associated with cooperation, but only for the 80^th^ quantile (See Fig 2). There was a significant and negative effect of having participated in Study 1. This might indicate strategic learning. The effects of membership of the FNC and of knowing people also taking part in the game were not observed in Study 2. As in Study 1, the effect of education was negative. Victimization variables, used only in Study 2, affected cooperation: Being a homicide witness increased cooperation in both quantiles and having been displaced reduced cooperation in the 80^th^ quantile. Regarding the index of aggregated victimization events, the higher the index the lower the cooperation in both quantiles.

### Trust Game

On average, participants in the sender role sent 36.6% of the endowment across the five rounds and receivers sent back an average of 52%, following multiplication by 3. No significant change in money sent was observed as the rounds unfolded (*F = 0*.*91*, *p = 0*.*47*), but the average was significantly lower than in Study 1 (*t = 9*.*43; p <* .*001*) (See Fig 3). The distribution was very similar to the earlier study (S4 Fig). Similarly, the difference in money returned was also significantly less (*t = 3*.*01; p < 0*.*01*). For money returned, no significant change was observed as the rounds unfolded (F = 0.85, p = 0.49) but the average was significantly lower than in Study 1 (t = 13.15; p < .001) (See Fig 3). The distribution was very similar to that of Study 1 (S4 Fig).

We conducted panel linear regressions with population-averaged effects, using the same specification as in Study 1, plus the three victimization variables and a dummy variable that were used to capture participation in Study 1. Again, we found that subjective insecurity was positively related to trust (i.e. money sent to player B) but the effect was weaker. (See S1 Table and Fig 2). The same effects of round and money returned from player B were observed. The only victimization variable that showed an effect was the aggregate index. As in Study 1, no effect of subjective insecurity was observed in reciprocity (See S1 Table and Fig 2).

### Dictator Game

Players sent on average 19% of the endowment (See Fig 4), ranging from 0 to 83%, and the distribution was similar to that of Study 1 (See S5 Fig); nor was the mean different from the one observed in that study (*t = 1*.*34; p = 0*.*18*). We replicated the same regression model used in the earlier study, with the addition of the victimization variables. However, we found no effect for subjective insecurity or victimization variables on money sent. In contrast to cooperation and trust, participation in Study 1 did not have a significant negative effect in the dictator game. Regression results are presented in S1 Table and Fig 2.

### Mediation Models

**Fig 5:**
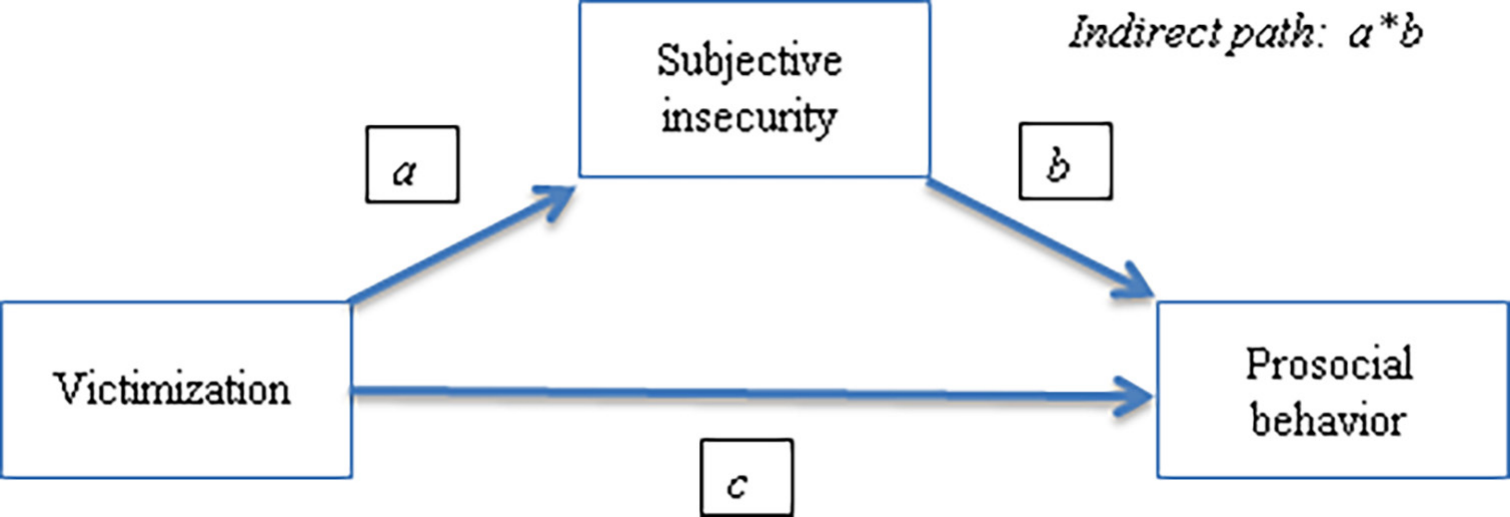
Path diagram of the direct and indirect (mediated) effects of victimization on subjective prosocial behavior.

In order to better capture the linkages between victimization, subjective insecurity and prosocial behavior, we tested mediation models where victimization anteceded subjective insecurity and subjective insecurity affected prosocial behavior. These models allowed testing of whether the effect of victimization on prosocial behavior was mainly direct and independent of individual heterogeneous responses to violence or, as hypothesized, the effect of victimization on cooperation was also indirect and depended on subjective experiences of violence and on the effect of victimhood on subjective insecurity. Fig 5 displays a path diagram that captures these relationships visually.

As explained previously, we tested three models, one for each type of victimization: events occurred in the previous 12 months, witnessing a homicide and being displaced. Since we had no results for altruism or reciprocity in Study 2 we were only able to test the mediation models for cooperation and trust.

To do so, we needed to estimate the different coefficients depicted in Fig 5. To obtain the *a* coefficients, using an OLS model we first regressed the subjective insecurity of each of the three types of victimization, with the same covariates used in Study 2 (This regression is reported in S2 Table). For coefficients *b* and *c*, we use the regressions already conducted for cooperation and trust to analyze the effect of subjective insecurity, controlling for victimization and covariates (regressions reported in S1 Table). The products of the coefficients of victimization on subjective insecurity (coefficients *a*) and subjective insecurity on cooperation and trust (coefficients *b*) were calculated, and these products were taken as the indirect effect. The Sobel test of mediation was performed to test for the significance of the indirect effects (a*b). For cooperation, we report the mediation models for this quantile only, since the effect of subjective insecurity was significant only for the 80^th^ quantile of the cooperation distribution.

For cooperation, the indirect (mediated) effect was significant and negative for general victimization (Coefficient a*b = -35.*09; z = - 2*.*37; p <* .*05*) and for witnessing a homicide (Coefficient a*b = -22.*85; z = -2*.*36; p <* .*05*). There was no significant effect for displacement. Interestingly, the direct effect (coefficient *c*) of general victimization on cooperation was also negative while the direct effect of witnessing a homicide was positive (see S1 Table). The direct effect of being displaced was not significant. Meanwhile, subjective insecurity decreased cooperation, in a finding that was consistent with previous analyses. Displacement did not affect either subjective insecurity or cooperation.

Pertaining to trust, there was no significant mediation for displacement or for witnessing homicide. A significant indirect (mediated) relation was observed for general victimization (Coefficient a*b = 55.*6; z = 1*.*69; p <* .*1*). This effect was positive, while the direct effect of general victimization on trust was negative (Coefficient a*b = -202.*4; z = -2*.*24; p <* .*05*). Furthermore, for trust, no direct effect either of displacement or of witnessing homicide was observed (see S1 Table).

These analyses also allowed us to assess the possible causes of subjective insecurity. As expected, the three types of victimization increased subjective insecurity significantly, as reported in the regression in S2 Table. Subjective insecurity was also increased by education and gender (females reporting higher subjective insecurity).

These results highlight the importance of heterogeneous responses to violence because, regardless of the type of victimization, as long as it increases subjective insecurity, the subsequent effect of subjective insecurity is negative on cooperation and positive on trust. Meanwhile, the direct effect of victimization on both cooperation and trust depends on the type of victimization. The explanation for these finding should be a matter of further research.

In summary, Study 2 confirms the results of Study 1 and suggests that subjective insecurity mediates the effects of exposure to violence on cooperation and trust. The effects on cooperation are stronger than on trust, and are observable for relatively high levels of cooperation. The effect on trust is positive only for the aggregate index of victimization.

### Discussion of Study 2

Controlling by victimization, the results for cooperative behavior for the 80th quantile were the same as for Study 1. This suggests that the negative effect of subjective insecurity is more salient for high levels of cooperative behavior. We also found a positive effect of subjective insecurity on trust, as in Study 1. However, the original finding for altruism was not replicated. Therefore, we cannot conclude that this is a stable finding. One possible explanation is that in Study 2 participants with higher subjective insecurity sent less in the dictator game.

In short, we report: i) a negative relationship between subjective insecurity and cooperation, and ii) a positive relation between subjective insecurity and trust. Our results are novel, because they help to understand the effects of violence on behavior, focusing on the subjective experience of such violence. The results are also novel from a methodological standpoint, because to our knowledge this is the first time that field experiments have been replicated within the same population.

Furthermore, by including victimization in a mediation model, we were able to evaluate both the direct effects of victimization, previously explored in the literature, and its indirect effects on prosocial behavior, via subjective insecurity. The nature of the indirect effects depends on the heterogeneous individual responses to violence, and our results highlight the relevance of such heterogeneity in understanding the relationship between violence and prosocial behavior. We found that the direct effect of victimization on cooperation depends on the type of victimization, while the indirect effects are consistently negative. Finally, for trust, the direct effect of aggregate victimization on trust is negative and the indirect effect is positive.

Interestingly, our results show that aggregate victimization and witnessing a homicide increase subjective insecurity but that displacement does not have the same effect. Therefore, even though displacement is a very difficult situation to live through, it may constitute a mechanism to mitigate insecurity by migrating to a different location.

## Conclusions

Cooperative behavior and trust are desirable for many reasons. They could be understood as proxies, or components, of social capital and, as such, antecedents of collective action (e.g. [6, 30, 31]). This research builds on the classical problems of social capital by centering the analysis on the subjective assessments of insecurity carried out by individuals. Consistent with the model of cognitive institutionalism proposed by Mantzavinos et al. [51], in which outcomes alter reality through a process of feedback occurring in the mind, we argue that individuals experiencing similar conditions of violence in the same environment may develop different perceptions of insecurity, which in turn affect prosocial behavior.

Our results contribute to the literature by discussing the interplay between exposure to violence and prosocial behaviors (see for example [9, 10, 11–14, 39]). By moving away from objective, aggregate and sometimes narrow and problematic measures of violence toward subjective, individual-level measures of insecurity that are wider in scope, we find a negative relationship between perceptions of insecurity and cooperative behavior and a positive relationship between subjective insecurity and trust. Cooperation and trust were experimentally measured in two different moments, separated by three years (Study 1 and Study 2). These results might reveal a behavioral pattern whereby the subjective experience of insecurity increases prosocial preferences (expressed in more trust) but triggers strategic behavior that overrides personal preferences (expressed in less cooperation). An important caveat is that such difference could be related in part to the nature of the games used to study individual decisions, which measure different aspects of behavior, and also create different dynamics.

We went more deeply into the nature of these relationships by exploring the antecedents of subjective insecurity in relation to victimization experienced at the individual level. We found an expected positive relationship between different indicators of victimization and subjective insecurity. However, each indicator of victimization displayed a unique relationship with cooperative behavior and trust. This motivated the use of mediation models through which the connection from victimization to cooperation and trust were allowed at least two paths, one of them direct, and depending on the type of victimization, and the other indirect, depending on the heterogeneous individual experience. The models result in different levels of subjective insecurity and capture the complex dynamics that result from the interplay of each type of victimization and the heterogeneous response to it expressed in subjective insecurity. Though further research is needed, the work we undertook suggests that the reported increase of trust and the decrease of cooperation resulting from subjective insecurity is attributable to the competing forces of individual preferences and contextual influences.

Our results show that, as a necessary component of human development, it would be difficult to improve cooperation under conditions of increased subjective insecurity, although interpersonal trust might improve. People find it difficult to shake off a feeling of insecurity and to cooperate in pursuit of collective objectives. Instead, as feelings of insecurity are based on “*appraisal of threat and coping capability”*, people who feel insecure may tend to reduce their contributions to collective efforts because they might feel their vulnerability is increasing [15]. Inversely, when subjective insecurity is high, people are more likely to trust their partners in the search for mutual benefits.

This research has shown subjective insecurity to be a powerful tool to improve understanding of what constricts people’s choices and community development in a context of violence. In this sense, our results suggest a new puzzle that requires further research. While individual-centered measures such as emotions and beliefs negatively affect cooperation, some measures of victimization increase it. The opposite occurs for trust. New research designs are needed to explore these relationships. Field and laboratory studies could be designed to reveal these mechanisms and their interactions, controlling by exposure and time elapsed subsequent to the occurrence of violent events.

## Methods

In 2011 and 2014, we conducted two studies with farmers in rural areas of the region of Antioquia, Colombia. This is one of the main coffee-producing areas of the country, where different kinds and intensities of violence and conflict have been present for considerable periods of time. We selected four municipalities in the region that had experienced different average levels of objective violence over the previous 10 years in order to increase the expected variance of subjective insecurity. We used official data compiled by the Presidential Observatory for Human Rights on homicide rates, number of kidnappings, number of displaced individuals (expulsion) and number of armed clashes (such as attacks on police and army facilities, ambushes, and acts of harassment) to construct a municipal violence index for the period 1998–2010. This index was calculated by averaging the normalized indicators for each year and using the “peak-end rule” [52, 53], a psychological heuristic according to which the subjective judgment of an experience that occurs over time depends mainly on the maximum and final levels of that experience. Thus, for the “peak-end rule,” we used the average of these two values to capture the salience of extreme and recent events within a long- but low-intensity conflict, such as the one affecting Colombia as a whole and Antioquia in particular [54, 55].

We calculated indices for all the municipalities in Antioquia (n = 125) and selected two municipalities, Sopetrán and San Jerónimo, with relatively low violence indices (LV) and two municipalities (Abejorral and Betulia) where these indices were relatively high (HV).

The classification of these four municipalities was consistent when using the average violence and the peak-end rule. For Betulia the average based index (ABI) was 1.98 and the peak-end rule based index (PER) 4.02. For Abejorral, the ABI was 1.15 and the PER 1.65; for San Jerónimo, the ABI was 0.28 and the PER 0.17; for Sopetrán, the ABI was 0.21 and the PER 0.48. The municipalities were first selected taking into consideration their similar characteristics of population size, distribution of ethnic groups, average farm size, income, and municipal expenditure. From a subgroup of relatively homogenous municipalities in those criteria, we selected the four that offer the highest difference in objective violence. Therefore, in the final selection, HV municipalities displayed an index above the 60th centile and LV municipalities below the 20th centile for both methods. We conducted both studies in the same municipalities.

In each municipality a total of 240 participants were recruited, with 80 participants randomly assigned to each game. In each study we used a between-subjects design with a standard repeated public goods game with a partners design for 15 rounds (e.g. [44]) to measure cooperation; a standard repeated investment game with a partners design for 5 rounds [56] to measure trust (and reciprocity), and a one-shot dictator game to measure altruism (e.g. [57]). All the games are versatile and well-established economic experiments that have been used in many contexts and for varied purposes, including to measure social capital (See, for example, [1, 2, 30, 31, 44, 58]).

In each study, participants were able to take part in one experiment. In each game, after instructions were read, participants participated in a quiz to guarantee understanding. Research assistants clarified any questions in private when confusion was identified by the quiz. Subsequently, three practice rounds were played before implementation of the public goods game for payment, and two before the trust game. The same person read the instructions in both studies.

We conducted four to five half-day sessions in each municipality. In the first two days of field work we conducted four sessions of the public goods game, with 20 participants each. In the following days we implemented four sessions of the trust game with 20 participants each, and two sessions of the dictator game with 40 participants each. Participants did not know in advance which game they would be playing. For both studies, in each municipality and in each session we followed exactly the same procedure, including the order in which the games were implemented. After the experiment we conducted a survey to collect data on subjective insecurity along with other relevant socioeconomic information. This information was collected anonymously in both studies, and participants completed written consent forms. The study was approved by the Research Committee of the School of Management at Universidad de Los Andes.

One year after the experimental sessions conducted for Study 1 we conducted community workshops with leaders and participants in the experiments to gather information about the contexts of the municipalities and to gain an understanding of the differences in participants’ perceptions of insecurity and the way violent events in the past might have shaped them. A summary of this information is reported in S1 Text together with the experimental instructions and questionnaires used in the study (S2–S8 Texts).

### Study 1

#### Participants

We recruited 80 farmers for each game, drawn from different rural districts in each of the four municipalities, for a total of 960 participants. However, here we report data for only 796 participants: we do not analyze data for participants B in the dictator game (160 participants) who did not make any decision, nor for four participants who did not show up for one of the trust sessions. The FNC helped with recruitment, spreading the word through public announcements and local community leaders. Recruitment targeted adult peasant farmers in the rural districts of the four municipalities. We deliberately sought a balanced representation of coffee and non-coffee producers in order to reduce biases resulting from affiliation to the FNC. Our sample cannot be considered representative of the adult population in these municipalities. We did not perform a random or quota sampling in the rural districts. Instead we focused on those rural districts where the FNC had the infrastructure to help with the recruitment and we did not include the urban population. In some rural districts the entire adult population was covered. Table 2 presents descriptive statistics for participants’ age, income, gender, level of education, FNC affiliation, and the average number of people known to them, by session, for each municipality and for each game. We controlled for these variables in our estimations (See S3 Table for details on these descriptive statistics).

**Table 2:** Sample average characteristics by game and by municipality: Study 1.

	N	% Men	Age	Years of education	Monthly Income (USD)	FNC Affiliation	% Known people per session
**By Game**							
Public goods	320	0.44	42.76	5.20	125.72	0.52	0.70
Trust	158	0.59	42.68	4.76	144.34	0.58	0.66
Reciprocity	158	0.60	43.17	4.77	159.19	0.50	0.71
Dictator	160	0.63	42.91	5.03	128.45	0.54	0.42
**By Municipality**							
**HV-** Abejorral	196	0.59	47.80	4.87	117.52	0.69	0.70
**HV-** Betulia	200	0.50	39.45	4.95	193.71	0.55	0.43
**LV**–Sopetrán	200	0.52	45.02	5.04	126.59	0.52	0.73
**LV-** S.Jerónimo	200	0.54	39.44	5.09	107.50	0.37	0.69
**Overall**	**796**	**0.54**	**42.89**	**4.99**	**136.40**	**0.53**	**0.64**

Exchange Rate for December 2015: 3,149.47 COP = 1 US$

#### Procedure

Data collection took place during November and December of 2011. For the public goods game, we followed the procedure reported by [44]. Participants were randomly assigned to groups of five individuals. Each person received an endowment of 1,500 COP from the experimenter in each round. Then, within each group, participants were asked to contribute to a community project. Individual contributions were private and anonymous to reduce bias of strategic behavior and only total contributions were announced to the group. The sum of individual contributions to the project was multiplied by two and the total amount was then distributed in equal parts to the five members of the group (MPCR = 0.4). Individual earnings corresponded to the endowment minus the contribution plus the amount returned to each participant after the “project” took place. The game was played three times for practice to facilitate understanding of the procedure, and then 15 times for money. Communication was not allowed. Participants were fully informed of the whole procedure and were provided with materials to keep track of their individual decisions and earnings. Total gains were the lump sum of the gains from each of the game’s 15 rounds. Four groups of five participated at the same time in each session. Participants knew who was in their group but did know their individuals contributions. We obtained a panel of 4,800 individual contributions.

We conducted a trust game following the original design proposed by [56]. Participants were randomly assigned either to role A or role B and played for 5 rounds with the same unknown partner. Participants A and B both received an initial endowment of 3,000 COP in each round. Participant A first decided how much to send to participant B. Each amount sent by participant A was tripled by the experimenters. Next, participant B decided whether to give back any money to participant A. We allowed participants B to include their endowment in the quantity of money returned. Earnings for A in each round corresponded to the initial endowment minus the amount sent plus what participant B decided to give back. Earnings for B were calculated as the endowment plus the amount sent by A tripled, minus the amount s/he decided to give back to A. Total gains were the sum of gains in each round. Communication was not allowed and all decisions were made anonymously. A final 6th round with a new anonymous partner was also implemented, but this round was excluded from this analysis.

The amount sent by Participants A was a measure of trust, while the amount returned by participants B captured individual reciprocity (See, for example, the discussion in [36]). We obtained a panel of 1,896 observations for each trust and reciprocity measure.

Finally we conducted a one-shot dictator game to measure individuals’ preferences for altruism. Following the standard procedure, participants were randomly assigned to role A or role B. Participants A played as the dictator and decided how much to send, from their initial endowment of 60,000 COP, to an anonymous participant B. Participant B’s earnings were the amount sent by A, while Participant A’s earnings were the initial endowment minus the amount sent to B. We obtained 160 individual observations of altruism for each study.

Total payments were calculated (sum of gains in each round) and participants were paid and dismissed. Each person was only allowed to participate in one game and earnings covered participants´ opportunity costs. The average earnings for Study 1 (in US dollars) were $9.90 for the public goods game; $6.11 for senders and $9.48 for recipients in the trust game; and $14.80 for senders in the dictator game. Calculated using the exchange rate for December 2015, one US dollar was equivalent to approximately 3,149 COP.

#### Subjective insecurity

To measure subjective insecurity we developed a questionnaire capturing affective and cognitive aspects of the construct. We adapted some questions from [24, 25, 18] and from the National Survey of Victimization [59] for a rural context. Questions were designed to capture variations in the intensity of perceptions independently of their causes (e.g. armed conflict, domestic violence or crime). That is, we did not inquire into the source of the perception of insecurity because we were interested in an overall sense of insecurity. Understanding the sources of vulnerability is outside the scope of this paper. See, for example [60] for suggestions on human security operationalization and [15] for the sources of feeling insecurity as a psychological process.

Our basic questionnaire was composed of eight questions designed to capture feelings of fear related to different general aspects of threats and vulnerabilities (affective side), as well as subjective estimates of the possibility of experiencing violence (cognitive side).

Table 3 contains the eight basic questions for personal subjective insecurity. (Note that Question 1 was reversed, in order to calculate the aggregate insecurity index). Besides personal insecurity, we also wanted to capture variations of subjective insecurity as a function of social distance. Social distance is related to other psychological distance judgments such as the hypotheticality of events [61]. Hence, underlying estimates (i.e. subjective probabilities) of being targeted by violence may vary as a function of social distance, affecting subjective insecurity. In addition, estimations of security anchored at different egocentric distances may also be affected by other well-known subjective biases and judgment miscalibrations such as over-confidence [62], specifically in terms of excessive precision in beliefs about insecurity [63]; illusory correlations between the occurrence of violent acts and social contexts [64]; and the illusion of control [65], as many people may believe that they have a certain control over threats within their own lives and families as opposed to the communities to which they belong.

**Table 3:** Personal subjective insecurity questions.

	1. Totally disagree	2. Partially disagree	3. Partially agree	4. Totally agree
1. I feel safe when going out at night				
2. I feel I could face threats to my life				
3. I fear for my life				
4. I feel I face risks when participating in social, economic and political meetings				
5. I fear being robbed by day				
6. I fear being robbed by night				
7. I fear personal aggressions by day				
8. I fear personal aggressions by night				

Accordingly, the questions were framed by three social-distance-related dimensions of insecurity from an egocentric perspective (i.e., distance from the self) [66]. Thus we asked the same eight questions at the personal, family (household) and community levels. For example “*I feel I could face threats to my life*” was framed as “*I feel that the members of my household could be threatened*” for the family level, and “*I feel that my neighbors could be threatened*” for the community level. In order to capture community-specific items of insecurity we replaced two of the base questions (“robbed by day” and “aggressions by day”) with concerns on “children playing safely” and the presence of a “protective authority”. The full set of questions is available in S3 Text.

The survey used a four-point bipolar Likert scale to capture variations in the intensity of perceptions of insecurity. The scale is symmetric, ranging from 1 (totally disagree) to 4 (totally agree), and does not have a central point, in order to force respondents to go in one direction or the other. Thus, we avoided the risk of an overestimated middle focal point in responses.

In addition to questions on the perceptions of insecurity, the survey included a wide array of questions intended to collect socio-demographic information on gender, income, education level, and economic activities (See S3 Text).

### Study 2

#### Participants

Three years after the first measure, we returned to the same villages and repeated the initial design (public goods game, trust game, dictator game and measure of subjective insecurity), adding measures of different indicators of victimization. Replicating the design of Study 1, we recruited 80 farmers for each game, from different rural districts in each of the four municipalities, for a total of 960 participants. We report 800 participants because we did not analyse data for participants B in the dictator game (160 participants).

#### Additional measures

To measure victimization we asked participants to self-report whether they had been victims of different types of violent acts according to the time span in which they had occurred: whether the incident had happened during the last 12 months (e.g., robbery), or during the course of their lives (e.g. witnessing a homicide or a forced displacement). See Table 4 for the full set of questions.

**Table 4:** Victimization questions.

*In the last 12 months, you have been a victim of:*	Yes	No
**a.** Non-armed robbery		
**b.** Armed robbery		
**c.** Assault or sexual violence		
**d.** Kidnapping		
**e.** Street fight		
**f.** Domestic violence		
**g.** Damage to your property, crops or animals		
**h.** Extortion		
**i.** Verbal aggression		
**j.** Harassment		
**k.** Persecution		
**l.** Psychological abuse		
**m.** Death threats		
**n.** Other		
*During the course of your life you have been a victim of:*		
**o.** Forced displacement		
**p.** Rape		
***q*.** *During the course of your life have you witnessed homicide(s)?*		

We acknowledge that self-reports of victimization may underestimate exposure to violence, but since we were not attempting to estimate the actual level of violence but the relationship between victimization and subjective insecurity, a certain amount of misreporting does not affect our conclusions.

As in the first study, the FNC and local community leaders helped with recruitment. This was not intended to be a panel study at the individual level, since participation was anonymous both in Study 1 and Study 2. However, we recruited subjects in the same rural districts and targeted the same type of individuals. As a result, we obtained a sample that was demographically very similar to that of Study 1 (see Table 5 and S3 Table). The average game earnings in Study 2 (in US dollars) were $10.30 in the public goods game; $7.30 for senders and $9.59 for recipients in the trust game; and $15.40 for senders in the dictator game.

**Table 5:** Sample average characteristics by game and by municipality: Study 2.

	N	%Men	Age	Years of education	Monthly income (USD)	FNC Affiliation	% Known people per session	% Participated 1^st^ Study
**By Game**								
Public goods	320	0.55	41.95	5.53	146.37	0.65	0.71	0.56
Trust	160	0.38	41.67	6.02	135.85	0.67	0.78	0.50
Reciprocity	160	0.41	41.83	5.63	160.19	0.63	0.78	0.51
Dictator	160	0.43	44.81	5.43	122.10	0.62	0.63	0.47
**By Municipality**								
**HV-** Abejorral	200	0.43	45.30	5.95	147.92	0.69	0.73	0.51
**HV-** Betulia	200	0.46	38.10	5.96	171.80	0.72	0.66	0.28
**LV-** Sopetrán	200	0.44	46.05	5.24	119.01	0.68	0.82	0.57
**LV-** S. Jerónimo	200	0.53	41.49	5.21	136.70	0.50	0.67	0.73
**Overall**	**800**	**0.46**	**42.73**	**5.59**	**143.92**	**0.65**	**0.72**	**0.52**

Exchange Rate for December 2015: 3,149.47 COP = 1 US$

## Supporting Information

S1 Fig Screeplot of insecurity. (TIF)

S2 Fig Distribution of insecurity. (TIF)

S3 Fig Distribution of Public Goods Game. (TIF)

S4 Fig Distribution of Trust Game and Reciprocity. (TIF)

S5 Fig Distribution of Dictator Game. (TIF)

S1 File Data. (CSV)

S1 Table Regression models for all prosocial behaviors. (DOCX)

S2 Table Median regression of the effect of victimization on subjective insecurity. (DOCX)

S3 Table Details about the sample. (DOCX)

S1 Text Workshops. (DOCX)

S2 Text Informed consent. (DOCX)

S3 Text Survey. (DOCX)

S4 Text Instruction Public Goods. (DOCX)

S5 Text Instructions Trust player A. (DOCX)

S6 Text Instructions Trust player B. (DOCX)

S7 Text Instructions Dictator player A. (DOCX)

S8 Text Instructions Dictator player B. (DOCX)
