# Correction: Prosocial Behavior and Subjective Insecurity in Violent Contexts: Field Experiments

**DOI:** 10.1371/journal.pone.0198020

**Published:** 2018-05-24

**Authors:** María Alejandra Vélez, Carlos Andres Trujillo, Lina Moros, Clemente Forero

There is an error in the ultimate sentence of the Abstract. The correct sentence is: Different indicators of victimization are positively correlated with subjective insecurity and an aggregate index of victimization has a negative direct effect on cooperation and trust.

There are errors in the fourth, fifth, and sixth paragraph under the Mediation Models heading of the Results section. The corrected paragraphs are: For cooperation, the indirect (mediated) effect was significant and negative for general victimization (Coefficient a*b = -49.*57; z = -3*.*21; p = 0*.*001*) and for witnessing a homicide (Coefficient a*b = -11.*52; z = -1*.*90; p =* .*056*). The direct effects (coefficient *c*) of general victimization and of forced displacement on cooperation were also negative while the direct effect of witnessing a homicide was positive. For trust, a significant positive indirect (mediated) relation was observed for general victimization (Coefficient a*b = 56.*08; z = 1*.*67; p =* .*09*). The direct effect of general victimization on trust was negative (*Coefficient c = -202*.*4; z = -2*.*24; p <* .*05*). Two types of victimization increased subjective insecurity significantly (general victimization and witnessing homicide), as reported in the regression in S2 Table. Subjective insecurity was also increased by education and gender (females reporting higher subjective insecurity).

In S1 Table, the coefficients of two control variables were switched and decimals were misplaced. Please view the correct S1 Table below.

## Supporting information

S1 Table Regression models for all prosocial behaviors. (DOCX)
