# Supplementary figures and images for: Prosocial Behavior and Subjective Insecurity in Violent Contexts: Field Experiments
Source: PLoS One. 2016 Jul 29;11(7):e0158878. doi: 10.1371/journal.pone.0158878 (PMC4966936; doi:10.1371/journal.pone.0158878)

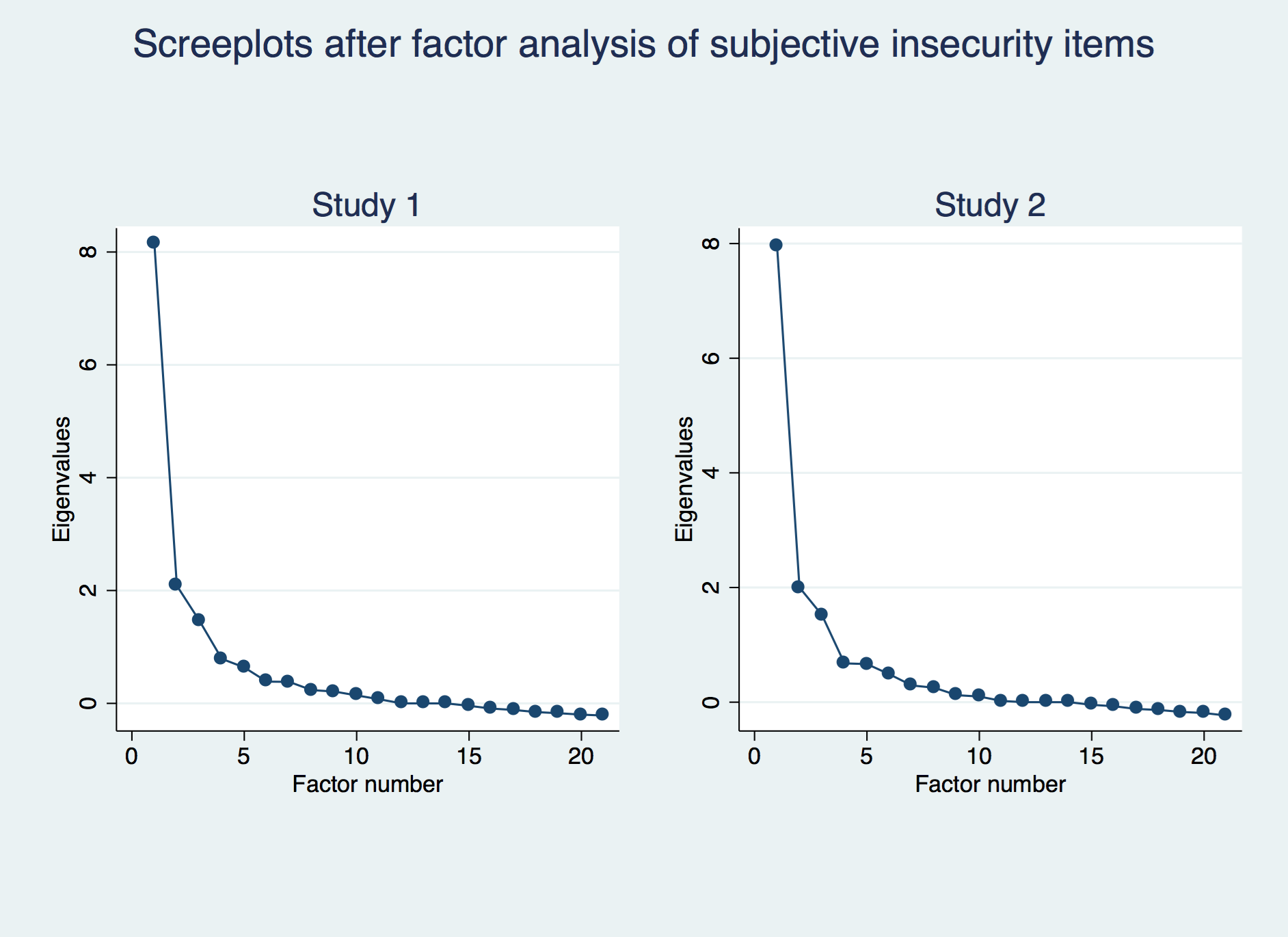

Supplement: S1 Fig — (TIF) [file pone.0158878.s001.tif]

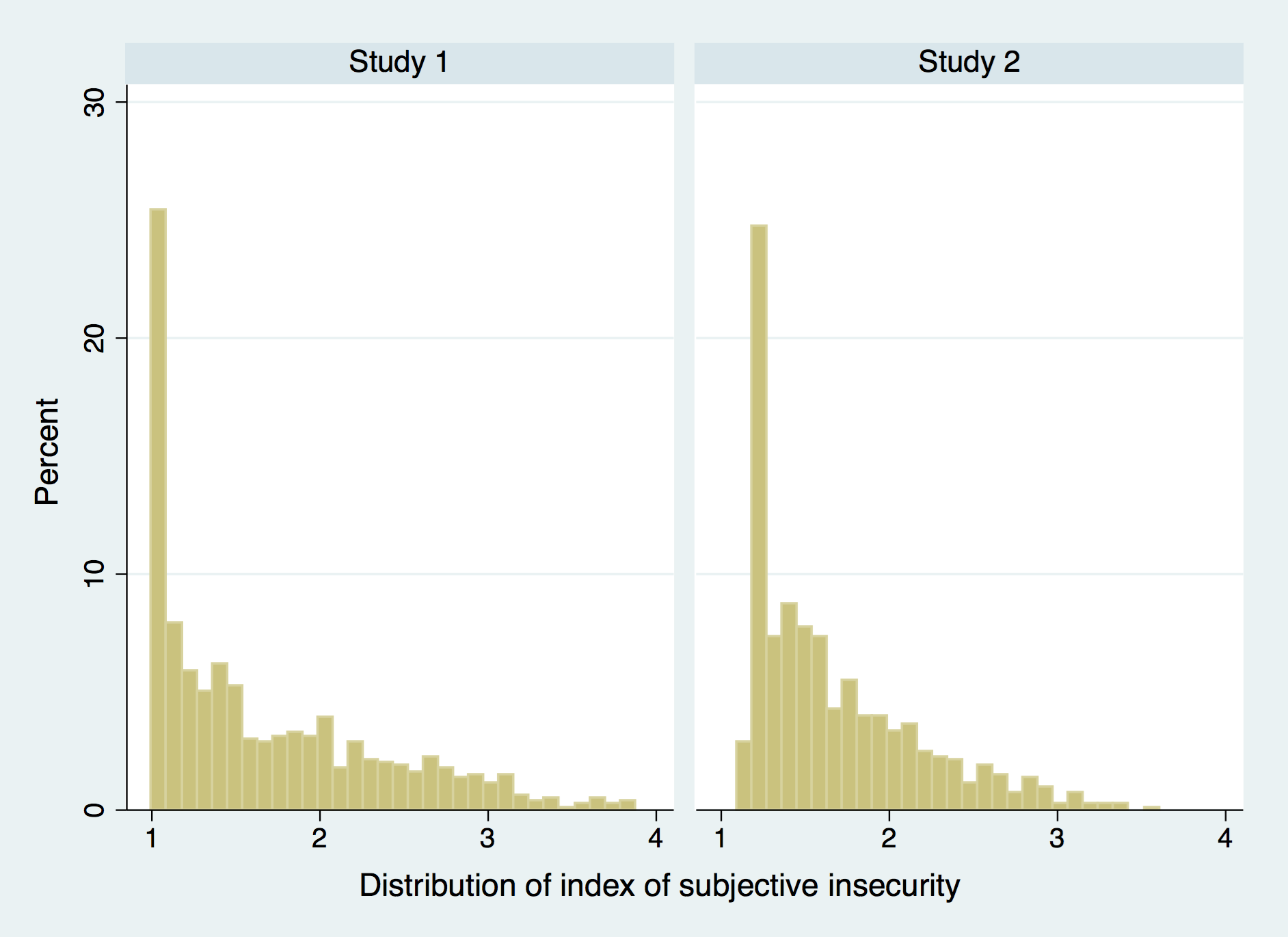

Supplement: S2 Fig — (TIF) [file pone.0158878.s002.tif]

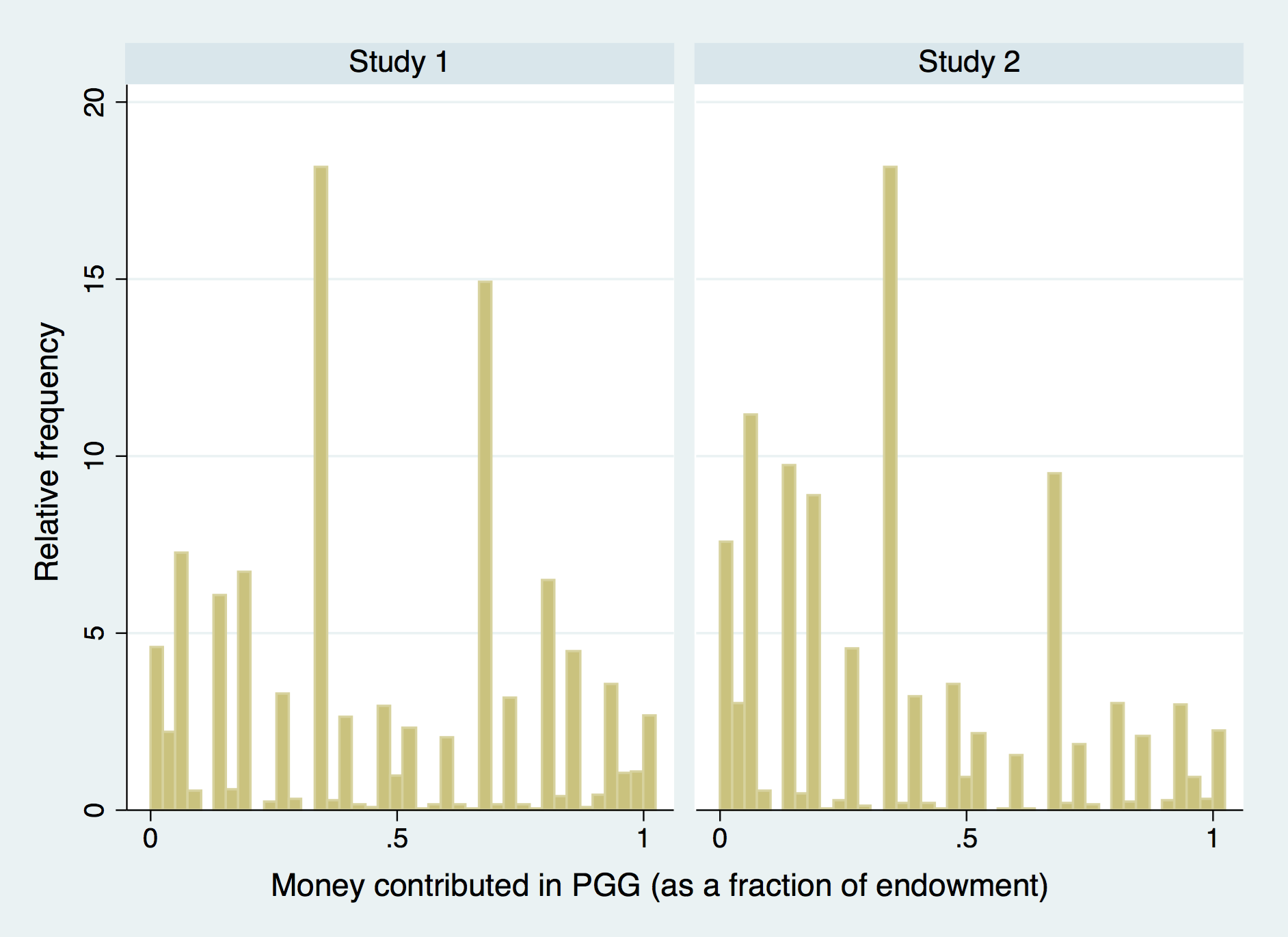

Supplement: S3 Fig — (TIF) [file pone.0158878.s003.tif]

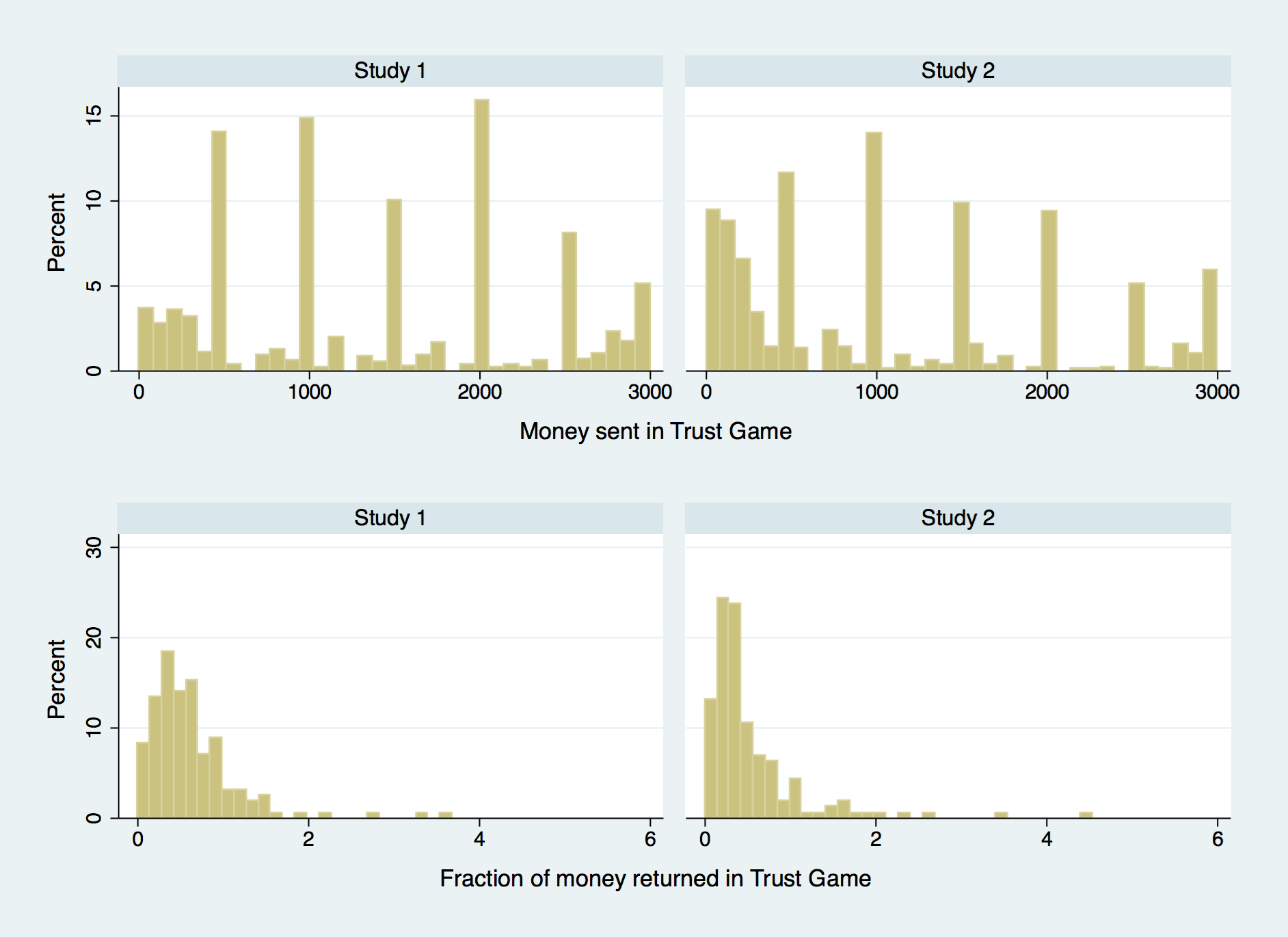

Supplement: S4 Fig — (TIF) [file pone.0158878.s004.tif]

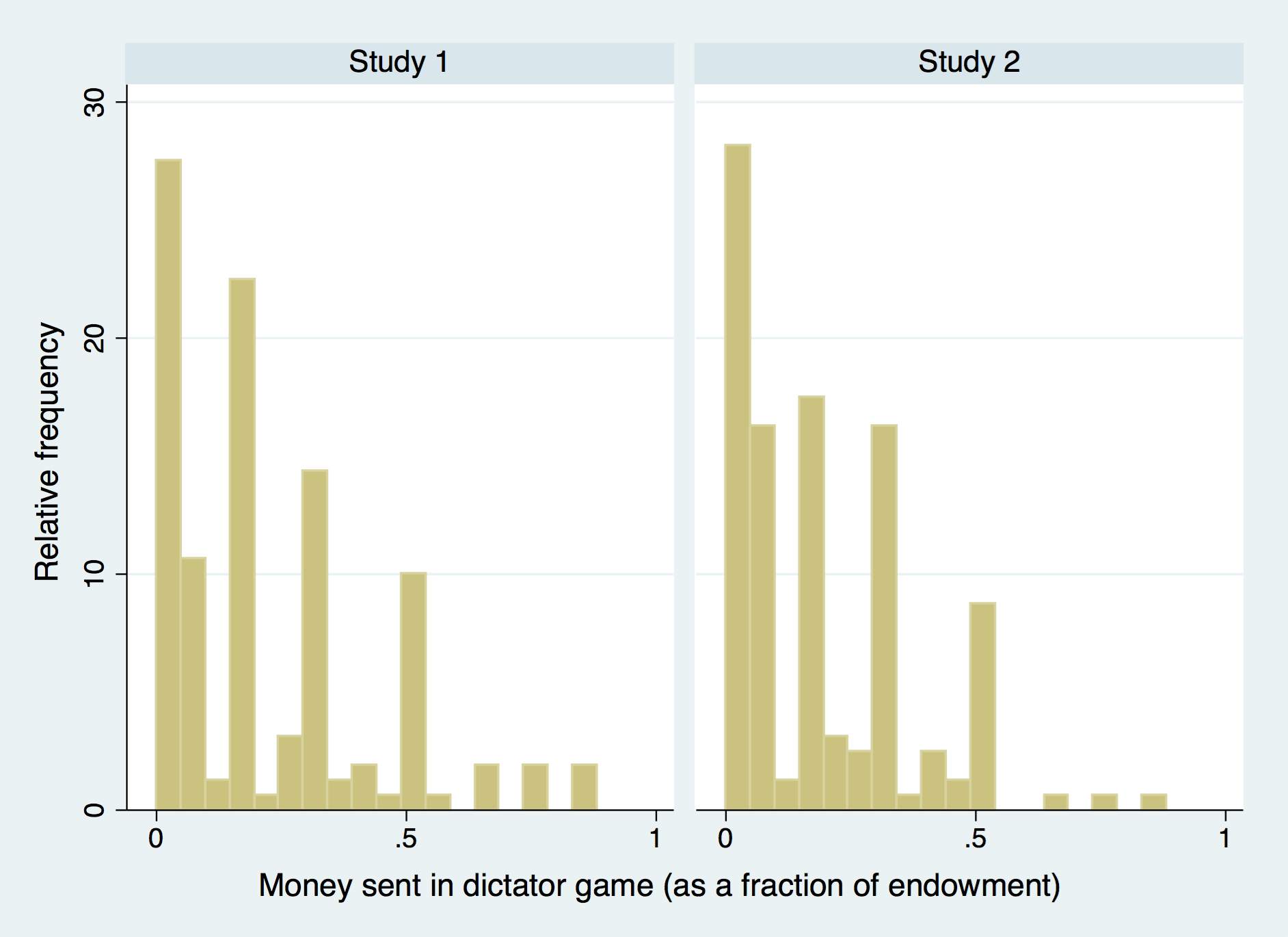

Supplement: S5 Fig — (TIF) [file pone.0158878.s005.tif]
