# Supplementary material for: Prosocial Behavior and Subjective Insecurity in Violent Contexts: Field Experiments
Source: PLoS One. 2016 Jul 29;11(7):e0158878. doi: 10.1371/journal.pone.0158878 (PMC4966936; doi:10.1371/journal.pone.0158878)
Supplement: S1 Table — (DOCX) [file pone.0158878.s007.docx]

**S4 Table. Regression models for all prosocial behaviors**

|  | **Cooperation^1^** | | | | **Trust^2^** | | **Altruism^3^** | | **Reciprocity^2^** | |
| --- | --- | --- | --- | --- | --- | --- | --- | --- | --- | --- |
| **Variables** | **1st Study** | | **2nd Study** | | **1st Study** | **2nd Study** | **1st Study** | **2nd Study** | **1^st^ study** | **2^nd^ study** |
|  | Q45 | Q75 | Q55 | Q80 |  |  |  |  |  |  |
| **Subjective insecurity** | -76.98*** | -58.08** | 14.49 | -111.90*** | 159.20*** | 126.60* | 4,61* | 2.33 | 0.02 | 0.06 |
|  | (21.78) | (23.93) | (27.73) | (35.23) | (61.55) | (74.58) | (2.40) | (2.58) | (0.07) | (0.93) |
| **Victimization** |  |  | -77.80*** | -94.06** |  | 54.16 |  | -3.71 |  | -0.00 |
|  |  |  | (30.16) | (46.03) |  | (163.70) |  | (4.11) |  | (0.10) |
| **Homicide Witness (dummy)** |  |  | 93.25*** | 123.70*** |  | -110.30 |  | -1.19 |  | -0.14 |
|  |  |  | (26.39) | (36.76) |  | (85.82) |  | (2.75) |  | (0.11) |
| **Forced displacement (dummy)** |  |  | 8.75 | -103.00** |  | -202.50** |  | -4.19 |  | 0.40 |
|  |  |  | (27.54) | (45.19) |  | (90.56) |  | (3.02) |  | (0.25) |
| **Gender (male=1)** | 27.35 | 134.1*** | -34.50 | -4.122 | 7.534 | 51.69 | 682.50 | 2.02 | 0.04 | 0.30*** |
|  | (28.43) | (27.77) | (21.38) | (27.96) | (80.39) | (75.26) | (3.21) | (2.26) | (0.08) | (0.09) |
| **Age** | 1.477* | -1.208 | 5.279*** | 5.967*** | 3.259 | 4.76* | -22.88 | 122.20 | 0.00 | 0.00 |
|  | (0.888) | (1.179) | (0.750) | (1.439) | (3.213) | (2.86) | (117.00) | (82.00) | (0.00) | (0.00) |
| **Education (years)** | -15.70*** | -9.053* | -13.59*** | 4.719 | -10.15 | 30.33** | -336.50 | 606.70 | 0.02* | 0.00 |
|  | (4.575) | (5.287) | (2.620) | (4.895) | (13.51) | (11.94) | (541.00) | (375.00) | (0.01) | (0.01) |
| **Monthly income** | 0.00 | 0.00** | 0.00* | -0.00 | 0.00 | 0.00 | 0.01 | -0.00 | -0.00 | 0.00 |
|  | (0.00) | (0.00) | (0.00) | (0.00) | (0.00) | (0.00) | (0.01) | (0.00) | (0.00) | (0.00) |
| **Coffee producer (dummy)** | 59.01** | 86.32*** | -0.30 | -34.47 | -86.81 | 33.42 | -893.10 | 499.20 | -0.07 | 0.11 |
|  | (29.09) | (25.31) | (22.99) | (36.93) | (86.38) | (88.49) | (3.49) | (2.66) | (0.08) | (0.10) |
| **Known people in the game** | -7.90*** | -11.25*** | -2.85* | -1.44 | -13.97** | 2.57 | 275.10 | -18.77 | -0.00 | 0.00 |
|  | (2.28) | (2.53) | (1.57) | (2.56) | (6.57) | (8.27) | (180.00) | (116.00) | (0.00) | (0.00) |
| **Round** | 0 | 0 | -2.202 | 0 | -39.29*** | -33.56*** |  |  | -0.00 | -0.02* |
|  | (0.53) | (0.00) | (1.99) | -1.44 | (9.77) | (8.62) |  |  | (0.01) | (0.01) |
| **Pariticipated in 1^st^ Study** |  |  | -185.20*** | -180.70*** |  | -219.30*** |  | 3,37 |  | 0.04 |
|  |  |  | (20.80) | (29.76) |  | (76.94) |  | (23.60) |  | (0.09) |
| **Returned money (lagged)** |  |  |  |  | 0.04*** | 0.12*** |  |  |  |  |
|  |  |  |  |  | (0.01) | (0.01) |  |  |  |  |
| **Money sent by A** |  |  |  |  |  |  |  |  | -0.00*** | -0.00*** |
|  |  |  |  |  |  |  |  |  | (0.00) | (0.00) |
| **Constant** | 125.70 | 372.40*** | 587.80*** | 910.40*** | 1.26*** | 20.50 | -1.56*** | -13.88*** | 0.54 | 0.33 |
|  | (88.38) | (82.18) | (148.20) | (177.80) | (291.40) | (259.30) | (0.00) | (0.00) | (0.36) | (0.39) |
| **N** | 4800 | 4800 | 4800 | 4800 | 1575 | 1590 | 159 | 158 | 770 | 770 |
| **Wald chi^2^** |  |  |  |  | 169.40*** | 366.26*** |  |  | 195.63*** | 119.82*** |
| **Pseudo R^2^** | 0.09 | 0.10 | 0.09 | 0.12 |  |  | 0.22 | 0.37 |  |  |

1. Contribution (cooperation) in the PGG game was analyzed using simultaneous quantile regressions with robust clustered errors.
2. Money sent (trust) and money returned (reciprocity) in the Trust game were analyzed using linear panel data regressions with population averaged effects
3. Money sent (altruism) in the Dictator game was analyzed using median regressions.

In all models, dummy variables for rural districts were included to control for fixed effects, but not reported here. Standard errors in parentheses.

**** p<0.01, ** p<0.05, * p<0.1*
