# Supplementary material for: Prosocial Behavior and Subjective Insecurity in Violent Contexts: Field Experiments
Source: PLoS One. 2016 Jul 29;11(7):e0158878. doi: 10.1371/journal.pone.0158878 (PMC4966936; doi:10.1371/journal.pone.0158878)
Supplement: S2 Table — (DOCX) [file pone.0158878.s008.docx]

**S7 Table. Median regression of the effect of victimization on subjective insecurity**

| **Variables** | **Overall Insecurity Index** |
| --- | --- |
| Victimization | 0.443*** |
|  | (0.05) |
| Forced displacement (dummy) | 0.017 |
|  | (0.07) |
| Homicide witness (dummy) | 0.102** |
|  | (0.05) |
| Gender (man=1) | -0.103*** |
|  | (0.04) |
| Age | 0.000765 |
|  | (0.001) |
| Education (years) | 0.0170*** |
|  | (0.001) |
| Monthly income | 0 |
|  | (0) |
| Coffee producer (dummy) | 0.0457 |
|  | (0.04) |
| Known participants in the game | 0.00206 |
|  | (0.002) |
| Pariticipated in 1^st^ Study | 0.0497 |
|  | (0.04) |
| Constant | 1.110*** |
|  | (0.51) |
| N | 797 |

Standard errors in parentheses. Dummy variables for rural districts included but not reported in the table.

**** p<0.01, ** p<0.05, * p<0.1*
