# Supplementary material for: Prosocial Behavior and Subjective Insecurity in Violent Contexts: Field Experiments
Source: PLoS One. 2016 Jul 29;11(7):e0158878. doi: 10.1371/journal.pone.0158878 (PMC4966936; doi:10.1371/journal.pone.0158878)
Supplement: S3 Table — (DOCX) [file pone.0158878.s009.docx]

**S 16 Table. Details about the sample**

|  | | Min | Max | Mean | Std. Dev | Correlations | |
| --- | --- | --- | --- | --- | --- | --- | --- |
|  |  |  |  |  |  | Education | Income |
| Study 1 | Age (Years) | 18 | 86 | 42.8 | 15 | -0.44*** | -0.09*** |
|  | Education (Years) | 0 | 17 | 5.1 | 3.6 |  | 0.26*** |
|  | Monthly Income (USD) | 0 | 1904.7 | 132.8 | 145.3 |  |  |
|  |  |  |  |  |  |  |  |
| Study 2 | Age (years) | 18 | 88 | 42.1 | 15 | -0.51*** | -0.10*** |
|  | Education (Years) | 0 | 17 | 5.6 | 3.6 |  | 0.14*** |
|  | Monthly Income (USD) | 0 | 1587.3 | 147.1 | 132.5 |  |  |
