# Supplementary material for: Prosocial Behavior and Subjective Insecurity in Violent Contexts: Field Experiments
Source: PLoS One. 2016 Jul 29;11(7):e0158878. doi: 10.1371/journal.pone.0158878 (PMC4966936; doi:10.1371/journal.pone.0158878)
Supplement: S1 Text — (DOCX) [file pone.0158878.s010.docx]

## S8 Summary Workshops

As mentioned above, we conducted community workshops with leaders and participants of the experiments to gather information about the context of these municipalities, and to gain an understanding of the differences in their perceptions of insecurity and the way violent events in the past might have shaped them. Each workshop consisted of three activities. First, a timeline was constructed to identify the most important stages in the community history. Second, a historical graph was plotted in order to recognize the existence and development of social and economic organizations. And third, a rules matrix was designed to establish changes in social norms over time. The methodology of these workshops was based on Arjona (2008, 2010).

### Abejorral

Workshops were carried out in three rural settlements or *veredas*: Pantanillo, Mata de Guadua and Guayabal. According to the information obtained in these workshops, the dominant illegal armed group in the area since the beginning of 2000 and until 2003 was the paramilitary group United Self-Defense Forces of Colombia (or AUC, from the name in Spanish), especially the Cacique Nutibara and Metro armed blocks. According to workshop participants, livestock farmers brought the AUC due to problems with petty crime. The presence of these groups was continuous due to the high strategic value of the area and the opportunity to extract rents from farmers. Between 2000 and 2002 there were confrontations between the paramilitary blocks, the FARC and Colombian National Army, leading to massive displacement.

During their time in the area, paramilitary groups instituted social and environmental rules. For instance, they made it mandatory to attend meetings of the Community Action Board (or JAC from the name in Spanish), and prohibited nightlife, drugs and the use of shotguns to hunt animals. According to workshop participants, the trust among community members decreased while these groups were present in the territory. Community leadership also deteriorated in comparison to the community order before the arrival of the AUC. For example, the Community Action Boards were split by ideological differences and the inhabitants resorted to the paramilitaries to solve everyday problems. The paramilitaries became the rulers of social and economic life, and the authorities for conflict resolution.

In 2003 there was a change of AUC control in the area (the Metro block lost its dominance to the Cacique block). In March, the IV Brigade (National Army) intensified military operations, and then in December of that year, the demobilization of the Bloque Cacique took place. Workshop participants reported that, after the demobilization of the AUC, petty crime increased. Participants also reported that despite the demobilization process, the paramilitary presence continued until 2005.

In 2011, Abejorral had the lowest number of homicides since 2007, and when the experiments were conducted, these communities were receiving state-driven aid for education and housing to mitigate the disastrous effects of the wet season.

### Betulia

We conducted three workshops in the *veredas* of Cibeles, La Valdivia and La Florida. According to information obtained, the FARC was the first armed group present in the area, arriving in 1980, and the paramilitaries arrived in the mid-1990s in order to gain territorial control. Unlike Abejorral, where the dominant armed group was the AUC, in Betulia constant violent encounters took place between the guerrillas and the Southwest and Metro paramilitary blocks until 2005, when the guerrillas withdrew from the area.

The strategic value of the area (its suitability for coca planting) was the main reason for the presence of different groups. In this process, the roles of the "informants" or "collaborators" were created on both sides. According to participants, this generated an atmosphere of uncertainty, distrust and terror.

According to workshop participants, before the presence of illegal armed groups, the Community Action Board and its president were a legitimate authority and they had a recognized regulatory function in social interactions within the community. However, with the arrival of armed actors, this social capacity to intervene was reduced through massacres, mass displacement, disappearances, targeted killings of peasants, accusations that leaders sympathized with the opposite side, and other coercion strategies.

According to workshop results, the Southwest block demobilized in 2008 and the following year the participants reported increases in petty crime until police arrival in the *veredas* in 2010.

In 2010, Betulia experienced a boom in coffee production, which was followed by a drastic drop in international coffee prices. At that time the FNC was setting up a support system to work with the farmers to deal with the emergency caused by low prices. When the experiments were carried out in late 2011, based upon participants’ reports and also official data, the level of violence had reached relatively low levels: the number of homicides was the lowest since the 1990s and the cases of mass displacement the lowest since 1997.

### San Jerónimo

We conducted workshops in the *veredas* of Buenos Aires, La Clarita and Alto Colorado. In Alto Colorado, the community identified the sporadic presence of paramilitary groups (including targeted killings and social cleansing for petty crime) during 1998 and until 2005-2006, when demobilization occurred. Nevertheless, according to workshop participants in that *vereda*, the community knew about those isolated cases when the paramilitaries came and went, but they never saw them again. In the other two *veredas*, presence or control by illegal armed groups was never identified at any moment in time.

In 2005, more projects were created, for example community organizations such as La Escuela Campesina (Rural School), the elderly group and also infrastructure projects like the shared irrigation system, the communal house and the road to La Mina.

In general and until 2011, San Jerónimo had achieved relatively low levels of violence. However, in 2010 the community experienced an isolated violent incident caused by the return of a family that had had problems years ago.

### Sopetrán

Here the workshops were held in Guayabal-Rojas and Guayabal-Los Pomos. Both *veredas* had a paramilitary presence from the mid-1990s until 2008. There are places within the municipality where these groups still remain.

According to participants, in the 1980s, gangs were created in town. Later, in 1995, police inspectors were removed and then the rumors of a paramilitary presence in Sopetrán started in 1998. According to some, paramilitaries arrived in the area brought by commercial traders to provide security. During this time, the JAC were weak and were not recognized as an authority. There were even rumors that some of the *vereda*’s leaders asked these groups for help because people were not participating in their meetings. With the arrival of the armed actors, a social purge took place. The paramilitaries gathered the community to warn them and tell them that they had certain rules, especially against theft and rape. The information obtained in the workshops suggests that paramilitary groups sought to create a social contract with the community by providing public goods such as roads and security against theft, and also by building a close relationship with the presidents of the Community Action Boards. In 2007, for example, paramilitary groups met with six or seven presidents of the JAC and they give away a cow for each *vereda*.

Even today, this armed group is recognized as an authority in town, to the point that they have an “office” in downtown Sopetrán where they solve the problems of the community. They reportedly act as rulers that provide security to the inhabitants.
