# Supplementary material for: Prosocial Behavior and Subjective Insecurity in Violent Contexts: Field Experiments
Source: PLoS One. 2016 Jul 29;11(7):e0158878. doi: 10.1371/journal.pone.0158878 (PMC4966936; doi:10.1371/journal.pone.0158878)
Supplement: S2 Text — (DOCX) [file pone.0158878.s011.docx]

**INFORMED CONSENT** Date: ____________________

You have been invited to participate in this exercise, which is part of a wider scientific research project.

This activity will not involve any risks. On the contrary, it could be beneficial, by giving you the opportunity to earn money. The amount of money you earn will depend on your decisions as well as on the decisions made by others. At the end of the activity you will be required to answer some questions. The amount of money that you earn during the exercise as well as the decisions you make will remain private. Your decision to participate is completely voluntary. You are free to withdraw from the activity at any moment. However, if you decide to withdraw, you will not receive any of the money.

I, ___________________________________________ declare that I understand the previously stated information as well as my rights and commitments as part of this activity. I am also aware that I can withdraw at any moment and waiver my right to claim any money I have earned.

Signed, ___________________________________________ National ID #___________

I, **Lina Moros**, researcher at *Universidad de los Andes*, hereby certify that this information will be used responsibly for academic and educative purposes. I also certify that each participant will be given the sum of money that they have earned during the exercise.

Signed, ______________________________________ National ID #___________
