# Supplementary material for: Prosocial Behavior and Subjective Insecurity in Violent Contexts: Field Experiments
Source: PLoS One. 2016 Jul 29;11(7):e0158878. doi: 10.1371/journal.pone.0158878 (PMC4966936; doi:10.1371/journal.pone.0158878)
Supplement: S3 Text — (DOCX) [file pone.0158878.s012.docx]

| **Survey respondent number**  (pre numerado Lina) |  | **Municipality**  **(Lina)** |  | |
| --- | --- | --- | --- | --- |
| **Game participated in.** | BP | C | D | |
| **Group number (BP)** |  |  |  | |
| **Group color (BP)** |  |  |  | |
| **Participant number (BP)** |  | **Date** |  | |
| **Participant letter (C and D)** |  | **Session** | AM | PM |
| **Researcher** |  | | | |

We have now finished the first part of today’s activity. While the researcher calculates your earnings, I am going to ask you a few questions. This survey is being undertaken by lecturers at *Universidad de los Andes*, and is being supported by the National Federation of Coffee Growers. We thank you both for your time and availability. The answers you give are completely anonymous and will only be analyzed aggregate, never individually.

1. **In which rural district and municipality do you live?**

Rural District: __________________________________________

Municipality: _________________________________________

1. **Of the following organizations, which do you have an association with?** Please use a scale of 1 to 3, 1 being no involvement and 3 being very active involvement.

|  |  | **No involvement** | **Occasional involvement** | **Active involvement** |
| --- | --- | --- | --- | --- |
|  |  | **1** | **2** | **3** |
| **A** | Premium coffee groups |  |  |  |
| **B** | Cooperative or other groups dedicated to coffee growing |  |  |  |
| **C** | Cooperative or other groups dedicated to non-coffee agricultural production |  |  |  |
| **D** | Any company (as a shareholder rather than an employee) |  |  |  |

1. **How active is your involvement with the organizations from this list?** **Please use a scale of 1 to 5, 1 being no involvement and 5 being very active involvement.**

|  |  | **No involvement** |  |  |  | **Very active involvement** | **Name** |
| --- | --- | --- | --- | --- | --- | --- | --- |
|  |  | **1** | **2** | **3** | **4** | **5** |  |
| **1** | **Environmental protection organizations** |  |  |  |  |  |  |
| **2** | Community water supply users associations |  |  |  |  |  |  |
| **3** | Senior citizen associations |  |  |  |  |  |  |
| **4** | Citizen oversight organizations |  |  |  |  |  |  |
| **5** | Choirs, music, theatre, or literary groups |  |  |  |  |  |  |
| **6** | Indigenous or Afro-Colombian organizations |  |  |  |  |  |  |
| **7** | Community mother associations |  |  |  |  |  |  |
| **8** | Savings groups |  |  |  |  |  |  |
| **9** | Sports teams |  |  |  |  |  |  |
| **10** | Groups of friends who get together from time-to-time |  |  |  |  |  |  |
| **11** | Community Action Councils |  |  |  |  |  |  |
| **12** | Women’s associations |  |  |  |  |  |  |
| **13** | Trade unions |  |  |  |  |  |  |
| **14** | Organizations to help those in need |  |  |  |  |  |  |
| **15** | Peasant Farmers’ Associations |  |  |  |  |  |  |
| **16** | Parents’ Associations |  |  |  |  |  |  |

*[****Note****: Only ask the following question to those people who answered 1 (No involvement) for question 3.1: involvement in an environmental protection organization]*

***3.a. Although you are not involved with an environmental protection organization, do you know people who are?***

*Yes With which organization? ________________*

*No (If NO move on to question 5)*

*[****Note****: Only ask the following questions to those people who answered* ***2 or higher to question 3.1*** *(involvement in an environmental protection organization) or* ***YES to question 3.a****]*

1. **Think of those people who you know who are involved with an environmental protection organization. Please answer to what extent these people would feel that they identify with the following sentences:**

|  |  | **To a not very great extent** | **To a great extent** |
| --- | --- | --- | --- |
| **1** | When I have a disagreement with people from the organization, I try to understand their points of view. | 1 | 2 |
| **2** | The things that happen to the other members of the organization move me. | 1 | 2 |
| **3** | I share the goals of other people from the organization. | 1 | 2 |
| **4** | When I notice that other people are taking advantage of someone from the organization, I want to protect them. | 1 | 2 |
| **5** | I feel sorry for the people from the organization when they have problems. | 1 | 2 |
| **6** | When I know that I am right, I don’t care about the arguments of the other members of the organization | 1 | 2 |
| **7** | Other organization members’ bad luck worries me. | 1 | 2 |
| **8** | Before criticizing anyone from the organization, I try to imagine how I would feel in his or her place. | 1 | 2 |

1. **Please rank the following statements from 1 to 4, 1 being categorically NO and 4 being categorically YES.**

|  | | **Categorically NO** | **Sometimes NO** | **Sometimes YES** | **Categorically YES** |
| --- | --- | --- | --- | --- | --- |
|  |  |  |  |  |  |
|  |  | **1** | **2** | **3** | **4** |
| **A** | I feel safe when going out at night |  |  |  |  |
| **B** | I think that I could be threatened |  |  |  |  |
| **C** | I feel that my life is in danger |  |  |  |  |
| **D** | I feel that I run risks when I take part in different types of meetings (commercial, political, religious, social). |  |  |  |  |
| **E** | I am afraid of day-time robberies |  |  |  |  |
| **F** | I am afraid of night-time robberies |  |  |  |  |
| **G** | I am afraid of day-time aggression |  |  |  |  |
| **H** | I am afraid of night-time aggression |  |  |  |  |

1. **How often do you currently participate in the following organizations? Use a range of 1 to 5, 1 representing no involvement and 5 very active involvement.**

|  |  | **I am not involved** |  |  |  | **I am very actively involved** | **Name** |
| --- | --- | --- | --- | --- | --- | --- | --- |
|  |  | **1** | **2** | **3** | **4** | **5** |  |
| **1** | Municipal planning council |  |  |  |  |  |  |
| **2** | Church, parish, worship or prayer group |  |  |  |  |  |  |
| **3** | Political party or movement |  |  |  |  |  |  |
| **4** | Participatory groups that are promoted by the town hall or local government |  |  |  |  |  |  |
| **5** | Other social groups that are promoted by the Federation of Coffee Growers |  |  |  |  |  |  |
| **6** | Others  Which ones?_________________________________________________________________ | | | | | |  |

1. **Would you say that the inhabitants of your rural district:**

1. Help each other a lot

2. Help each other a little

3. Do not help each other at all

| 1. **How many people do you live with in your home, not including yourself.** |  |
| --- | --- |

1. **Please rank the following statements from 1 to 4, 1 being categorically NO and 4 being categorically YES. The following questions concern the security of the members of your household.**

*[In the case that the person lives alone, make a note that this question does not apply]*

|  |  | **Categorically NO** | **Sometimes NO** | **Sometimes YES** | **Categorically YES** |
| --- | --- | --- | --- | --- | --- |
|  |  |  |  |  |  |
|  |  | **1** | **2** | **3** | **4** |
| **A** | I feel that the members of my household are safe when they go out at night |  |  |  |  |
| **B** | I feel that the members of my household could be threatened |  |  |  |  |
| **C** | I feel that the lives of the members of my household are in danger |  |  |  |  |
| **D** | I feel that the members of my household are at risk if they take part in different types of meetings (commercial, political, religious, social). |  |  |  |  |
| **E** | The members of my household are afraid of day-time robberies |  |  |  |  |
| **F** | The members of my household are afraid robberies at night |  |  |  |  |
| **G** | The members of my household are afraid of day-time aggressions |  |  |  |  |
| **H** | The members of my household are afraid of night-time aggressions |  |  |  |  |

1. **To what extent do you trust the people from the following groups:**

|  |  | **Complete trust** | **Partial trust** | **Little trust** | **No trust** | Don’t know  [DO NOT READ OUT] | Does not answer  [DO NOT READ OUT] |
| --- | --- | --- | --- | --- | --- | --- | --- |
| **1** | Your family | 4 | 3 | 2 | 1 | 0 | 0 |
| **2** | Your neighbors | 4 | 3 | 2 | 1 | 0 | 0 |
| **3** | People you know | 4 | 3 | 2 | 1 | 0 | 0 |
| **4** | People you have met for the first time | 4 | 3 | 2 | 1 | 0 | 0 |
| **5** | People who have a different religion | 4 | 3 | 2 | 1 | 0 | 0 |
| **6** | Foreigners | 4 | 3 | 2 | 1 | 0 | 0 |

1. **Rank the following statements from 1 to 4, 1 being categorically NO and 4 being categorically YES. The following questions are concerned with the security of your neighbors in your rural district.**

|  |  | **Categorically NO** | **Sometimes NO** | **Sometimes YES** | **Categorically YES** |
| --- | --- | --- | --- | --- | --- |
|  |  |  |  |  |  |
|  |  | **1** | **2** | **3** | **4** |
| **1** | I feel that my neighbors are safe when they go out at night |  |  |  |  |
| **2** | I feel that my neighbors could be threatened |  |  |  |  |
| **3** | I feel that my neighbors’ lives are in danger |  |  |  |  |
| **4** | I feel that my neighbors are at risk if they take part in different types of meetings (commercial, political, religious, social). |  |  |  |  |
| **5** | My neighbors are afraid of robberies |  |  |  |  |
| **6** | My neighbors are afraid of aggressions |  |  |  |  |
| **7** | My neighbors feel that there is an authority that protects them |  |  |  |  |
| **8** | My neighbors feel that their children can play peacefully in the area in which we live |  |  |  |  |

1. **In general, would you say that the majority of people can be trusted, or that it is necessary to be cautious when dealing with people?**

1. The majority of people can be trusted

2. It is necessary to be very cautious when dealing with people

0. Don’t know *[DO NOT READ OUT]*

1. **What is your household’s income?** *[Remember to state the period]*

| **Amount** |  |
| --- | --- |
| **Period**  (Per day, week, month) |  |

1. **What is the main activity you currently undertake, to which you dedicate the majority of your time?**

*[****Note:*** *in theory only one option should be ticked. However, if someone can definitively not decide, then it is acceptable to tick two.]*

| **Coffee grower** |  |
| --- | --- |
| **Non-coffee farmer** |  |
| **Cattle ranching** |  |
| **Homemaker** |  |
| **Student** |  |
| **Other. What? ________________** | |

1. **In the past 12 months have you been the victim of any of the following:**

|  | **YES** | **NO** |
| --- | --- | --- |
| 1. Robbery without firearm |  |  |
| 1. Armed robbery |  |  |
| 1. Physical aggression or sexual violence |  |  |
| 1. Kidnapping |  |  |
| 1. Street fight |  |  |
| 1. Family violence or violent family dispute |  |  |
| 1. Damage to your property, your crops, or your animals |  |  |
| 1. Extortion or ‘illegal taxes’ |  |  |
| 1. Verbal aggression |  |  |
| 1. Harassment |  |  |
| 1. Persecution |  |  |
| 1. Psychological bullying |  |  |
| 1. Death threats |  |  |
| 1. Another form of violence. What? _______________ |  |  |

1. Have you ever been the victim of:

a. Forced displacement YES NO

b. Rape YES NO

1. **Have you ever witnessed the murder of one or more than one person?**

Yes No

1. **Are you, or is someone living with you a landowner?** Yes No
2. **If you or someone living with you is a landowner, what is the combined total size of your land?**

| **Size** | **Unit of land area (blocks, hectares, varas, etc.)** |
| --- | --- |
|  |  |

1. **If you are a property owner, what is the legal situation regarding your land?**

|  |  | Plot 1 | Plot 2 | Plot 3 |
| --- | --- | --- | --- | --- |
| **1** | Your ownership is written in a deed, and it is registered in the registry office |  |  |  |
| **2** | Your ownership is written in a deed, but it is not registered in the registry office |  |  |  |
| **3** | Your ownership is written in a deed, but it is in the process of succession |  |  |  |
| **4** | You have verbal or written evidence that the piece of land is yours, but it does not have a deed |  |  |  |
| **5** | You are in possession of the piece of land, but it does not have any documentation |  |  |  |
| **6** | Other What? _____________________ |  |  |  |
| **7** | I don’t know |  |  |  |

1. **Do you have a coffee growers’ ID card?**

| **Yes, in my name** | **No, but a member of my household does** | **The application is currently being processed** | **No** |
| --- | --- | --- | --- |
|  |  |  |  |

| **Female** | **Male** |
| --- | --- |
|  |  |

1. **Sex:**
2. **How old are you?**
3. **What is your level of education? Please specify the year in which you finished.**

*[The researcher fills out the table with the person’s answer. Government validation of the high school diploma is equivalent to the same amount of years in formal education. The same applies to high school diploma undertaken via the radio. Remember to ask about technical qualifications and university qualifications.]*

| **Level** | **Year** |
| --- | --- |
| None |  |
| Primary (Specify the last year that was completed) |  |
| High school diploma (Specify the last year that was completed) |  |
| Unfinished technical qualification |  |
| Completed technical qualification |  |
| Unfinished university qualification |  |
| Completed university qualification |  |
| Unfinished postgraduate qualification |  |
| Completed postgraduate qualification |  |

1. **How many people that came to this activity today do you know personally?** ___________
2. **Three years ago, did you participate in a similar activity to this in which you were able to earn money for decisions you made?**

Yes No

1. **Have you heard about the Huellas de Paz agreement?**

[**Note:** Questions *28 and 29 are only for the Betulia and Sopetrán municipalities*]

1. **Have you or any member of your family ever been involved in Huellas de Paz?**

| **I haven’t been involved** | **I am sometimes involved** | **I am actively involved** |
| --- | --- | --- |
| **1** | **2** | **3** |

1. **Through my participation in Huellas de Paz:**

1. I am more aware of the need to support and help members of my community.
2. I have found out that I can be a leader in my community (or that I have the skills to lead in my community).
3. I have extended the circle of people I associate with in in my community.
4. I am better trained and prepared to suggest projects for my community.
5. I have been able to better understand and exercise my rights as a person and member of a community.
6. I have had the chance to actively be involved in community spaces that have been suggested by Huellas de Paz (inter-institutional events, community media).
