# Supplementary material for: Prosocial Behavior and Subjective Insecurity in Violent Contexts: Field Experiments
Source: PLoS One. 2016 Jul 29;11(7):e0158878. doi: 10.1371/journal.pone.0158878 (PMC4966936; doi:10.1371/journal.pone.0158878)
Supplement: S4 Text — (DOCX) [file pone.0158878.s013.docx]

**INSTRUCTIONS PUBLIC GOODS GAME**

Good morning / afternoon,

Thank you for accepting our invitation to take part in this activity. Today’s activity is part of a research project that is being carried out by researchers at *Universidad de los Andes*. The funding for this project comes from an international institution. Today there are *x* researchers working on the following activities: *xxx*, *xxx*, and *xxx*.

Before starting the activity, we are going to give all of you $10,000 to contribute towards your transport costs. This is your money, so please keep it.

**(RESEARCHER GIVES OUT MONEY TO EACH PARTICIPANT)**

We shall now commence reading the rules in order to explain the activity you will be involved in today.

The objective of this activity is to understand how people make decisions; so we will learn from the decisions you make. Bear in mind that all the decisions you make during this activity, and any information you provide us with, will remain confidential. The only people who will have access to the information are the researchers involved in the project; we will not provide it to any other person.

During the activity you will have the opportunity to earn money. The amount of money you earn will depend on your decisions and the decisions of others. We do not know how much you will earn, but it could be between **22,000 pesos and 45,000** pesos.

The total amount of money that you earn will be rounded up or down to the closest $1,000; for example, if you earn $18,400 pesos or less, the figure will be rounded down to $18,000, and if you earn $18,500 pesos or more, the figure will be rounded up to $19,000 pesos.

Does everyone understand?

We use money in this activity because in real life your decisions have financial consequences. Any money you earn today is yours. Only the research team will know how much you have earned.

This activity may differ from activities other members of your community have taken part in previously. Therefore, comments or explanations you might have heard about it are unlikely to apply to the activity in which you will participate today.

**Today’s activity will last 3 hours.** Your participation is absolutely voluntary and you are free to withdraw from it at any point. However, if you do leave, other participants will have to do so also, as we need an even number of people. In order to receive payment you must stay until the end of the activity. **If** **you cannot stay for the full 3 hours, please let us know now.**

**Is there anyone who cannot stay for the full 3 hours?**

This activity will not involve any risks. On the contrary, it could benefit you by allowing you the opportunity to earn money. Are you willing to participate?

If you are indeed willing, please read and sign the form that the researcher is currently handing out.

**(RESEARCHER HANDS OUT INFORMED CONSENT FORM)**

**(RESEARCHER READS CONSENT FORM)**

**INFORMED CONSENT FORM** Date: ____________________

You have been invited to participate in this exercise, which is part of a wider scientific research project.

This activity will not involve any risk. On the contrary, it could be beneficial, by giving you the opportunity to earn money. The amount of money you earn will depend on your decisions as well as on the decisions made by others. At the end of the activity you will be required to answer some questions. The amount of money that earn during the exercise as well as the decisions you make will remain private. Your decision to participate is completely voluntary. You are free to withdraw from the activity at any moment. However, if you decide to withdraw, you will not receive any of the money.

I, ___________________________________________ declare that I understand the previously stated information as well as my rights and commitments as part of this activity. I am also aware that I can withdraw at any moment and waiver my right to claim any money I have earned.

Signed, ___________________________________National ID #_________

I, **Lina Moros**, researcher at *Universidad de los Andes*, hereby certify that this information will be used responsibly for academic and educative purposes. I also certify that each participant will be given the sum of money that they have earned during the exercise.

Signed, ___________________________________National ID #_________

At this point a researcher will collect your consent form from you.

**(RESEARCHER COLLECTS INFORMED CONSENT FORM)**

We are now going to begin reading the instructions so that you are able to participate in the activity. Please do not speak to the other participants. If you speak with other people you will interrupt the activity and make it harder for others to understand the instructions. It might even spoil the activity to the extent that it has to be cancelled. We also ask you to put your cell phones on silent so they do not interrupt the activity.

If you have any questions please raise your hand. On no account ask a question out loud. All doubts will be resolved one-to-one.

Please pay careful attention to these instructions.

The activity in which you are going to take part today seeks to simulate a situation in which a group of individuals needs to make a decision about how to carry out a project that will benefit the entire community **(for example, repairing a path that everyone in the community uses).**

**The activity in which you are going to take part has a total of 15 rounds.**

In each round, **each participant** will be required to make decisions similar to those you make when deciding between something that would benefit only you and your family or the whole of your community.

You are going to participate in a group of 5 people, **but the decisions you make will be your own.** Today, 4 groups will be working at the same time. Each group is independent and the **decisions made by other groups do not affect the decisions made by your group at all.** Each group will be distinguishable from the other by the color of the sheets of paper they use during the activity.

Participants start **every round with 1,500 pesos.**

You have to decide how much of this money you wish to keep and how much you wish to contribute to the group project. The project benefits **everyone in the group**, regardless of how much each individual contributes.

The overall amount of money contributed by the 5 participants **(your money, plus the contributions of the 4 other group members)** will be doubled by the researchers. This total will then be divided equally between the 5 participants in the group, **irrespective of the amount each has contributed**.

At the end of each round your earnings will be the sum of:

1. **The amount of money you kept for yourself.**
2. **The amount of money you receive from the project.**

For example, imagine that the total amount of money contributed by the members of the group (your contribution and the contributions of the other 4 members) is **4,000** pesos. We will double this **4,000** pesos for a total of **8,000** pesos, which will then be divided by 5. Therefore, each member of the group will receive 1,600 pesos, plus the sum of money that s/he kept back.

**The individual earnings** made in each round will be calculated and paid out in cash at the end of Round 15.

**Don’t worry if you don’t understand – later on we are going to provide some examples and do some practice rounds.**

Now I am going to explain how to record the decisions you make in each round.

In each round you will complete a **“decision sheet,”** recording the money you keep and the amount you are going to contribute to the group project.

The decision sheets are sheets of paper like **this**, which you will receive before starting the activity.

**(SHOW THE POSTER FOR THE DECISION SHEET)**

Each decision sheet has a participant identification number on it. **This will be your identification number throughout the entire activity.**

You will begin each round with 1,500 pesos, and you will be required to write **three things** on your decision sheet:

1. **The number of the current round**, which will be announced by the researcher.
2. **The “Amount I keep”:** this is the amount of money you are going to keep (between 0 and 1,500 pesos)
3. **The “Contribution to the project”:** this is the amount of money you wish to contribute to the group project (also between 0 and 1,500 pesos)

Therefore, the total of “Amount I keep” and “Contribution to the project” should equal 1,500 pesos for each round.

After all 5 group participants have made and recorded their decisions, one of the researchers will collect their decision sheets and add up the contributions **made to the group project** by all the members of the group.

This amount is multiplied by 2 and then divided by 5 to calculate the individual amount earned by each member of the group.

The researcher announces the **total amount of money contributed to the group, multiplied by 2,** and the amount that each group member will receive for each round.

**It should be stressed that the decisions made by members are confidential and that you should not inform any other group member the decision you have made.**

**Does anyone have any questions? If you do, please raise your hand and a researcher will answer them one-to-one.**

**(THE RESEARCHER HANDS OUT THE RECORD SHEET TO EACH PARTICIPANT)**

At this point the researchers distribute a **record sheet**, which will be used to record the decisions made, and the amount of money that each participant earns in each round. The sheet contains three examples showing how it should be filled in.

We shall provide three examples so you understand how to use this sheet.

**Remember that these are simply examples.** In each round you are free to decide **how much money you want to keep** (between 0 and 1,500 pesos) and **how much you wish to contribute to the project** (also between 0 and 1,500 pesos).

Please pay careful attention to the explanation:

**(THE RESEARCHER SHOWS THE POSTER OF THE RECORD SHEET)**

Let’s look at the **first example** in order to see how to use the record sheet. The first example appears in the first row of the record sheet shown on the poster and on the sheet you have in your hands.

Remember that at the beginning of each round **you will have** **1,500 pesos**. This amount should be written in **Column A.** You should do **two things** with this 1,500 pesos**:**

1. “**Amount I keep”:** this is the amount of money you are going to keep (between **0 and 1,500** pesos)
2. **“Contribution to the project”:** this is the amount of money you wish to contribute to the group project (also between **0 and 1,500** pesos)

Remember that in every round **“Amount I keep” + “Contribution to the project”** should equal 1,500 **pesos**.

Suppose that in this example you decide to keep **1,200pesos.**

You should write **1,200** pesos in **Column B** of the record sheet: “Amount I keep”.

If you decide to keep **1,200pesos** you are therefore contributing **300** pesos to the project **(1,500 pesos - 1,200pesos = 300 pesos**).

You should write this amount in **Column C of the** record sheet, “Contribution to the project”.

Remember that you should record the amounts for “Amount I keep” and “Contribution to the project” in two places**: on the record sheet and on the decision sheet** (Please ensure you write the same amount on both sheets).

Remember that these are just examples. In each round, you are free to decide how much money to keep and how much to contribute to the project.

When all the participants have decided, one of the researchers will note down the decisions of all **5 participants** and add up the amount each one contributed to the project, in order to calculate the  **“Total amount contributed to the project**” – that is, the money you contributed, plus what the other four members of the group contributed.

This amount will then be announced to the group by the researcher and should be written down in **Column D** of your record sheet. In this case, we are assuming the amount to be **2,500 pesos.**

Next, the researcher multiples this total by two. In this example, the total will be **5,000 pesos (2,500 pesos x 2).**

This amount is then announced by the researcher to the group and you should write it down in **Column E,** “**Total amount contributed to the project x 2**”.

These **5,000** pesos are shared out equally between the 5 participants in the group. This means that in this example each participant receives **1,000 pesos, regardless of how much money s/he contributed to the project**. As you can see, **1,000 pesos** is written in **Column F,** “My money earned thanks to the project".

Now, in order to calculate your earnings for the round, you should add **Column B** (“Amount I keep”) and Column **F** (“My money earned thanks to the project”). In this example, your earnings are **1,200 pesos + 1,000 pesos = 2,200pesos**. As you can see, this amount is written in **Column G**, **“Overall total earned in this round”.**

**Does anyone have any questions? If you do, please raise your hand and a researcher will answer them one-to-one.**

**Now let’s look at a second example.**

Remember that at the beginning of each round **you will have** **1,500 pesos**. This amount should be written in **Column A.**

Suppose that in this example you decide to keep **300 pesos.**

You should write **300 pesos** in **Column B** of the record sheet: “Amount I keep”.

If you decide to keep **300 pesos,** you are therefore contributing **1,200** pesos to the project **(1,500 pesos - 300 pesos = 1,200pesos).**

You should write this amount in **Column C of the** record sheet, “Contribution to the project”.

Remember that you should record the amounts for “Amount I keep” and “Contribution to the project” in two places**: on the record sheet and on the decision sheet** (Please ensure you write the same amount on both sheets).

Remember that **these are examples**. In each round, **you are free to decide** how much money to keep and how much to contribute to the project.

When all the participants have decided, one of the researchers will collect the decisions of all **5 participants** and add up the amount each one contributed to the project, in order to calculate  **“Total amount contributed to the project**” – that is, the money you contributed, plus what the other four members of the group contributed.

This amount will then be announced to the group by the researcher and should be written down in **Column D** of your record sheet. In this case we shall assume the amount to be **8,000 pesos.**

Next, the researcher multiples the total by two. In this example the total will be **16,000 pesos (8,000 pesos x 2).**

This amount is the announced by the researcher to the group and you should write it down in **Column E,** “**Total amount contributed to the project x 2**”.

These **16,000** pesos are **shared out equally between the 5 participants in the group**. This means that in this example each participant receives **3,200** pesos, regardless of how much money s/he contributed to the project. As you can see, **3,200 pesos** is written in **Column F,** “My money earned thanks to the project".

Now, in order to calculate your earnings for the round, you should add **Column B** (“Amount I keep”) and Column **F** “My money earned thanks to the project”. In this example, your earnings are **300 pesos + 3,200 pesos = 3,500 pesos**. As you can see, this amount is written in **la Column G**, **“Overall total earned in this round”.**

**Does anyone have any questions? If you do, please raise your hand and a researcher will answer them one-to-one.**

**Don’t worry if you don’t understand – later on we are going to provide some examples and do some practice rounds.**

Before starting, we are going to hand out a sheet of paper containing some questions we would like you to answer, in order to ensure that **you have understood the** **instructions**.

This is not the exercise, so you still do not need to make any decisions. Remember that you should not speak with anyone during the activity. When you finish please raise your hand and a researcher will come over and check your answers.

Please answer the questions now.

**(RESEARCHER DISTRIBUTES THE QUESTIONS)**

Please fill in the blank space with the correct answer:

Remember that you will start each round with 1,500 pesos.

Suppose that you decide to contribute 1,000 pesos to the project

**How much money would you keep?**_500 pesos____

The researcher announces that the total amount of money contributed to the project by the participants (your money plus that of the other 4 members of the group) is 4,500 pesos.

The researcher multiplies this amount by 2 (in this example, twice the amount contributed equals 9,000) and then divides it equally between the 5 participants. Thus, in this example each participant will receive:

__ 1,800 __pesos from the project.

**What are your total earnings from this round?**  2,300___ pesos

**(THE RESEARCHER COLLECTS AND CHECKS EACH SET OF ANSWERS. WHEN AN ANSWER IS INCORRECT THE RESEARCHER EXPLAINS BRIEFLY WHY. IF, FOLLOWING THE EXPLANATION, S/HE SUSPECTS THAT THE PERSON DOES NOT FULLY UNDERSTAND THE REASONS THIS SHOULD BE NOTED ON THE OBSERVATIONS SHEET)**

We are now ready to begin. We are going to divide you into groups of 5. Please remain silent.

**(THE RESEARCHER ORGANIZES THE GROUPS SO THAT THEY ARE SEPARATED FROM EACH OTHER. IDEALLY, IF CHAIRS WITH FOLDING DESKS ARE AVAILABLE THE GROUPS SHOULD BE ARRANGED IN CIRCLES WITH THEIR BACKS TO THE OTHER GROUPS. ONCE THE GROUPS HAVE BEEN FORMED THE DECISION SHEETS ARE HANDED OUT AND THE MEANING OF THE IDENTIFICATION NUMBERS EACH HAS BEEN ASSIGNED IS EXPLAINED)**

At this point a researcher will distribute the decision sheets, your record sheet and your identification number.

**(RESEARCHER DISTRIBUTES THE DECISION SHEETS, YOUR RECORD SHEET AND YOUR IDENTIFICATION NUMBER)**

Please write your identification number at the top of the sheet.

We are going to do two practice rounds that will not affect your overall earnings but that provide an important opportunity to practice the activity.

**Instructions for practice Round 1**

Please write down the number of the round (“P1”), the amount of money you are going to keep, and the amount you are going to contribute to the project.

These amounts should be registered both on the record sheet and on the decision sheet. Please wait for the researcher to collect your decision sheet.

Now the researcher announces the totals for “Total amount contributed to the project”, “Total contributed x 2” and “My money earned thanks to the project”.

We have now completed practice Round 1.

**Instructions for practice Round 2**

**We are now going to start practice Round 2.**

Please write down the number of the round (“P2”), the amount of money you are going to keep, and the amount you are going to contribute to the project.

These amounts should be registered both on the record sheet and on the decision sheet. Please wait for the researcher to collect your decision sheet.

Now the researcher announces the totals for “Total amount contributed to the project”, “Total contributed x 2” and “My money earned thanks to the project”.

We have now completed practice Round 2

**Instructions for practice Round 3**

**We are now going to start practice Round 3**

Please write down the number of the round (“P3”), the amount of money you are going to keep, and the amount you are going to contribute to the project.

These amounts should be registered both on the record sheet and on the decision sheet. Please wait for the researcher to collect your decision sheet.

Now the researcher announces the totals for “Total amount contributed to the project”, “Total contributed x 2” and “My money earned thanks to the project”.

We have now completed practice Round 3

**Instructions Round 1.**

**We are now going to start Round 1.** From this round onwards, your earnings will be counted. Remember to write the number of the round on your decision sheet.

Please write down the number of the round (“1”), the amount of money you are going to keep, and the amount you are going to contribute to the project.

These amounts should be registered both on the record sheet and on the decision sheet. Please wait for the researcher to collect your decision sheet.

Now the researcher announces the totals for “Total amount contributed to the project”, “Total contributed x 2” and “My money earned thanks to the project”.

We have now completed Round 1.

**Instructions Round 2.**

**We are now going to start Round 2.**

Please write down the number of the round (“2”), the amount of money you are going to keep, and the amount you are going to contribute to the project.

These amounts should be registered both on the record sheet and on the decision sheet. Please wait for the researcher to collect your decision sheet.

Now the researcher announces the totals for “Total amount contributed to the project”, “Total contributed x 2” and “My money earned thanks to the project”.

We have now completed Round 2.

**Instructions Round 3.**

**We are now going to start Round 3.**

Please write down the number of the round (“3”), the amount of money you are going to keep, and the amount you are going to contribute to the project.

These amounts should be registered both on the record sheet and on the decision sheet. Please wait for the researcher to collect your decision sheet.

Now the researcher announces the totals for “Total amount contributed to the project”, “Total contributed x 2” and “My money earned thanks to the project”.

We have now completed Round 3.

**Instructions Round 4.**

**We are now going to start Round 4.**

Please write down the number of the round (“4”), the amount of money you are going to keep, and the amount you are going to contribute to the project.

These amounts should be registered both on the record sheet and on the decision sheet. Please wait for the researcher to collect your decision sheet.

Now the researcher announces the totals for “Total amount contributed to the project”, “Total contributed x 2” and “My money earned thanks to the project”.

We have now completed Round 4.

**Instructions Round 5.**

**We are now going to start Round 5.**

Please write down the number of the round (“5”), the amount of money you are going to keep, and the amount you are going to contribute to the project.

These amounts should be registered both on the record sheet and on the decision sheet. Please wait for the researcher to collect your decision sheet.

Now the researcher announces the totals for “Total amount contributed to the project”, “Total contributed x 2” and “My money earned thanks to the project”.

We have now completed Round 5.

**Instructions Round 6.**

**We are now going to start Round 6.**

Please write down the number of the round (“6”), the amount of money you are going to keep, and the amount you are going to contribute to the project.

These amounts should be registered both on the record sheet and on the decision sheet. Please wait for the researcher to collect your decision sheet.

Now the researcher announces the totals for “Total amount contributed to the project”, “Total contributed x 2” and “My money earned thanks to the project”.

We have now completed Round 6.

**Instructions Round 7.**

**We are now going to start Round 7.**

Please write down the number of the round (“7”), the amount of money you are going to keep, and the amount you are going to contribute to the project.

These amounts should be registered both on the record sheet and on the decision sheet. Please wait for the researcher to collect your decision sheet.

Now the researcher announces the totals for “Total amount contributed to the project”, “Total contributed x 2” and “My money earned thanks to the project”.

We have now completed Round 7.

**Instructions Round 8.**

**We are now going to start Round 8.**

Please write down the number of the round (“8”), the amount of money you are going to keep, and the amount you are going to contribute to the project.

These amounts should be registered both on the record sheet and on the decision sheet. Please wait for the researcher to collect your decision sheet.

Now the researcher announces the totals for “Total amount contributed to the project”, “Total contributed x 2” and “My money earned thanks to the project”.

We have now completed Round 8.

**Instructions Round 9.**

**We are now going to start Round 9.**

Please write down the number of the round (“9”), the amount of money you are going to keep, and the amount you are going to contribute to the project.

These amounts should be registered both on the record sheet and on the decision sheet. Please wait for the researcher to collect your decision sheet.

Now the researcher announces the totals for “Total amount contributed to the project”, “Total contributed x 2” and “My money earned thanks to the project”.

We have now completed Round 9.

**Instructions Round 10.**

**We are now going to start Round 10.**

Please write down the number of the round (“10”), the amount of money you are going to keep, and the amount you are going to contribute to the project.

These amounts should be registered both on the record sheet and on the decision sheet. Please wait for the researcher to collect your decision sheet.

Now the researcher announces the totals for “Total amount contributed to the project”, “Total contributed x 2” and “My money earned thanks to the project”.

We have now completed Round 10.

**Instructions Round 11.**

**We are now going to start Round 11.**

Please write down the number of the round (“11”), the amount of money you are going to keep, and the amount you are going to contribute to the project.

These amounts should be registered both on the record sheet and on the decision sheet. Please wait for the researcher to collect your decision sheet.

Now the researcher announces the totals for “Total amount contributed to the project”, “Total contributed x 2” and “My money earned thanks to the project”.

We have now completed Round 11.

**Instructions Round 12.**

**We are now going to start Round 12.**

Please write down the number of the round (“12”), the amount of money you are going to keep, and the amount you are going to contribute to the project.

These amounts should be registered both on the record sheet and on the decision sheet. Please wait for the researcher to collect your decision sheet.

Now the researcher announces the totals for “Total amount contributed to the project”, “Total contributed x 2” and “My money earned thanks to the project”.

We have now completed Round 12.

**Instructions Round 13.**

**We are now going to start Round 13.**

Please write down the number of the round (“13”), the amount of money you are going to keep, and the amount you are going to contribute to the project.

These amounts should be registered both on the record sheet and on the decision sheet. Please wait for the researcher to collect your decision sheet.

Now the researcher announces the totals for “Total amount contributed to the project”, “Total contributed x 2” and “My money earned thanks to the project”.

We have now completed Round 13.

**Instructions Round 14.**

**We are now going to start Round 14.**

Please write down the number of the round (“14”), the amount of money you are going to keep, and the amount you are going to contribute to the project.

These amounts should be registered both on the record sheet and on the decision sheet. Please wait for the researcher to collect your decision sheet.

Now the researcher announces the totals for “Total amount contributed to the project”, “Total contributed x 2” and “My money earned thanks to the project”.

We have now completed Round 14.

**Instructions Round 15.**

**We are now going to start Round 15.**

Please write down the number of the round (“15”), the amount of money you are going to keep, and the amount you are going to contribute to the project.

These amounts should be registered both on the record sheet and on the decision sheet. Please wait for the researcher to collect your decision sheet.

Now the researcher announces the totals for “Total amount contributed to the project”, “Total contributed x 2” and “My money earned thanks to the project”.

**Instructions for the end of Round 15**

**(WHEN THE 15 ROUNDS HAVE ALL BEEN COMPLETED THE SUPERVISING RESEARCHER WILL READ OUT THE FOLLOWING PARAGRAPH)**

We have now completed **Round** **15, and the activity has finished**. Please do not speak to anyone. A researcher will collect your record sheet and ask you some questions. While you are answering the questions, the researcher will calculate your earnings. After answering all the questions, we will call each individual and give them their earnings in cash.
