# Supplementary material for: Prosocial Behavior and Subjective Insecurity in Violent Contexts: Field Experiments
Source: PLoS One. 2016 Jul 29;11(7):e0158878. doi: 10.1371/journal.pone.0158878 (PMC4966936; doi:10.1371/journal.pone.0158878)
Supplement: S5 Text — (DOCX) [file pone.0158878.s014.docx]

**INSTRUCTIONS: TRUST**

Good morning / afternoon,

Thank you for accepting our invitation to take part in this activity. Today’s activity is part of a research project that is being carried out by researchers at *Universidad de los Andes*. The funding for this project comes from an international institution. Today there are *x* researchers working on the following activities: *xxx*, *xxx*, and *xxx*.

Before starting the activity, we are going to give all of you $10,000 to contribute towards your transport costs. This is your money, so please keep it.

**(RESEARCHER GIVES OUT MONEY TO EACH PARTICIPANT)**

We will now begin to read the instructions in order to explain the activity in which you will be taking part.

The objective of this activity is to find out how people make decisions; thus, we will learn by observing you decide. All the decisions that you make during this activity, as well as any other information that you provide us with, will remain confidential. The only people with access to the information will be the researchers working on this project; it will not be given to any one else.

You can earn money by taking part in this activity. The amount of money that you earn depends on the decisions you make as well on the decisions that others make. We do not know the exact amount that you will earn; however, it will be somewhere between **$0 and $72,000** pesos**.**

The total amount of money that you earn will be rounded up or down to the closest $1,000; for example, if you earn $18,400 pesos or less, the figure will be rounded down to $18,000, and if you earn $18,500 pesos or more, the figure will be rounded up to $19,000 pesos.

Does everyone understand?

We will use money in this activity because as in real life, your decisions will have economic consequences. The money that you earn in today’s activity is yours, and only the researchers will know the exact amount that you have earned.

This activity may be different to other activities in which members of your community have participated. As such, any comment or explication that you may have heard regarding today’s activity may well not be pertinent.

**Today’s activity will last three hours.** Your participation is completely voluntary and you are free to withdraw at any moment. However, if you do withdraw other participants will also be forced to withdraw as we need an equal number of participants. In order to receive the money that you have earned, you will have to stay until the end of the activity. **If anyone is unable to stay until the end of the activity, please advise us now.**

**Is anyone unable to stay for the full three hours?**

This activity will not involve any risks. On the contrary, it could benefit you by allowing you the opportunity to earn money. Are you willing to participate?

If you are indeed willing, please read and sign the form that the researcher is currently handing out. **(RESEARCHER HANDS OUT INFORMED CONSENT SHEETS)**

**(RESEARCHER READS INFORMED CONSENT SHEETS)**

**INFORMED CONSENT FORM** Date: ____________________

You have been invited to participate in this exercise, which is part of a wider scientific research project.

This activity will not involve any risk. On the contrary, it could be beneficial, by giving you the opportunity to earn money. The amount of money you earn will depend on your decisions as well as on the decisions made by others. At the end of the activity you will be required to answer some questions. The amount of money that earn during the exercise as well as the decisions you make will remain private. Your decision to participate is completely voluntary. You are free to withdraw from the activity at any moment. However, if you decide to withdraw, you will not receive any of the money.

I, ___________________________________________ declare that I understand the previously stated information as well as my rights and commitments as part of this activity. I am also aware that I can withdraw at any moment and waiver my right to claim any money I have earned.

Signed, ________________________________National ID #____________

I, **Lina Moros**, lecturer at *Universidad de los Andes*, hereby certify that this information will be used responsibly for academic and educative purposes. I also certify that each participant will be given the sum of money that they have earned during the exercise.

Signed, ________________________________National ID #____________

We are now going to read the instructions in order for you to participate in the activity. **Please do not speak to other participants.** If you speak to others you will disrupt the activity and make it more difficult for others to understand the instructions. You may also ruin the activity and it will have to be cancelled. We also request that you turn your phones on to silent so that the activity is not interrupted.

Please pay attention to these instructions.

The activity in which you are about to participate **will be carried out in pairs**. Each person will be playing with someone else who will be selected randomly. However, no one will know the identity of the person with whom they are playing; only the researchers will know who the pairs are.

**You will play six rounds.** In each round you will either play with the same partner as in the previous round or with a new partner; we will tell you which at the beginning of each round.

Each pair will be made up of a **participant A and a participant B**. What letter participant you are will be determined by a draw. Each participant will also be given an identification number.

From this moment onwards you should speak to no one. If anyone has a question, then please raise your hand. Under no circumstances ask the question aloud. Any questions will be individually answered. **(RESEARCHER TO SHOW POSTER 1)**

I shall now explain the activity:

In each round, **each participant, A and B, will start with 3,000 pesos**.

In each round, Participant A should decide how many of the 3,000 pesos s/he wishes **to keep and how much to transfer to** Participant B**.** In other words, Participant A may transfer all the 3,000 pesos to Participant B, transfer nothing, or any amount between 0 and 3,000 pesos.

In each round, the researchers will **multiply the money that** Participant A **transfers to** Participant B **by three.**

Participant B will therefore end up with the 3,000 pesos s/he started the round with, as well as **three times the amount transferred by** Participant A.

Of this amount, Participant B should decide how much s/he wishes to return to Participant A.

Participant B decides whether s/he wishes to return something, everything or nothing to Participant A**.**

Once this decision has been made the round finishes and we move on to the next one.

After the six rounds have been completed, we will add up your winnings, which will be **handed over in cash to each participant.**

**(RESEARCHER SHOWS POSTER 2)**

**Don’t worry if you don’t understand – later on we are going to provide some examples and do some practice rounds.**

**Next, we will explain how** to keep a record of your decisions and of the amount of money you win in each round.

Later on we will define who will be Participants A and who will be Participants B.

To help you learn how to use the record sheet we will now **go through three examples**.

**Remember that these are simply examples.** In each round you are free to decide how much money you want to keep and how much to transfer. You can transfer any amount of money between 0 and 3,000 pesos.

We will begin now with the **first** **example.**

**In the first example** we will see how Participant A should fill in the record sheet**.**

**(THE RESEARCHER SHOWS THE POSTER OF THE RECORD SHEET FOR PARTICIPANT A. KEEP EXAMPLE 2 COVERED)**

Remember that at the beginning of each round, each Participant **begins with 3,000 pesos**. This amount is written in the first column.

Participant A should decide how many of these **3,000** pesos s/he wishes to keep and how many s/he wants to transfer to Participant B.

**Suppose that Participant A decides** to keep **500 pesos and to transfer 2,500** pesos to **Participant B.** The amount left **(500 pesos**) should be written in the **second** **column,** “Amount I am left with,” and the amount transferred to Participant B **(2,500 pesos)** should be written in the **third column,** “Amount transferred by Participant A to Participant B.”

The money **received by** Participant B is multiplied by 3, and Participant B decides if s/he wants to return something, everything or nothing to Participant A**.**

Suppose that Participant B decides to return **4,000 pesos** to Participant A. This amount **should be written in the** **column** “Amount returned by Participant B to Participant A.”

At the end of the round, Participant A will have **4,500 pesos: the 500** pesos s/he retained **plus the 4,000 pesos** returned by Participant B. This should be written in the **fifth** **column** “Final winnings,” **which records the sum of the second and the fourth columns. In this case, final winnings are 4,500 pesos.**

**Does anyone have any questions? If you do, please raise your hand and a researcher will answer them one to one.**

Now let us look at how Participants B **should** fill in the record sheet for the **first example.**

**(THE RESEARCHER SHOWS THE POSTER OF THE RECORD SHEET FOR EXAMPLE 1 FOR PARTICIPANT B. KEEP EXAMPLE 2 COVERED)**

As in the case of Participant A, **3,000** pesos is recorded in the **first column** of the record sheet. This is the initial amount with which **each participant starts every round.**

The “Amount transferred by Participant A to Participant B” should be written in the **second column**. In our example, Participant A transfers **2,500** pesos to Participant B.

The money **received by** Participant B is multiplied by 3. That is, in this example, Participant B **receives 7,500 pesos (2,500 pesos x 3 = 7,500 pesos**). This amount should be written in the **third column**, “Amount transferred by Participant A to Participant B, x three.”

Thus, Participant B would end up with **10,500 pesos (**the original **3,000** pesos plus **7,500** pesos transferred by Participant A). This amount should be written in the **fourth column** “My winnings following the transfer from Participant A.” That is, **10,500 pesos.**

Of these **10,500 pesos**, Participant B decides whether s/he wishes to **return something, everything or nothing** to Participant A**.**

Suppose that Participant B decides to return 4,000 pesos to Participant A**. This amount should be written in the fifth column “Amount returned by Participant B to Participant A.”**

At the end of the round, Participant B will have **6,500** pesos, calculated by subtracting the “Amount returned by Participant B to Participant A” **(4,000 pesos**) from “My winnings following the transfer from Participant A” **(10,500 pesos**). This amount should be recorded in the **sixth column** “Final winnings.” In this **case the final winnings are 6,500 pesos (10,500 pesos minus 4,000 pesos).**

**Does anyone have any questions? If you do, please raise your hand and a researcher will answer them one to one.**

**Let us examine a second example:** First, we are going to see how to fill in the record sheet for Participant A**.**

**(THE RESEARCHER SHOWS THE POSTER OF THE RECORD SHEET FOR EXAMPLE 2 FOR PARTICIPANT A. KEEP EXAMPLE 1 COVERED)**

Remember that each participant starts every round with **3,000 pesos**. This amount is recorded in the **first column** of the record sheet.

Participant A should decide how much of the **3,000 pesos** s/he wishes to keep and how much to transfer to Participant B.

**Suppose that Participant A decides** to keep **2,000 pesos and to transfer 1,000** pesos to Participant B**. The remaining amount (2,000 pesos) should be noted in the second column,** “Amount I am left with,” while the amount transferred to Participant B **(1,000 pesos**) should be registered in the **third column**, “Amount transferred by Participant A to Participant B.”

The money **received by** Participant B is multiplied by 3, and Participant B decides if s/he wants to return something, everything or nothing to Participant A**.**

Suppose that Participant B decides to return **500 pesos** to Participant A. This amount should be registered in the **fourth column,** “Amount returned by Participant B to Participant A.”

At the end of the round, Participant A will have **2,500 pesos:** the **2,000 pesos** s/he retained **plus 500 pesos** returned by Participant B. This amount, which is the sum of the second and the fourth columns should be registered in the **fifth column,** “Final winnings.” **In this case the final winnings are 2,500 pesos.**

Now we are going to see how to fill in the record sheet for **Participant B for the second example.**

**(THE RESEARCHER SHOWS THE POSTER OF THE RECORD SHEET FOR EXAMPLE 2 FOR PARTICIPANT B. KEEP EXAMPLE 1 COVERED)**

As in Participant A’s record sheet, **3,000** pesos - the initial amount with which **each participant starts every round** - is recorded in the **first column** of the record sheet.

The “Amount transferred by Participant A to Participant B” should be written in the **second column**. In our example, Participant A transfers **1,000** pesos to Participant B**.**

The money **received by** Participant B is multiplied by 3. That is, in this example, Participant B **receives 3,000 pesos (1,000 pesos x 3 = 3,000 pesos).** This amount should be written in the **third column**, “Amount transferred by Participant A to Participant B, x three.”

Thus, Participant B would end up with **6,000 pesos** (the original 3,000 pesos plus **3,000** pesos transferred by Participant A). This amount should be written in the **fourth column** “My winnings following the transfer from Participant A.” That is, **6,000 pesos**

Of these **6,000 pesos**, Participant B **decides whether s/he wishes to return something, everything or nothing to** Participant A**.**

Suppose that Participant B decides to return **500 pesos** to Participant A**. This amount should be registered in the fifth column** “Amount returned by Participant B to Participant A.”

At the end of the round, Participant B will have **5,500** pesos, calculated by subtracting “My winnings following the transfer from Participant A” **(500 pesos**) from the “Amount returned by Participant B to Participant A.” **(6,000 pesos**). This amount should be noted in the **sixth column** “Final winnings.” In this **case the final winnings are 5,500 pesos (6,000 pesos minus 500 pesos).**

**Does anyone have any questions? If you do, please raise your hand and a researcher will answer them one to one.**

So, to summarize: (**RESEARCHER SHOWS POSTER 2 AGAIN)**

**Remember:** The greater the amount Participant A transfers to Participant B, the greater the amount that is tripled and that Participant B receives.

In any case, Participant A is free to decide how much to transfer to Participant B, just as Participant B is free to decide how much s/he wishes to return to Participant A.

**Does anyone have any questions? If you do, please raise your hand and a researcher will answer them one to one.**

**Don’t worry if you don’t understand – later on we are going to provide some examples and carry out some practice rounds.**

Before starting we are going to hand out a sheet of paper containing some questions we would like you to answer, in order to ensure that **you have understood the** **instructions**. This is not the exercise, so you still do not need to take any decisions. Remember that you should not speak with anyone during the activity. When you finish please raise your hand and a researcher will come over and check your answers.Please answer the questions now.

**(RESEARCHER DISTRIBUTES THE QUESTIONS)**

Please fill in the blank space with the correct answer:

Remember that at the start of each round Participant A and B both start off with 3,000 pesos.

Participant A decides to transfer 1,700 pesos to Participant B.

**How much money is** Participant A **left with?** _1,300 pesos____

The amount Participant B receives is multiplied by three by the researchers.

That is, Participant B receives 5,100 pesos.

**Therefore,** Participant B **would end up with**: 8.100 pesos____

Next, Participant B decides to return 2,500 pesos to Participant A.

**What are** Participant A’s **final winnings?** _3,800 pesos______

**What are** Participant B’s **final winnings?** _5,600 pesos______

**(THE RESEARCHER COLLECTS AND CHECKS EACH SET OF ANSWERS. WHEN AN ANSWER IS INCORRECT THE RESEARCHER EXPLAINS BRIEFLY WHY. IF, FOLLOWING THE EXPLANATION, S/HE SUSPECTS THAT THE PERSON DOES NOT FULLY UNDERSTAND THE REASONS THIS SHOULD BE NOTED ON THE OBSERVATIONS SHEET)**

At this stage participants will find out whether they have been given the role of participant A or B. **This bag contains cards marked either with the letter A or the letter B**. Each letter also has an identification number assigned it.

A researcher hands round the bag and the **participants choose a card at random.** Please do not show your card to anyone else, nor make any comments to the other participants.

**(WAIT WHILE PARTICIPANTS ARE ASSIGNED THE LETTER A OR B)**

Remember that you will keep your letter throughout the entire activity.

At this stage Participants B **should leave the room** and wait in the room next door. Please take your personal belongings and identification numbers with you. Remember, you should not speak to anyone.

**(ONE OF THE RESEARCHERES WILL GO WITH PARTICIPANTS B INTO THE NEXT ROOM TO ENSURE THEY DO NOT SPEAK)**

**Instructions for Participants A, practice round**

The people left in the room are Participants A. **Please check that your card says A**.

We are going to have **two practice rounds**that will not count towards your total winnings but that provide an important opportunity to practice the activity.

Right now you are receiving your record sheet. **Please do not write anything until we ask you to.**

**(RESEARCHER HANDS OUT RECORD SHEETS)**

You are also receiving a sheet of paper that is divided into three sections, **like this poster.**

We will now explain how to communicate your decisions to Participants B.

**(RESEARCHER HANDS OUT SHEETS AND SHOWS POSTER OF TRANSFER FORM)**

You, Participants A, **should only fill in the first** section. The second part will be completed by the researcher and third will be filled in by Participant B. On the **left of the form it says who should fill in the form.**

**We are now going to explain the first section:**

First, you should write down the number of the round, in this case P1, and your identification number – that is, the number on your card. **(THE RESEARCHER SHOULD WAIT UNTIL THE PARTICIPANTS HAVE COMPLETED THEIR SHEETS). Please do this now.**

Next, each Participant A should decide **how much of the 3,000 pesos s/he wishes to keep and how much s/he wishes to transfer to** Participant B**.** S/he should write this amount in the blank space above, following the phrase “Participant A transfers.”

**Please write down now** how much of the 3,000 pesos you wish to transfer to Participant B.

In addition, please write on your **record sheet**:

- The “Amount I am left with” in the second column.
- The “Amount transferred by Participant A to Participant B” in the third column.

When you finish, raise your hand. One of the researchers will collect your sheet.

**(THE RESEARCHERS SHOULD WAIT UNTIL THE PARTICIPANTS HAVE COMPLETED THEIR FORMS AND THEN COLLECT THEM, CONFIRMING THAT THE PARTICIAPNTS HAVE RECORDED THE SAME AMOUNT ON THEIR RECORD SHEET)**

We have now received all the decisions made by Participants A. We shall now go to the room where Participants B are waiting. **Each Participant B chooses an envelope at random to decide which Participant A s/he will play with this round. (SHOW THE ENVELOPES)**

**While Participants B** **are choosing they should speak with no one.**

Remember that in each round you will play with the same partner you played within the previous round or with someone new. **This will be announced at the start of each round.**

**(THE RESEARCHER NOW FILLS OUT THE SECOND PART OF THE TRANSFER FORM SO S/HE CAN BE TAKEN TO THE ROOM WHERE PARTICIPANTS B ARE WAITING)**

**Instructions for Participants A at end of the practice round.**

Participants B have now made their decision.

Please place your card on the table, with its identification number in plain view, so that Participant B will know what you have decided.

The decision made by Participant B appears lower down the sheet, in the section that says “Participant B returns…”

Write this amount in the fourth column of your record sheet where it says “Amount returned by Participant B to Participant A” and record your final winnings in the fifth column of the record sheet.

When you finish, raise your hand. One of the researchers will collect your sheet.

**(THE RESEARCHER COLLECTS THE TRANSFER FORM FOR THE PRACTICE ROUND AND DISTRIBUTES THE NEW TRANSFER FORM)**

We have now completed the first practice round.

**Instructions for Participants A, Practice round 2**

We are about to begin the second practice round.

You will be playing with the **same partner you played with in the last round.**

On the new sheet, you should first write the number of the round and your identification number, which appears on your card. **(RESEARCHERS SHOULD WAIT UNTIL PARTICIPANTS COMPLETE THEIR SHEET)**

Now please write down how much of the 3,000 pesos you wish to retain and how much you wish to transfer to Participant B. Please also write on your record sheet:

- The Amount I am left with” in the second column
- The “Amount transferred by Participant A to Participant B” in the third column

After taking the decision please raise your hand. One of the researchers will collect your sheet.

**(RESEARCHERS SHOULD WAIT UNTIL THE PARTICIPANTS HAVE COMPLETED THEIR FORMS AND THEN COLLECT THEM, CONFIRMING THAT THEY HAVE RECORDED THE SAME AMOUNT ON THEIR RECORD SHEET)**

We have now received all the decisions made by Participants A. We shall now go to the room where Participants B are waiting.

While Participants B are taking their decision **they should speak with no one.**

**(THE RESEARCHER NOW FILLS OUT THE SECOND PART OF THE TRANSFER FORM SO THEY CAN BE TAKEN TO THE ROOM WHERE PARTICIPANTS B ARE)**

**Instructions for Participants A at the end of practice round 2**

Participants B have now all made their decisions.

Please place your card on the table, with its identification number in plain view, so that the decision made by Participant B is clear.

The decision made by Participant B appears lower down the sheet in the section that says “Participant B returns…”

Write this amount in the fourth column of your record sheet where it says “Amount returned by Participant B to Participant A” and record your final winnings in the fifth column of the record sheet.

When you finish, raise your hand. One of the researchers will collect your sheet.

**(THE RESEARCHER COLLECTS THE TRANSFER SHEET FOR ROUND 2 AND DISTRIBUTES THE NEW TRANSFER SHEET)**

We have now completed practice round 2.

**Instructions for** Participants A, **Round 1**

We are now about to start Round 1. From this point onwards, records will be kept of your winnings.

In this round you will **play with a new partner**.

On the new sheet, you should first note down the number of the round and your identification number. (**THE RESEARCHER SHOULD WAIT UNTIL THE PARTICIPANTS HAVE COMPLETED THEIR SHEETS).**

**Please write down now** how much of the 3,000 pesos you wish to transfer to Participant B.

In addition, please write on your **record sheet**:

- The “Amount I am left with” in the second column
- The “Amount transferred by Participant A to Participant B” In the third column

When you finish, raise your hand. One of the researchers will collect your sheet.

In this round you will play with a new partner.

**(RESEARCHERS SHOULD WAIT UNTIL THE PARTICIPANTS HAVE COMPLETED THEIR FORMS AND THEN COLLECT THEM, CONFIRMING THAT THEY HAVE RECORDED THE SAME AMOUNT ON THEIR RECORD SHEET)**

We have now received all the decisions made by Participants A. We shall now go to the room where Participants B are waiting. **Each** Participant B **chooses an envelope at random to decide which Participant A s/he will play with during this round.**

**Please do not speak to the other participants** while Participants B are choosing.

**(THE RESEARCHER NOW FILLS OUT THE SECOND PART OF THE TRANSFER FORM SO S/HE CAN BE TAKEN TO THE ROOM WHERE PARTICIPANTS B ARE)**

**Instructions for Participants A at the end of round 1**

The Participants B have now made their decision.

Please place your card on the table, with its identification number in plain view, so that the decision made by Participant B is clear.

The decisions of Participants B appear lower down the sheet in the section that says “Participant B returns…”

This amount should be written in the **fourth column** “My winnings following the transfer from Participant A” and record your final winnings in the fifth column of the record sheet.

When you finish, raise your hand. One of the researchers will collect your sheet.

**(THE RESEARCHER COLLECTS THE TRANSFER SHEET FOR ROUND 1 AND DISTRIBUTES THE NEW TRANSFER SHEET)**

We have now completed Round 1.

**Instructions for Participants A, Round 2**

We are now going to begin Round 2.

You will be playing with the **same partner as in the previous round.**

On the new sheet, you should first write the number of the round and your identification number, which appears on the sheet. **(RESEARCHERS SHOULD WAIT UNTIL PARTICIPANTS COMPLETE THEIR SHEET)**

Now please write down how much of the 3,000 pesos you wish to retain and how much you wish to transfer to Participant B. Please also write on your record sheet:

- The Amount I am left with” in the second column
- The “Amount transferred by Participant A to Participant B” in the third column

After taking the decision please raise your hand. One of the researchers will collect your sheet.

**(RESEARCHERS SHOULD WAIT UNTIL PARTICIPANTS COMPLETE THEIR SHEET)**

Please note down how much of the 3,000 pesos you want to keep and how much to transfer to Participant B. Also, please note on the record sheet:

- The “Amount I am left with” in the second column
- The “Amount transferred by Participant A to Participant B” in the third column

After taking the decision please raise your hand. One of the researchers will collect your sheet.

**(THE RESEARCHERS SHOULD WAIT UNTIL THE PARTICIPANTS HAVE COMPLETED THEIR FORMS AND THEN COLLECT THEM, CONFIRMING THAT THEY HAVE RECORDED THE SAME AMOUNT ON THEIR RECORD SHEET)**

We have now received all the decisions made by Participants A. We shall now go to the room where Participants B are waiting.

**Please do not speak to the other participants** while Participants B are choosing.

**(THE RESEARCHER NOW FILLS OUT THE SECOND PART OF THE TRANSFER FORM SO S/HE CAN BE TAKEN TO THE ROOM WHERE PARTICIPANTS B ARE WAITING)**

**Instructions for Participants A at the end of Round 2**

The Participants B have now made their decisions.

Please place your card on the table, with its identification number in plain view, so that the decision made by Participant B is clear.

The decisions of Participants B appears on the lower part of the sheet, in the section that says “Participant B returns…”

Write this amount in the fourth column of your record sheet where it says “Amount returned by Participant B to Participant A” and record your final winnings in the fifth column of the record sheet.

When you finish, raise your hand. One of the researchers will collect your sheet.

**(THE RESEARCHER COLLECTS THE TRANSFER FORM FOR THE PRACTICE ROUND AND DISTRIBUTES THE NEW TRANSFER FORM)**

We have now completed Round 2.

**Instructions for Participants A, Round 3**

We are now going to begin Round 2.

You will be playing with the **same partner as in the previous round.**

On the new sheet, you should first write the number of the round and your identification number, which appears on the sheet. **(RESEARCHERS SHOULD WAIT UNTIL PARTICIPANTS COMPLETE THEIR SHEET)**

Now please write down how much of the 3,000 pesos you wish to retain and how much you wish to transfer to Participant B. Please also write these amounts on the record sheet

After taking the decision please raise your hand. One of the researchers will collect your sheet.

**(RESEARCHERS SHOULD WAIT UNTIL THE PARTICIPANTS HAVE COMPLETED THEIR FORMS AND THEN COLLECT THEM, CONFIRMING THAT THEY HAVE RECORDED THE SAME AMOUNT ON THEIR RECORD SHEET)**

We have now received all the decisions made by Participants A. We shall now go to the room where Participants B are waiting.

**Please do not speak to the other participants** while Participants B are choosing.

**(THE RESEARCHER NOW FILLS OUT THE SECOND PART OF THE TRANSFER FORM SO S/HE CAN BE TAKEN TO THE ROOM WHERE PARTICIPANTS B ARE WAITING)**

**Instructions for Participants A at the end of Round 3**

The Participants B have now made their decisions.

The decisions of Participants B appears on the lower part of the sheet, in the section that says “Participant B returns…”

Write this amount in the fourth column of your record sheet where it says “Amount returned by Participant B to Participant A” and record your final winnings in the fifth column of the record sheet.

When you finish, raise your hand. One of the researchers will collect your sheet.

**(THE RESEARCHER COLLECTS THE TRANSFER FORM FOR THE PRACTICE ROUND AND DISTRIBUTES THE NEW TRANSFER FORM)**

We have now completed Round 3.

**Instructions for Participants A, Round 4**

We are now going to begin Round 4.

You will be playing with the same partner as in the previous round.

On the new sheet, you should first write the number of the round and your identification number, which appears on the sheet. **(RESEARCHERS SHOULD WAIT UNTIL PARTICIPANTS COMPLETE THEIR SHEET)**

Now please write down how much you wish to retain and how much you wish to transfer to Participant B. Please also write on your record sheet.

After taking the decision please raise your hand. One of the researchers will collect your sheet.

**(RESEARCHERS SHOULD WAIT UNTIL THE PARTICIPANTS HAVE COMPLETED THEIR FORMS AND THEN COLLECT THEM, CONFIRMING THAT THEY HAVE RECORDED THE SAME AMOUNT ON THEIR RECORD SHEET)**

We have now received all the decisions made by Participants A. We shall now go to the room where Participants B are waiting.

**Please do not speak to the other participants** while Participants B are choosing.

**(THE RESEARCHER NOW FILLS OUT THE SECOND PART OF THE TRANSFER FORM SO S/HE CAN BE TAKEN TO THE ROOM WHERE PARTICIPANTS B ARE WAITING)**

**Instructions for Participants A at the end of Round 4**

The Participants B have now made their decisions.

The decisions of Participants B appears on the lower part of the sheet, in the section that says “Participant B returns…”

Write this amount in the fourth column of your record sheet where it says “Amount returned by Participant B to Participant A” and record your final winnings in the fifth column of the record sheet.

When you finish, raise your hand. One of the researchers will collect your sheet.

**(THE RESEARCHER COLLECTS THE TRANSFER FORM FOR THE PRACTICE ROUND AND DISTRIBUTES THE NEW TRANSFER FORM)**

We have now completed Round 4.

**Instructions for Participants A, Round 5**

We are now going to begin Round 5.

You will be playing with the **same partner as in the previous round.**

On the new sheet, you should first write the number of the round and your identification number, which appears on the sheet. **(RESEARCHERS SHOULD WAIT UNTIL PARTICIPANTS COMPLETE THEIR SHEET)**

Now please write down how much of the 3,000 pesos you wish to retain and how much you wish to transfer to Participant B. Please also write on your record sheet.

After taking the decision please raise your hand. One of the researchers will collect your sheet.

**(THE RESEARCHERS SHOULD WAIT UNTIL THE PARTICIPANTS HAVE COMPLETED THEIR FORMS AND THEN COLLECT THEM, CONFIRMING THAT THEY HAVE RECORDED THE SAME AMOUNT ON THEIR RECORD SHEET)**

We have now received all the decisions made by Participants A. We shall now go to the room where Participants B are waiting.

**Please do not speak to the other participants** while Participants B are choosing.

**(THE RESEARCHER NOW FILLS OUT THE SECOND PART OF THE TRANSFER FORM SO S/HE CAN BE TAKEN TO THE ROOM WHERE PARTICIPANTS B ARE WAITING)**

**Instructions for Participants A at the end of Round 5**

The Participants B have now made their decisions.

The decisions of Participants B appears on the lower part of the sheet, in the section that says “Participant B returns…”

Write this amount in the fourth column of your record sheet where it says “Amount returned by Participant B to Participant A” and record your final winnings in the fifth column of the record sheet.

When you finish, raise your hand. One of the researchers will collect your sheet.

**(THE RESEARCHER COLLECTS THE TRANSFER FORM FOR THE PRACTICE ROUND AND DISTRIBUTES THE NEW TRANSFER FORM)**

We have now completed Round 5.

**Instructions for Participants A, Round 6**

We are now going to begin Round 6.

In this round you will be playing with a **new partner.**

On the new sheet, you should first write the number of the round and your identification number, which appears on the sheet. **(RESEARCHERS SHOULD WAIT UNTIL PARTICIPANTS COMPLETE THEIR SHEET)**

Now please write down how much you wish to retain and how much you wish to transfer to Participant B. Please also write these amounts on your record sheet

After taking the decision please raise your hand. One of the researchers will collect your sheet.

**(THE RESEARCHERS SHOULD WAIT UNTIL THE PARTICIPANTS HAVE COMPLETED THEIR FORMS AND THEN COLLECT THEM, CONFIRMING THAT THEY HAVE RECORDED THE SAME AMOUNT ON THEIR RECORD SHEET)**

We have now received all the decisions made by Participants A. We shall now go to the room where Participants B are waiting.

**Each Participant B chooses an envelope at random to decide which Participant A s/he will play with during this round.**

**Please do not speak to the other participants** while Participants B are choosing.

**(THE RESEARCHER NOW FILLS OUT THE SECOND PART OF THE TRANSFER FORM SO S/HE CAN BE TAKEN TO THE ROOM WHERE PARTICIPANTS B ARE WAITING)**

**Instructions for Participants A at the end of Round 6**

The Participants B have now made their decisions.

The decisions of Participants B appears on the lower part of the sheet, in the section that says “Participant B returns…”

Write this amount in the fourth column of your record sheet and record your final winnings in the fifth column of the record sheet.

When you finish, raise your hand. One of the researchers will collect your sheet.

**(THE RESEARCHER COLLECTS THE TRANSFER FORM FOR ROUND 6)**

We have now completed Round 6. The activity has ended. Please do not speak with anyone. A researcher will now collect your record sheets and ask you some questions. While you are answering these questions another researcher will calculate your winnings. We will call participants up individually and hand over their winnings in cash.

**(THE RESEARCHER COLLECTS THE RECORD SHEETS BUT NOT THE SHEETS WITH LETTERS ON)**
