# Supplementary material for: Prosocial Behavior and Subjective Insecurity in Violent Contexts: Field Experiments
Source: PLoS One. 2016 Jul 29;11(7):e0158878. doi: 10.1371/journal.pone.0158878 (PMC4966936; doi:10.1371/journal.pone.0158878)
Supplement: S6 Text — (DOCX) [file pone.0158878.s015.docx]

**Instructions trust_ B**

Good morning / afternoon,

Thank you for accepting our invitation to take part in this activity. Today’s activity is part of a research project that is being carried out by researchers at *Universidad de los Andes*. The funding for this project comes from an international institution. Today there are *x* researchers working on the following activities: *xxx*, *xxx*, and *xxx*.

Before starting the activity, we are going to give all of you $10,000 to contribute towards your transport costs. This is your money, so please keep it.

**(RESEARCHER GIVES OUT MONEY TO EACH PARTICIPANT)**

We will now begin to read the instructions in order to explain the activity in which you will be taking part.

The objective of this activity is to find out how people make decisions; thus, we will learn by observing you decide. All the decisions that you make during this activity, as well as any other information that you provide us with, will remain confidential. The only people with access to the information will be the researchers working on this project; it will not be given to any one else.

You can earn money by taking part in this activity. The amount of money that you earn depends on the decisions you make as well on the decisions that others make. We do not know the exact amount that you will earn; however, it will be somewhere between **$0 and $72,000** pesos**.**

The total amount of money that you earn will be rounded up or down to the closest $1,000; for example, if you earn $18,400 pesos or less, the figure will be rounded down to $18,000, and if you earn $18,500 pesos or more, the figure will be rounded up to $19,000 pesos.

Does everyone understand?

We will use money in this activity because as in real life, your decisions will have economic consequences. The money that you earn in today’s activity is yours, and only the researchers will know the exact amount that you have earned.

This activity may be different to other activities in which members of your community have participated. As such, any comment or explication that you may have heard regarding today’s activity may well not be pertinent.

**Today’s activity will last three hours.** Your participation is completely voluntary and you are free to withdraw at any moment. However, if you do withdraw other participants will also be forced to withdraw as we need an equal number of participants. In order to receive the money that you have earned, you will have to stay until the end of the activity. **If anyone is unable to stay until the end of the activity, please advise us now.**

**Is anyone unable to stay for the full three hours?**

This activity will not involve any risks. On the contrary, it could benefit you by allowing you the opportunity to earn money. Are you willing to participate?

If you are indeed willing, please read and sign the form that the researcher is currently handing out. **(RESEARCHER HANDS OUT INFORMED CONSENT SHEETS)**

**(RESEARCHER READS INFORMED CONSENT SHEETS)**

**INFORMED CONSENT FORM** Date: ____________________

You have been invited to participate in this exercise, which is part of a wider scientific research project.

This activity will not involve any risk. On the contrary, it could be beneficial, by giving you the opportunity to earn money. The amount of money you earn will depend on your decisions as well as on the decisions made by others. At the end of the activity you will be required to answer some questions. The amount of money that earn during the exercise as well as the decisions you make will remain private. Your decision to participate is completely voluntary. You are free to withdraw from the activity at any moment. However, if you decide to withdraw, you will not receive any of the money.

I, ___________________________________________ declare that I understand the previously stated information as well as my rights and commitments as part of this activity. I am also aware that I can withdraw at any moment and waiver my right to claim any money I have earned.

Signed, ________________________________National ID #____________

I, **Lina Moros**, lecturer at *Universidad de los Andes*, hereby certify that this information will be used responsibly for academic and educative purposes. I also certify that each participant will be given the sum of money that they have earned during the exercise.

Signed, ________________________________National ID #____________

We are now going to read the instructions in order for you to participate in the activity. **Please do not speak to other participants.** If you speak to others you will disrupt the activity and make it more difficult for others to understand the instructions. You may also ruin the activity and it will have to be cancelled. We also request that you turn your phones on to silent so that the activity is not interrupted.

Please pay attention to these instructions.

The activity in which you are about to participate **will be carried out in pairs**. Each person will be playing with someone else who will be selected randomly. However, no one will know the identity of the person with whom they are playing; only the researchers will know who the pairs are.

**You will play six rounds.** In each round you will either play with the same partner as in the previous round or with a new partner; we will tell you which at the beginning of each round.

Each pair will be made up of a **participant A and a participant B**. What letter participant you are will be determined by a draw. Each participant will also be given an identification number.

From this moment onwards you should speak to no one. If anyone has a question, then please raise your hand. Under no circumstances ask the question aloud. Any questions will be individually answered. **(RESEARCHER TO SHOW POSTER 1)**

I shall now explain the activity:

In each round, **each participant, A and B, will start with 3,000 pesos**.

In each round, Participant A should decide how many of the 3,000 pesos s/he wishes **to keep and how much to transfer to** Participant B**.** In other words, Participant A may transfer all the 3,000 pesos to Participant B, transfer nothing, or any amount between 0 and 3,000 pesos.

In each round, the researchers will **multiply the money that** Participant A **transfers to** Participant B **by three.**

Participant B will therefore end up with the 3,000 pesos s/he started the round with, as well as **three times the amount transferred by** Participant A.

Of this amount, Participant B should decide how much s/he wishes to return to Participant A.

Participant B decides whether s/he wishes to return something, everything or nothing to Participant A**.**

Once this decision has been made the round finishes and we move on to the next one.

After the six rounds have been completed, we will add up your winnings, which will be **handed over in cash to each participant.**

**(RESEARCHER SHOWS POSTER 2)**

**Don’t worry if you don’t understand – later on we are going to provide some examples and do some practice rounds.**

**Next, we will explain how** to keep a record of your decisions and of the amount of money you win in each round.

Later on we will define who will be Participants A and who will be Participants B.

To help you learn how to use the record sheet we will now **go through three examples**.

**Remember that these are simply examples.** In each round you are free to decide how much money you want to keep and how much to transfer. You can transfer any amount of money between 0 and 3,000 pesos.

We will begin now with the **first** **example.**

**In the first example** we will see how Participant A should fill in the record sheet**.**

**(THE RESEARCHER SHOWS THE POSTER OF THE RECORD SHEET FOR PARTICIPANT A. KEEP EXAMPLE 2 COVERED)**

Remember that at the beginning of each round, each Participant **begins with 3,000 pesos**. This amount is written in the first column.

Participant A should decide how many of these **3,000** pesos s/he wishes to keep and how many s/he wants to transfer to Participant B.

**Suppose that Participant A decides** to keep **500 pesos and to transfer 2,500** pesos to **Participant B.** The amount left **(500 pesos**) should be written in the **second** **column,** “Amount I am left with,” and the amount transferred to Participant B **(2,500 pesos)** should be written in the **third column,** “Amount transferred by Participant A to Participant B.”

The money **received by** Participant B is multiplied by 3, and Participant B decides if s/he wants to return something, everything or nothing to Participant A**.**

Suppose that Participant B decides to return **4,000 pesos** to Participant A. This amount **should be written in the** **column** “Amount returned by Participant B to Participant A.”

At the end of the round, Participant A will have **4,500 pesos: the 500** pesos s/he retained **plus the 4,000 pesos** returned by Participant B. This should be written in the **fifth** **column** “Final winnings,” **which records the sum of the second and the fourth columns. In this case, final winnings are 4,500 pesos.**

**Does anyone have any questions? If you do, please raise your hand and a researcher will answer them one to one.**

Now let us look at how Participants B **should** fill in the record sheet for the **first example.**

**(THE RESEARCHER SHOWS THE POSTER OF THE RECORD SHEET FOR EXAMPLE 1 FOR PARTICIPANT B. KEEP EXAMPLE 2 COVERED)**

As in the case of Participant A, **3,000** pesos is recorded in the **first column** of the record sheet. This is the initial amount with which **each participant starts every round.**

The “Amount transferred by Participant A to Participant B” should be written in the **second column**. In our example, Participant A transfers **2,500** pesos to Participant B.

The money **received by** Participant B is multiplied by 3. That is, in this example, Participant B **receives 7,500 pesos (2,500 pesos x 3 = 7,500 pesos**). This amount should be written in the **third column**, “Amount transferred by Participant A to Participant B, x three.”

Thus, Participant B would end up with **10,500 pesos (**the original **3,000** pesos plus **7,500** pesos transferred by Participant A). This amount should be written in the **fourth column** “My winnings following the transfer from Participant A.” That is, **10,500 pesos.**

Of these **10,500 pesos**, Participant B decides whether s/he wishes to **return something, everything or nothing** to Participant A**.**

Suppose that Participant B decides to return 4,000 pesos to Participant A**. This amount should be written in the fifth column “Amount returned by Participant B to Participant A.”**

At the end of the round, Participant B will have **6,500** pesos, calculated by subtracting the “Amount returned by Participant B to Participant A” **(4,000 pesos**) from “My winnings following the transfer from Participant A” **(10,500 pesos**). This amount should be recorded in the **sixth column** “Final winnings.” In this **case the final winnings are 6,500 pesos (10,500 pesos minus 4,000 pesos).**

**Does anyone have any questions? If you do, please raise your hand and a researcher will answer them one to one.**

**Let us examine a second example:** First, we are going to see how to fill in the record sheet for Participant A**.**

**(THE RESEARCHER SHOWS THE POSTER OF THE RECORD SHEET FOR EXAMPLE 2 FOR PARTICIPANT A. KEEP EXAMPLE 1 COVERED)**

Remember that each participant starts every round with **3,000 pesos**. This amount is recorded in the **first column** of the record sheet.

Participant A should decide how much of the **3,000 pesos** s/he wishes to keep and how much to transfer to Participant B.

**Suppose that Participant A decides** to keep **2,000 pesos and to transfer 1,000** pesos to Participant B**. The remaining amount (2,000 pesos) should be noted in the second column,** “Amount I am left with,” while the amount transferred to Participant B **(1,000 pesos**) should be registered in the **third column**, “Amount transferred by Participant A to Participant B.”

The money **received by** Participant B is multiplied by 3, and Participant B decides if s/he wants to return something, everything or nothing to Participant A**.**

Suppose that Participant B decides to return **500 pesos** to Participant A. This amount should be registered in the **fourth column,** “Amount returned by Participant B to Participant A.”

At the end of the round, Participant A will have **2,500 pesos:** the **2,000 pesos** s/he retained **plus 500 pesos** returned by Participant B. This amount, which is the sum of the second and the fourth columns should be registered in the **fifth column,** “Final winnings.” **In this case the final winnings are 2,500 pesos.**

Now we are going to see how to fill in the record sheet for **Participant B for the second example.**

**(THE RESEARCHER SHOWS THE POSTER OF THE RECORD SHEET FOR EXAMPLE 2 FOR PARTICIPANT B. KEEP EXAMPLE 1 COVERED)**

As in Participant A’s record sheet, **3,000** pesos - the initial amount with which **each participant starts every round** - is recorded in the **first column** of the record sheet.

The “Amount transferred by Participant A to Participant B” should be written in the **second column**. In our example, Participant A transfers **1,000** pesos to Participant B**.**

The money **received by** Participant B is multiplied by 3. That is, in this example, Participant B **receives 3,000 pesos (1,000 pesos x 3 = 3,000 pesos).** This amount should be written in the **third column**, “Amount transferred by Participant A to Participant B, x three.”

Thus, Participant B would end up with **6,000 pesos** (the original 3,000 pesos plus **3,000** pesos transferred by Participant A). This amount should be written in the **fourth column** “My winnings following the transfer from Participant A.” That is, **6,000 pesos**

Of these **6,000 pesos**, Participant B **decides whether s/he wishes to return something, everything or nothing to** Participant A**.**

Suppose that Participant B decides to return **500 pesos** to Participant A**. This amount should be registered in the fifth column** “Amount returned by Participant B to Participant A.”

At the end of the round, Participant B will have **5,500** pesos, calculated by subtracting “My winnings following the transfer from Participant A” **(500 pesos**) from the “Amount returned by Participant B to Participant A.” **(6,000 pesos**). This amount should be noted in the **sixth column** “Final winnings.” In this **case the final winnings are 5,500 pesos (6,000 pesos minus 500 pesos).**

**Does anyone have any questions? If you do, please raise your hand and a researcher will answer them one to one.**

So, to summarize: (**RESEARCHER SHOWS POSTER 2 AGAIN)**

**Remember:** The greater the amount Participant A transfers to Participant B, the greater the amount that is tripled and that Participant B receives.

In any case, Participant A is free to decide how much to transfer to Participant B, just as Participant B is free to decide how much s/he wishes to return to Participant A.

**Does anyone have any questions? If you do, please raise your hand and a researcher will answer them one to one.**

**Don’t worry if you don’t understand – later on we are going to provide some examples and carry out some practice rounds.**

Before starting we are going to hand out a sheet of paper containing some questions we would like you to answer, in order to ensure that **you have understood the** **instructions**. This is not the exercise, so you still do not need to take any decisions. Remember that you should not speak with anyone during the activity. When you finish please raise your hand and a researcher will come over and check your answers.Please answer the questions now.

**(RESEARCHER DISTRIBUTES THE QUESTIONS)**

Please fill in the blank space with the correct answer:

Remember that at the start of each round Participant A and B both start off with 3,000 pesos.

Participant A decides to transfer 1,700 pesos to Participant B.

**How much money is** Participant A **left with?** _1,300 pesos____

The amount Participant B receives is multiplied by three by the researchers.

That is, Participant B receives 5,100 pesos.

**Therefore,** Participant B **would end up with**: 8.100 pesos____

Next, Participant B decides to return 2,500 pesos to Participant A.

**What are** Participant A’s **final winnings?** _3,800 pesos______

**What are** Participant B’s **final winnings?** _5,600 pesos______

**(THE RESEARCHER COLLECTS AND CHECKS EACH SET OF ANSWERS. WHEN AN ANSWER IS INCORRECT THE RESEARCHER EXPLAINS BRIEFLY WHY. IF, FOLLOWING THE EXPLANATION, S/HE SUSPECTS THAT THE PERSON DOES NOT FULLY UNDERSTAND THE REASONS THIS SHOULD BE NOTED ON THE OBSERVATIONS SHEET)**

At this stage participants will find out whether they have been given the role of participant A or B. **This bag contains cards marked either with the letter A or the letter B**. Each letter also has an identification number assigned it.

A researcher hands round the bag and the **participants choose a card at random.** Please do not show your card to anyone else, nor make any comments to the other participants.

**(WAIT WHILE PARTICIPANTS ARE ASSIGNED THE LETTER A OR B)**

Remember that you will keep your letter throughout the entire activity.

At this stage Participants B **should leave the room** and wait in the room next door. Please take your personal belongings and identification numbers with you. Remember, you should not speak to anyone.

**(ONE OF THE RESEARCHERES WILL GO WITH PARTICIPANTS B INTO THE NEXT ROOM TO ENSURE THEY DO NOT SPEAK)**

**Instructions for participants B in practice round 1**

The people in this room are participants B. **Please check that the letter B is written on your card.**

We are going to wait in silence while participants A make their decisions.

We will now have a practice round that will not count towards your final earnings. It is, however, important to have a practice run of the activity.

You will now receive the balance sheets. **Please do not write anything until we say so.**

**(RESEARCHER HANDS OUT BALANCE SHEETS)**

The decisions made by participants A are in these envelopes. **Please choose an envelope at random to find out with which participant A you will be playing in this practice round**. Please do not open the envelopes until we give the instruction to do so.

**(RESEARCHER GOES AROUND TO EACH PARTICIPANT ALLOWING THEM TO CHOOSE AN ENVELOPE)**

**(RESEARCHER: WHEN EVERYONE HAS CHOSEN AN ENVELOPE, INDICATE THAT THEY ARE NOW ALLOWED TO OPEN IT)**

We will now explain how you can communicate your decision to participant A.

The sheet that you have just received is divided into three parts, as is shown in this poster.

**(RESEARCHER TO PRESENT THE POSTER THAT SHOWS THE SENDING FORMAT)**

In the top part of the sheet, **participant A made her/his decision about how much they wanted to send to participant B**. In the second part, we multiply this number by three.

Therefore, in this round, you have received the amount that participant A gave you (multiplied by three), plus the $3,000 pesos that you originally had at the beginning of the round.

Please remember that you should fill out **the third part of the sheet that** you have just been given. You should first, however, write your identification number on the sheet, which is the number written on your card. Please write it now.

**(RESEARCHER WAITS FOR THE PARTICIPNTS TO FINISH FILLING IN THEIR SHEETS).**

Next, each participant B should write the total amount of money that they have after this round in the blank space. They should do so after the phrase: participant B’s total amount of money…

This amount is calculated by the researcher and is written in the second part of the sheet.

Please now write the amount of money that you have after this round in the blank space, after the phrase: participant B’s total amount of money…

**(RESEARCHER WAITS UNTIL THE PARTICIPANTS HAVE FILLED IN THEIR SHEETS)**

You should next decide **how much money you want to return to participant A**. You should write this amount in the blank space, after the phrase: participant B will return…

Please also write on your balance sheet:

- The **“amount given by participant A to participant B”** in the second column.
- The **“amount given by participant A to participant B multiplied by three”** in the third column.
- **“My earnings after giving money to participant A”** in the forth column.
- **“Amount participant B gave back to participant A”** in the fifth column.

When you have finished, please raise your hand (do not put the sheet in the envelope). A researcher will collect both the sheet and the envelope from you.

**(RESEARCHER WAITS UNTIL THE PARTICIPANTS HAVE FILLED OUT THEIR SHEET AND THEN COLLECT THEM, CHECKING THAT THEY HAVE WRITTEN THE SAME AMOUNT ON THE BALANCE SHEET)**

We now have all the decisions made by participants B. We will now go to the room in which participants A are waiting and inform them of your decisions.

Please **do not speak to any of the other participants.**

Please remember that in the next round you will either be playing with the same partner with whom you played the previous round or with a new partner; we will tell you which at the beginning of each round.

We have now finished practice round one.

**(REASEARCHER TAKES THE SHEETS TO THE ROOM IN WHICH PARTICIPANTS A ARE LOCATED AND GIVES THEM TO THE CORRESPONDING PARTNER)**

**Instructions for participants B in practice round 2**

We are going to wait in silence until participants A have made their decision. We will now begin practice round 2.

You will be playing with the same partner as in the previous round. Please place the card with its number visible on the table. A researcher will give you the decision made by participant A. Please wait until the researcher has finished handing out the sheets before making your decision.

**(RESEARCHER GOES TO EACH TABLE TO DELIVER EACH PARTICIPANT HER OR HIS SHEET)**

At the top of the sheet, participant A made her/his decision about how much they wanted to give to participant B. In the second part of the sheet, you should multiply this amount by three.

Therefore, in this round, you will earn the amount that was given to you by participant A (multiplied by three) plus the $3,000 pesos that you began the round with.

Please remember that you should fill out **the third part of the sheet** that you have just received.

However, before doing this, please write your **identification number** on the sheet**, which is the number written on your card. Please write this number now.**

**(RESEARCHER WAITS UNTIL ALL PARTICIPANTS HAVE FILLED OUT THEIR SHEETS)**

Following this, each participant should write the total number of money that they have after this round in the blank space, following the phrase: participant B’s total amount of money…

**(RESEARCHER WAITS UNTIL THE PARTICIPANTS HAVE FILLED IN THEIR SHEETS)**

You should next decide **how much money you want to return to participant A**. You should write this amount in the blank space, after the phrase: participant B will return…

Please also write on your balance sheet:

- The **“amount given by participant A to participant B”** in the second column.
- The **“amount given by participant A to participant B multiplied by three”** in the third column.
- **“My earnings after giving money to participant A”** in the forth column.
- **“Amount participant B gave back to participant A”** in the fifth column.

When you have finished, please raise your hand (do not put the sheet in the envelope). A researcher will collect both the sheet and the envelope from you.

**(WAIT)**

We now have all the decisions made by participants B. We will now go to the room in which participants A are waiting and inform them of your decisions.

Please **do not speak to any of the other participants.**

Please remember that in the next round you will either be playing with the same partner with whom you played the previous round or with a new partner; we will tell you which at the beginning of each round.

We have now finished practice round two.

**I**

**nstructions for participants B in round 1**

We are going to wait in silence while participants A make their decisions. We will now begin round one. From this round onwards, the money you earn will be recorded.

**In this round you will play with a new partner.**

The decisions made by participants A are in these envelopes. **Please choose an envelope at random to find out with which participant A you will be playing in round one**.

Please do not open the envelopes until we give the instruction to do so.

**(RESEARCHER GOES AROUND TO EACH PARTICIPANT ALLOWING THEM TO CHOOSE AN ENVELOPE)**

**(RESEARCHER WHEN EVERYONE HAS CHOSEN AN ENVELOPE, INDICATE THAT THEY ARE NOW ALLOWED TO OPEN IT)**

You can now open the envelope and take out the sheet inside.

In the top part of the sheet, **participant A made her/his decision about how much they wanted to send to participant B**. In the second part, we multiply this number by three.

Therefore, in this round, you have received the amount that participant A gave you (multiplied by three), plus the $3,000 pesos that you originally had at the beginning of the round.

Please remember that you should fill out **the third part of the sheet that** you have just been given. You should first, however, write your **identification number** on the sheet**, which is the number written on your card. Please write it now.**

**(RESEARCHER WAITS UNTIL THE PARTICIPANTS HAVE FILLED IN THEIR SHEETS)**

Next, each participant B should write the total amount of money that they have after this round in the blank space, after the phrase: participant B’s total amount of money…

This amount is calculated by the researcher and is written in the second part of the sheet.

Please now write the amount of money that you have after this round in the blank space, after the phrase: participant B’s total amount of money…

**(RESEARCHER WAITS UNTIL THE PARTICIPANTS HAVE FILLED IN THEIR SHEETS)**

You should next decide **how much money you want to return to participant A**. You should write this amount in the blank space after the phrase: participant B will return…

Please also write on your balance sheet:

- The **“amount given by participant A to participant B”** in the second column.
- The **“amount given by participant A to participant B multiplied by three”** in the third column.
- **“My earnings after giving money to participant A”** in the forth column.
- **“Amount participant B gave back to participant A”** in the fifth column.

When you have finished, please raise your hand (do not put the sheet in the envelope). A researcher will collect both the sheet and the envelope from you.

**(WAIT)**

We now have all the decisions made by participants B. We will now go to the room in which participants A are waiting and inform them of your decisions.

Please **do not speak to any of the other participants.**

Please remember that in the next round you will either be playing with the same partner with whom you played the previous round or with a new partner; we will tell you which at the beginning of each round.

We have now finished round one.

**Instructions for participants B in round 2**

We are going to wait in silence while participants A make their decisions. We will now begin round two.

In this round, you will be playing with the same partner as in round one. Please place the card with its number visible on the table. A researcher will give you the decision made by participant A. Please wait until the researcher has finished handing out the sheets until making your decision.

**(RESEARCHER GOES AROUND HANDING OUT EACH PARTICIPANT’S SHEET)**

In the top part of the sheet, participant **A made her/his decision about how much s/he wanted to send to participant B**. In the second part, we multiply this number by three.

Therefore, in this round, you have received the amount that participant A gave you (multiplied by three), plus the $3,000 pesos that you originally had at the beginning of the round.

Please remember that you should fill out **the third part of the sheet that** you have just been given. You should first, however, write your **identification number** on the sheet**, which is the number written on your card. Please write it now.**

**(RESEARCHER WAITS UNTIL THE PARTICIPANTS HAVE FILLED IN THEIR SHEETS)**

Next, each participant B should write the total amount of money that they have after this round in the blank space, after the phrase: participant B’s total amount of money…

This amount is calculated by the researcher and is written in the second part of the sheet.

Please now write the amount of money that you have after this round in the blank space, after the phrase: participant B’s total amount of money…

**(RESEARCHER WAITS UNTIL THE PARTICIPANTS HAVE FILLED IN THEIR SHEETS)**

You should next decide **how much money you want to return to participant A**. You should write this amount in the blank space after the phrase: participant B will return…

Please also write on your balance sheet:

- The **“amount given by participant A to participant B”** in the second column.
- The **“amount given by participant A to participant B multiplied by three”** in the third column.
- **“My earnings after giving money to participant A”** in the forth column.
- **“Amount participant B gave back to participant A”** in the fifth column.

When you have finished, please raise your hand (do not put the sheet in the envelope). A researcher will collect both the sheet and the envelope from you.

**(WAIT)**

We now have all the decisions made by participants B. We will now go to the room in which participants A are waiting and inform them of your decisions.

Please **do not speak to any of the other participants.**

Please remember that in the **next round you will either be playing with the same partner with whom you played the previous round or with a new partner**; we will tell you which at the beginning of each round.

We have now finished round two.

**Instructions for participants B in round three**

We are going to wait in silence while participants A make their decisions.

We will now begin round three.

In this round, you will be playing with the same partner as in the previous round. A researcher will give you the decision made by participant A. Please wait until the researcher has finished handing out the sheets until making your decision. Please place the card with its number visible on the table.

**(RESEARCHER GOES AROUND HANDING OUT EACH PARTICIPANT’S SHEET)**

Please now write the amount of money that you have after this round in the blank space after the phrase: participant B’s total amount of money…

You should next decide **how much money you want to return to participant A**. You should write this amount in the blank space after the phrase: participant B will return…

Please also write on your balance sheet:

- The **“amount given by participant A to participant B”** in the second column.
- The **“amount given by participant A to participant B multiplied by three”** in the third column.
- **“My earnings after giving money to participant A”** in the forth column.
- **“Amount participant B gave back to participant A”** in the fifth column.

When you have finished, please raise your hand (do not put the sheet in the envelope). A researcher will collect both the sheet and the envelope from you.

**(RESEARCHER WAITS UNTIL THE PARTICIPANTS HAVE FILLED OUT THEIR SHEET AND THEN COLLECT THEM, CHECKING THAT THEY HAVE WRITTEN THE SAME AMOUNT ON THE BALANCE SHEET)**

We now have all participant B’s decisions. We will now go to the room in which participants A are waiting and inform them of your decisions.

Please **do not speak to any of the other participants.**

We have now finished round three.

**Instructions for participants B in round 4**

We will now begin round four.

In this round, you will be playing with the same partner as in the previous round. A researcher will give you the decision made by participant A. Please wait until the researcher has finished handing out the sheets until making your decision. Please place the card with its number visible on the table.

**(RESEARCHER GOES AROUND HANDING OUT EACH PARTICIPANT’S SHEET)**

Please write the amount of money that you now have after this round in the blank space, after the phrase: participant B’s total amount of money…

You should next decide **how much money you want to return to participant A**. You should write this amount in the blank space, after the phrase: participant B will return…

Please also write on your balance sheet:

- The **“amount given by participant A to participant B”** in the second column.
- The **“amount given by participant A to participant B multiplied by three”** in the third column.
- **“My earnings after giving money to participant A”** in the forth column.
- **“Amount participant B gave back to participant A”** in the fifth column.

When you have finished, please raise your hand (do not put the sheet in the envelope). A researcher will collect both the sheet and the envelope from you.

**(WAIT)**

We now have all the decisions made by participants B. We will now go to the room in which participants A are waiting and inform them of your decisions.

Please **do not speak to any of the other participants.**

We have now finished round four.

**Instructions for participants B in round 5**

We will now begin round five.

In this round, you will be playing with the same partner as in round one. A researcher will give you the decision made by participant A. Please wait until the researcher has finished handing out the sheets until making your decision. Please place the card with its number visible on the table.

**(RESEARCHER GOES AROUND HANDING OUT EACH PARTICIPANT’S SHEET)**

Please now write the amount of money that you have after this round in the blank space, after the phrase: participant B’s total amount of money…

You should next decide **how much money you want to return to participant A**. You should write this amount in the blank space, after the phrase: participant B will return…

Please also write on your balance sheet:

- The **“amount given by participant A to participant B”** in the second column.
- The **“amount given by participant A to participant B multiplied by three”** in the third column.
- **“My earnings after giving money to participant A”** in the forth column.
- **“Amount participant B gave back to participant A”** in the fifth column.

When you have finished please raise your hand (do not put the sheet in the envelope). A researcher will collect both the sheet and the envelope from you.

**(RESEARCHER WAITS UNTIL THE PARTICIPANTS HAVE FILLED OUT THEIR SHEET AND THEN COLLECT THEM, CHECKING THAT THEY HAVE WRITTEN THE SAME AMOUNT ON THE BALANCE SHEET)**

We now have all the decisions made by participants B. We will now go to the room in which participants A are waiting and inform them of your decisions.

Please **do not speak to any of the other participants.**

We have now finished round five.

**Instructions for participants B in round 6**

We will now begin round six.

In this round you will be playing with a new partner. These envelopes contain the decisions made by two participants A. **Please choose an envelope at random to find out with which participant A you will be playing with in this round.** Please do not open the envelope until told to do so.

**(RESEARCHER GOES AROUND ALLOWING THE PARTICIPANTS TO CHOOSE AN ENVELOPE)**

**(RESEARHER: WHEN ALL PARTICIPANTS HAVE CHOSEN AN ENVELOPE INFORM THEM THAT THEY CAN NOW OPEN THE ENVELOPE)**

You can now open the envelope and take out the sheet inside.

Please now write the amount of money that you have after this round in the blank space after the phrase: participant B’s total amount of money…

You should next decide **how much money you want to return to participant A**. You should write this amount in the blank space after the phrase: participant B will return…

Please also write on your balance sheet:

- The **“amount given by participant A to participant B”** in the second column.
- The **“amount given by participant A to participant B multiplied by three”** in the third column.
- **“My earnings after giving money to participant A”** in the forth column.
- **“Amount participant B gave back to participant A”** in the fifth column.

When you have finished please raise your hand (do not put the sheet in the envelope). A researcher will collect both the sheet and the envelope from you.

**(RESEARCHER WAITS UNTIL THE PARTICIPANTS HAVE FILLED OUT THEIR SHEET AND THEN COLLECTS THEM, CHECKING THAT THEY HAVE WRITTEN THE SAME AMOUNT ON THE BALANCE SHEET)**

We now have all the decisions made by participants B. We will now go to the room in which participants A are waiting and inform them of your decisions.

Please **do not speak to any of the other participants.**

**Instructions for participants B at the end of round 6**

We have now finished round six, and the exercise is over. Please do not speak to anybody. A researcher will come and collect your balance sheet and will ask a few questions. While you are answering the questions, the researcher will calculate your earnings. After answering all the questions, we will call each individual and give them their earnings in cash.

**(THE RESEARCHER COLLECTS THE BALANCE SHEETS BUT NOT THE CARDS CONTAINING THE LETTER A OR B)**
