# Supplementary material for: Prosocial Behavior and Subjective Insecurity in Violent Contexts: Field Experiments
Source: PLoS One. 2016 Jul 29;11(7):e0158878. doi: 10.1371/journal.pone.0158878 (PMC4966936; doi:10.1371/journal.pone.0158878)
Supplement: S8 Text — (DOCX) [file pone.0158878.s017.docx]

**Dictator B**

**Instructions**

**(RESEARCHER HANDS OUT INFORMED CONSENT FORMS AT THE ENTRANCE)**

Good morning / afternoon,

Thank you for accepting our invitation to take part in this activity. Today’s activity is part of a research project that is being carried out by researchers at *Universidad de los Andes*. The funding for this project comes from an international institution. Today there are *x* researchers working on the following activities: *xxx*, *xxx*, and *xxx*.

Before starting the event, we are going to give all of you $10,000 to contribute towards your transport costs. This is your money, so please keep it.

**(RESEARCHER GIVES OUT MONEY TO EACH PARTICIPANT)**

We will now begin to read the instructions in order to explain the activity in which you will be taking part.

The objective of this activity is to find out how people make decisions; thus, we will learn by observing you decide. All the decisions that you make during this activity, as well as any other information that you provide us with, will remain confidential. The only people with access to the information will be the researchers working on this project; it will not be given to any one else.

You can earn money by taking part in this activity. The amount of money that you earn depends on the decisions you make as well on the decisions that others make. We do not know the exact amount that you will earn; however, it will be somewhere between **$0 and $60,000 pesos**.

The total amount of money that you earn will be rounded up or down to the closest $1,000; for example, if you earn $18,400 pesos or less, the figure will be rounded down to $18,000, and if you earn $18,500 pesos or more, the figure will be rounded up to $19,000 pesos.

Does everyone understand?

We will use money in this activity because as in real life, your decisions will have economic consequences. The money that you earn in today’s activity is yours, and only the researchers will know the exact amount that you have earned.

This activity may be different to other activities in which members of your community have participated. As such, any comment or explication that you may have heard regarding today’s activity may well not be pertinent.

**Today’s activity will last two hours.** Your participation is completely voluntary and you are free to withdraw at any moment. However, if you do withdraw, other participants will also be forced to withdraw as we need an equal number of participants. In order to receive the money that you have earned, you will have to stay until the end of the activity. **If anyone is unable to stay for the full two hours, please advise us now.**

**Is anyone unable to stay for the full three hours?**

This activity will not involve any risks. On the contrary, it could benefit you by allowing you the opportunity to earn money. Are you willing to participate?

If you are indeed willing, please read and sign the form that the researcher is currently handing out. **(RESEARCHER HANDS OUT INFORMED CONSENT SHEETS)**

**(RESEARCHER READS INFORMED CONSENT FORM)**

**INFORMED CONSENT FORM** Date: ___________

You have been invited to participate in this exercise, which is part of a wider scientific research project.

This activity will not involve any risk. On the contrary, it could be beneficial, by giving you the opportunity to earn money. The amount of money you earn will depend on your decisions as well as on the decisions made by others. At the end of the activity you will be required to answer a survey. The amount of money that you earn during the exercise as well as the decisions you make will remain private. Your decision to participate is completely voluntary. You are free to withdraw from the activity at any moment. However, if you decide to withdraw, you will not receive any of the money.

AGREEMENT: (Please write in print)

I, ________________ write name __________ declare that I understand the previously stated information as well as my rights and commitments as part of this activity. I am also aware that I can withdraw at any moment and waiver my right to claim any money I have earned.

Signed, _______ Please sign form __________c.c.

I, Lina Moros, researcher at *Universidad de los Andes*, hereby certify that this information will be used responsibly for academic and educative purposes. I also certify that each participant will be given the sum of money that they have earned during the exercise.

Signed, _______________________________________________ c.c.

The researcher will now collect your informed consent forms.

We are now going to read the instructions in order for you to participate in the activity. **Please do not speak to other participants.** If you speak to others you will disrupt the activity and make it more difficult for others to understand the instructions. You may also ruin the activity and it will have to be cancelled. **We also request that you turn your phones on to silent** so that the activity is not interrupted.

Please pay attention to these instructions.

The activity in which you are about to participate **will be carried out in pairs**. Each person will be playing with someone else who will be selected **randomly**. However, no one will know the identity of the person with whom they are playing; only the researchers **will know who the pairs are**.

Each pair will be made up of a **participant A and a participant B**. What letter participant you are will be determined by a draw. Each participant will also be given an identification number.

From this moment onwards you should speak to no one. If anyone has a question, then please raise your hand. Under no circumstances ask the question aloud. Any questions will be individually answered. **(RESEARCHER SHOWS POSTER 1)**

We will now explain the activity:

Participant A will receive $60,000 pesos.

With this money, participant A needs to decide what amount of **money s/he wants to keep and what** amount s/he wants to give to participant B. Participant A can give participant B everything, nothing, or any amount between $0 and $60,000 pesos.

Participant B does **not have to make any decisions**.

Participant B’s earnings will be the **amount of money that** was given by participant A. Participant A’s earnings will be her/his original $60,000 pesos less the amount that s/he decides to give to participant B.

**All earnings will be given out at the end of the activity.**

**Does anybody have any questions? If so, please raise your hand and a researcher will resolve the issue individually.**

We will now illustrate with some examples to show you how the activity works:

**(RESEARCHER EXPLAINS EACH EXAMPLE USING THE EXAMPLES POSTER)**

1) Let’s suppose that participant A decides to send $10,000 pesos of her/his $60,000 pesos to participant B: participant B will earn $10,000 pesos and participant A $50,000 pesos ($60,000 pesos - $10,000 pesos = $50,000 pesos). The activity will then be over.

2) Let’s suppose that participant A decides to send $1,000 pesos to participant B from her/his original $60,000 pesos: **participant B will earn $1,000** pesos and participant A $59,000 pesos ($60,000 pesos - $1,000 pesos = $59,000 pesos). The activity will then be over.

3) Let’s suppose that participant A decides to send $22,000 pesos to participant B from her/his original $60,000 pesos: participant B will earn $22,000 pesos and **participant A $38,000 pesos** ($60,000 pesos - $22,000 pesos = $38,000 pesos). The activity will then be over.

**Please remember that these are examples.** Participant A is free to choose what amount of money s/he wants to keep and what amount s/he wants to send to participant B. Participant A can send participant B everything, nothing, or any amount **between** **$0 and $60,000 pesos.**

**Does anybody have any questions? If so, please raise your hand and a researcher will resolve the issue individually.**

Before beginning the activity, we are going to hand out a sheet containing some questions to make sure that **you have correctly understood the instructions**. This is not the activity itself, and you are still not required to make any decisions. Please remember that you should not talk to anybody during the activity. When you have finished answering the questions please raise your hand and a researcher will make sure that you have correctly answered the questions.

**(RESEARCHER GIVES OUT QUESTION SHEETS)**

Please fill in the blanks with the correct answer.

Remember that participant A is given $60,000 pesos.

Let’s suppose that participant A decides to give participant B $19,000 pesos:

**What are participant A’s final earnings?** $_41,000______pesos

**What are participant B’s final earnings?** $_19,000______pesos

**THE RESEARCHER WILL COLLECT AND INDIVIDUALLY CHECK THAT ALL THE QUESTIONS HAVE BEEN ANSWERED CORRECTLY. IF SOMEONE ANSWERS A QUESTION INCORRECTLY, THEN THEY WILL RECEIVE A BRIEF EXPLANATION OF WHY IT IS INCORRECT. IF AFTER EXPLAINING, THERE IS A SUSPICIONS THAT THE PERSON STILL DOES NOT UNDERSTAND THE ANSWER, THEN A NOTE WILL BE MADE ON AN OBSERVATION SHEET)**

People will then be assigned as either participant A or participant B. There are cards with the letters A and B in the bag. Each card also has its own identification number.

A researcher **will then go round** with the bag, allowing each participant to choose a card at random. Please do not show your card to anyone else or make comments to the other participants.

**(THE RESEARCHER GOES AROUND WITH THE BAG)**

**(THE RESEARCHER WAITS UNTIL ALL THE CARDS HAVE BEEN CHOSEN AT RANDOM)**

Participants B should now leave the room and take a seat in the next room.

Please take with you your personal belongings and your identification number.

**Remember that you should not talk to any of the other participants.**

**(A RESEARCHER GOES WITH PARTICIPANTS B TO THE OTHER ROOM TO MAKE SURE THAT THERE IS NO TALKING)**

**Instructions for participants B**

The people in this room **are participants B. Please take a moment to make sure that the letter B is written on you card.**

**We are going to wait in silence until participants A have made their decisions.**

The decisions made by participants A are in these envelopes. Please **choose an envelope** at random to find out with which participant A you will be playing. Please do not open the envelopes until we give the instruction to do so.

**(RESEARCHER GOES AROUND TO EACH PARTICIPANT ALLOWING THEM TO CHOOSE AN ENVELOPE)**

You can now open the envelope and take out the sheet.

Participant A’s decision about how much they sent participant B is written at the top of the sheet. Your earnings will be based on this amount.

**(RESEARCHER PRESENTS THE POSTER THAT SHOWS THE SENDING FORMAT)**

On the bottom part of the sheet please write your identification number, **which is the number written on your card. Please write it down now.**

Write down your earnings in the blank space where it says participant B’s earnings. Please remember that your earnings are the amount given by participant A.

When you have finished, place the sheet back in the envelope and raise your hand. A researcher will collect the envelope. **Please do not speak to any other participants.**

**(WHEN ALL PARTICIPANTS HAVE FINISHED, THE RESEARCHER WILL COLLECT THE ENVELOPES, BUT NOT THE CARDS. THEY ARE THEN TO BE TAKEN TO THE ROOM IN WHICH PARTICIPANTS A ARE WAITING)**

**Instructions for the end of the activity in both participant A and participant B’s rooms**

We have now finished the activity. Please do not speak to any of the other participants. A researcher will come to you to ask you some questions. While you are answering the questions, a researcher will calculate your earnings. When we have finished asking the questions, each person will be called **to collect their earnings in cash**.
